# Patient-centered access to mental health care at selected life-course stages: a scoping review using the Levesque framework

**DOI:** 10.1186/s12913-026-15895-z

**Published:** 2026-10-22

**Authors:** Jan Christopher Cwik, Moritz Hess, Claudia Kardys, Thomas Plieger, Annika Walker, Maria Weyermann, Jessica Frieß, Luisa Hilbrand, Ben Rohrbach, Bernhard Breil

**Affiliations:** 1 Faculty of Health Care, Hochschule Niederrhein, Reinarzstraße 49, 47805 Krefeld, Germany; 2 Faculty of Social Work, Hochschule Niederrhein, Mönchengladbach, Germany; 3 Nutrition Communication and Public Health, Faculty of Nutritional Sciences, Hochschule Niederrhein, Mönchengladbach, Germany; 4 Faculty of Health Care, Hochschule Niederrhein, Krefeld, Germany; 5 Epidemiology and Biometry, Faculty of Health Care, Hochschule Niederrhein, Krefeld, Germany; 6 Research Funding Officer, Division of Research and Knowledge Transfer, Hochschule Niederrhein, Krefeld, Germany; 7 Research Assistant, Faculty of Health Care, Hochschule Niederrhein, Krefeld, Germany; 8 Faculty of Health Care, Hochschule Niederrhein, Krefeld, Germany; 9 Faculty of Health Care, Hochschule Niederrhein, Krefeld, Germany

**Keywords:** Mental health services, Access to care, Patient-centered access, Levesque framework, Life course, Perinatal mental health, Child and adolescent mental health, Older adults, Scoping review, Health equity

## Abstract

**Background:**

The Levesque patient-centered access framework links five service dimensions with five corresponding population abilities, from recognizing need to engaging with care. We synthesized its substantive applications to mental-health access among pregnant and postpartum people, children and adolescents aged 0–17 years, and adults aged 65 years or older.

**Methods:**

We conducted a framework-guided scoping review with descriptive evidence mapping and narrative synthesis. Four Elicit reports supported semantic retrieval, preliminary screening, and draft extraction; PubMed and Web of Science searches supplemented identification through 1 August 2026. One reviewer determined eligibility, evidence roles, and framework coding; a second independently checked extracted data against full texts. Two reviewers independently appraised empirical studies and reviews using MMAT 2018 and the JBI review checklist. Direct population-specific evidence was distinguished from adjacent and contextual sources. Reporting was guided by PRISMA-ScR and GAMER.

**Results:**

Reported screening totals were 367 records and 70 full texts. The final map contained 37 sources: 23 empirical studies, 11 reviews, one protocol, and two conceptual sources. Ten sources provided direct evidence: five perinatal sources (three empirical studies and two reviews), four youth empirical studies, and one older-adult empirical study. The other 27 sources did not support direct cross-population claims. Preconference appraisal agreement was 95.2% (297/312 item pairs; descriptive pooled κ = 0.895). Appraisal identified limitations in representativeness, confounder control, mixed-methods integration, and review procedures. Reported barriers included difficulties before formal service contact, alongside problems in referral navigation, cultural acceptability, workforce capacity, affordability, and continuity. Gatekeepers and service interfaces varied across life-course stages. Validated measurement linking service dimensions and population abilities was limited. Incomplete screening documentation restricted auditability.

**Conclusions:**

The framework offers a comparative vocabulary for examining access pathways and population-specific mechanisms. Findings from this small, heterogeneous evidence base are exploratory, not estimates of barrier prevalence or relative importance. Research should strengthen paired measurement, age-specific reporting, longitudinal designs, and pathway-level intervention evaluation.

**Clinical trial number:**

not applicable.

**Supplementary Information:**

The online version contains supplementary material available at https://doi.org/10.1186/s12913-026-15895-z.

## Introduction

Access to mental health care is not a single event. It is a process that begins before an individual recognizes symptoms as a potential need for care and continues through help-seeking, service contact, affordability, continuity, and meaningful engagement. Traditional metrics that count utilization or facility density fail to capture these stages. Foundational access theory stems from Penchansky and Thomas [1], who described access as the degree of fit between clients and health care systems. Later, Gulliford et al. [2] refined this by distinguishing among available services, barriers to accessing them, the relevance and effectiveness of services, and the needs and perspectives of diverse groups. The Andersen Behavioral Model [3] further expanded access theory by distinguishing predisposing, enabling, and need-related determinants of health care utilization and by differentiating potential from realized access. Building on this lineage, Levesque et al. [4] proposed a patient-centered model linking five supply-side dimensions (approachability, acceptability, availability/accommodation, affordability, appropriateness) with corresponding demand-side abilities (perceive, seek, reach, pay, engage).

The framework is especially relevant to mental health because barriers often arise simultaneously on both sides of the care interface. On the population side, symptoms may be normalized, hidden by stigma, interpreted through cultural idioms, or deprioritized because of competing family, work, school, or caregiving demands. On the system side, services may be difficult to identify, poorly coordinated, geographically remote, unaffordable, culturally mismatched, or insufficiently tailored to complex needs. Mental health care thus requires a model that captures not only whether services exist but also whether people perceive the need for care, can seek it safely, can reach it in practice, can pay for it, and remain engaged [5–11].

The Levesque framework was selected because the review concerns the complete access pathway, extending beyond realized service utilization. In contrast to models centered primarily on determinants or utilization, it explicitly pairs five service characteristics with five corresponding population abilities and traces access from recognition of need through help-seeking, reaching and paying for care, to meaningful engagement. This paired structure permits comparison of system-side and population-side mechanisms while retaining population-specific differences in autonomy, gatekeeping, service interfaces, and continuity requirements.

Evidence from a broader scoping review of empirical applications supports using the Levesque framework as an organizing structure for access research. Across 31 empirical health care access studies, the framework was applied in diverse settings and services, including a priori use for instrument development and a posteriori (post hoc) use for organizing and interpreting collected access data [12]. These applications position the framework as a practical bridge between health-system characteristics and population abilities, and provide methodological context for the present review.

A mental-health-focused mapping review by Lowther-Payne et al. [13] makes this measurement gap tangible. Synthesizing 152 UK adult mental health service studies, the review used the Levesque framework to classify access measures and barriers and found that most studies measured health care utilization, whereas very few addressed perception of need, desire for care, or consequences of access [13]. It also identified frequent barriers to reaching and seeking care, including availability, stigma and discrimination, trust, and appropriateness, and cautioned that routine service data can obscure inequalities when demographic fields are incomplete [13]. It therefore provides a mental-health-specific methodological benchmark used exclusively for contextual interpretation.

To our knowledge, no synthesis has compared Levesque-informed mental-health access at selected life-course stages using a common set of pathway-oriented questions. Using this common framework, the present review examines where barriers arise along the access pathway, which actors or institutions mediate progression to the next stage, how similarly labeled barriers acquire different meanings across populations, and which components of the Levesque framework remain underoperationalized.

The three populations—pregnant and postpartum people, children and adolescents, and older adults—were specified a priori in the preregistered protocol and were retained as purposively selected cases representing contrasting configurations of dependence, autonomy, gatekeeping, and service-system contact at different life stages. Perinatal access is commonly mediated through maternity, primary-care, and mental-health services [14–16]; access in childhood and adolescence frequently depends on caregivers, schools, and confidentiality-sensitive services [7, 8]; and access in later life may require coordination across primary, geriatric, mental-health, community, and social care [9, 17–19]. The comparison is illustrative. It does not cover all populations facing access barriers or imply that the selected groups are uniquely or necessarily most vulnerable. Migration, disability, socioeconomic disadvantage, race and ethnicity, gender diversity, and rurality were treated as intersecting access determinants where reported.

The present review examined four questions:

1. How has the Levesque framework been used in mental-health access research?
2. At which stage of the access pathway do reported barriers and facilitators arise?
3. Which actors, gatekeepers, service-system interfaces, and population-specific acceptability conditions shape progression and sustained engagement?
4. Which methodological and framework-use gaps remain?

## Methods

### Design

We conducted a framework-guided scoping review with descriptive evidence mapping and narrative synthesis. Scoping reviews can examine the application of a conceptual framework and describe characteristics and findings across heterogeneous sources [20]. Evidence mapping organizes the volume, distribution, characteristics, and gaps of the evidence base [21, 22]. The approaches overlap, and both require transparent and systematic procedures [21]. The scoping-review designation reflects our questions about framework application and access-pathway mechanisms; evidence mapping describes the organization of the findings. The PRISMA Extension for Scoping Reviews (PRISMA-ScR) guided reporting [23].

The Levesque framework was selected at the start of study planning and included in the preregistration of 6 February 2026. The three target populations and descriptive framework-based synthesis were also prespecified. The present article maps substantive framework applications and distinguishes direct, adjacent and contextual evidence. A common conceptual framework enables comparison of access stages, mediating mechanisms and framework operationalization. The scope was defined by the prespecified framework and populations; additional searches strengthened evidence identification without changing the descriptive purpose of the review. Supplementary Material S2 relates the registered plan to the implemented search, selection and synthesis procedures. The review does not claim comprehensive coverage of all mental-health-access literature.

### Source reports and searches

Elicit, an AI-assisted literature-search and extraction tool, was selected for a rapid, broad overview of potentially relevant literature, with conventional searches planned to extend coverage. A four-case comparison found that Elicit identified additional eligible studies but missed many relevant records, supporting its use for preliminary and supplementary searching [24]. Three reports dated 18 February 2026 each ranked up to 1,000 query-level records: perinatal, older-adult, and broader mental-health searches, the latter filtered for children and adolescents. They generated 31, 17 and two structured extraction outputs, respectively (50 total). A dedicated youth report dated 31 July 2026 applied the prespecified 0–17-year population definition and substantive framework-use criterion. It ranked 5,000 records, advanced 20 candidates to Elicit-supported full-text screening and proposed three sources for extraction. These are platform outputs, not unique, deduplicated human-screening counts.

JCC reviewed all 20 candidate records advanced by the youth-focused report. Of the ten records for which Elicit lacked full-text access, six were considered potentially relevant based on title and abstract and were subsequently retrieved and assessed. None met all criteria for direct evidence; three were retained as adjacent evidence, and three were excluded. The remaining four records were excluded during human title and abstract screening and were therefore not sought for retrieval. No report sought for retrieval remained unavailable. The exact youth-focused research question, screening criteria, extraction instructions, generated report, and available source-level decisions, together with the available documentation for the three original reports, are documented across Supplementary Materials S4–S5.

JCC accessed PubMed’s “Cited by” links for the framework article on 28 July 2026; this yielded no additional candidate records. Web of Science Core Collection (A&HCI, ESCI, SCI-EXPANDED, SSCI) citation tracking on 1 August identified 2,423 records; population filters yielded 185 youth, 48 perinatal and 67 older-adult hits (300 before deduplication). Separately, the thematic PubMed update on 28 July combined framework, population, mental-health and access terms with 2026/02/18:3000[crdt] for record creation date. Neither of its two hits met all direct-evidence criteria. One was retained as adjacent evidence in the final map and also identified through the youth-focused Elicit and citation routes; the other was excluded. Overlapping route contributions are distinguished in Supplementary Material S5. Supplementary Material S5 provides the available queries and histories; the total PubMed citing-record set is not documented in the available materials.

The reported aggregate screening flow comprised 372 candidate-record entries before deduplication: 50 outputs from the original Elicit reports, all 20 candidates from the dedicated youth report, 300 Web of Science population-filter hits, and two thematic PubMed records. The three Elicit extraction proposals were a subset of the 20 youth candidates and were not counted again. The reported deduplication and screening totals were five duplicates removed, 367 unique records screened by JCC at title and abstract level, and 297 excluded. All 70 reports sought were retrieved. JCC assessed them in full text and excluded 33 for not meeting the eligibility criteria, leaving 37 sources in the evidence map. No complete record-level screening log was maintained. The available source-specific decisions in Supplementary Material S5 form a partial retrospective account; they do not document every exclusion or permit a complete breakdown of exclusion reasons. Final eligibility, evidence classification, Levesque coding and synthesis decisions were based on the original publications.

Two framework-level reviews were retained exclusively for methodological contextualization. Cu et al. informed the interpretation of how the Levesque framework has been applied in empirical health-care-access research, whereas Lowther-Payne et al. provided a mental-health-specific benchmark for the operationalization of access stages and measures in adult services [12, 13]. Neither review contributed to direct population-specific claims.

### Eligibility concepts

Eligibility was assessed separately for direct population-specific evidence and for sources retained only for adjacent or contextual interpretation. A passing citation to Levesque et al. [4] was insufficient.

The following criteria defined direct population-specific empirical evidence:

1. publication in 2013 or later in English or German;
2. examination of access to or utilization of mental-health services, or of a clearly defined mental-health diagnostic or treatment pathway, for children or adolescents aged 0–17 years, pregnant or postpartum people, or adults aged 65 years or older;
3. access for the eligible target population as the explicit unit of analysis. Data could be obtained from members of the target population, caregivers or family members, health- or social-care professionals, or service organizations. For mixed-age service populations, findings had to be separately attributable to the eligible age group; otherwise, the source was classified as adjacent evidence;
4. explicit use of the Levesque framework beyond a passing citation to inform study design, data collection, measurement, analysis, coding, or interpretation; and
5. completed empirical findings.

Eligible completed evidence syntheses—including systematic reviews, scoping reviews, mapping reviews, rapid reviews, integrative reviews, and umbrella reviews or overviews of reviews—were classified as direct review-level evidence when they met the same publication-date, language, population, mental-health-access, and substantive framework-use criteria. Evidence syntheses spanning eligible and ineligible age ranges contributed to the direct population-specific synthesis only when findings for the prespecified age group were reported separately; otherwise, they were classified as adjacent review-level evidence.

Study protocols, conceptual articles, general-health-care studies, and sources not meeting all direct criteria could be retained only as clearly labelled protocol, adjacent, or contextual evidence and did not contribute to direct cross-population claims.

### Data extraction and synthesis

Data were charted in a standardized evidence matrix covering bibliographic details, study design, country and setting, population, mental-health service or access pathway, substantive use of the Levesque framework, access dimensions, barriers and facilitators, and key findings. Elicit generated draft extraction fields, which were treated as preliminary. An empirical comparison of Elicit and human extraction identified incomplete extraction of nuanced study information, supporting verification against the original reports [25]. JCC verified the retained extraction fields, and BR independently checked the extracted study data against the corresponding full-text publications. Bibliographic information was checked against publisher or DOI records.

Each source was assigned both (a) a source type - empirical primary study, review or evidence synthesis, study protocol, or conceptual paper – and (b) a role in the synthesis – direct population-specific, adjacent, or contextual. Only direct sources informed core cross-population claims. Findings were compared using four predefined questions: the access stages at which barriers and facilitators were reported, the principal actors or gatekeepers, the relevant service-system interfaces, and the population-specific conditions shaping acceptability and sustained engagement. The complete evidence matrix is provided in Supplementary Material S6.

### AI-assisted procedures and human oversight

Available task-level documentation of the AI-assisted workflow is provided in Supplementary Material S3. The supplement records the tools and purposes, available provider and version information, prompt history, outputs used in the review, human verification procedures, source and claim verification, data-protection considerations, and any historical metadata that could not be recovered. Available Elicit research questions, screening criteria, extraction instructions, and report documentation are provided in Supplementary Material S4.

Elicit supported semantic retrieval, relevance ranking, automated preliminary screening and draft extraction of study characteristics. The automatic preselection criteria covered framework use, data completeness, a mental-health focus and the target population. JCC developed the research questions and eligibility criteria, initiated searches, reviewed reports, and made final eligibility and evidence-classification decisions following human review. BR independently checked extracted study data against the full texts. JCC and BR independently conducted the methodological appraisal. Retained data, appraisal judgments, framework coding and interpretation were subject to author verification and decision-making.

ChatGPT, a generative-AI conversational tool (OpenAI; GPT-5.5 Pro on 6 May 2026 and GPT-5.6 Pro during the July–August 2026 revision) was used to support manuscript structuring, translation, linguistic refinement, preparation of transparency documentation, and organization of editor and reviewer comments. All suggested text and structural changes were evaluated, revised, or rejected by the authors. AI tools were not assigned responsibility for scientific decisions, source verification, or conclusions. During the September 2026 revision, ChatGPT also supported analysis of the appraisal workbooks, calculation and checking of agreement statistics, and drafting of the appraisal results and related discussion. Appraisal judgments and consensus decisions were made by JCC and BR.

### Analytical positioning

This review maps the available Levesque-informed evidence and generates comparative, framework-based propositions. It does not estimate the prevalence, effect size, or relative importance of access barriers. Cross-population statements were interpreted considering evidence directness, study type, and the volume of evidence available for each population. Contextual and adjacent sources supported interpretation but were not treated as direct population-specific evidence.

### Methodological quality appraisal and relevance classification

A formal methodological quality appraisal was conducted to support the interpretation of the mapped evidence. Across the 37-source evidence map, 23 empirical primary studies were appraised using the design-specific Mixed Methods Appraisal Tool (MMAT) 2018 [26] criteria, and 11 reviews or evidence syntheses were appraised using the JBI Critical Appraisal Checklist for Systematic Reviews and Research Syntheses [27]. One study protocol and two conceptual or framework-level sources were not formally appraised. Ten sources constituted the direct population-specific evidence base; appraisal of adjacent sources informed interpretation but did not allow them to contribute to direct cross-population claims. Appraisal informed interpretation but did not determine inclusion or evidence role. The protocol described planned research without completed empirical findings [28].

JCC and BR independently completed the design-specific MMAT 2018 criteria for empirical primary studies and the JBI checklist for review-level evidence. The three mixed-methods studies were appraised separately for their qualitative component, the appropriate quantitative component, and mixed-methods integration. The independently revised ratings saved before the consensus conference were used for agreement analysis. Incorrectly assigned JBI “not applicable” ratings were uniformly recoded to “no” for analysis and final reporting. Remaining disagreements were resolved by discussion and consultation of the original reports. Final item-level judgments and evidence roles are reported in Supplementary Table S1. No global MMAT score or overall high-, moderate-, or low-quality category was calculated.

Agreement was calculated at the source–criterion level as percentage agreement and unweighted Cohen’s κ, separately for the main MMAT ratings, the additional mixed-methods components, and JBI. A descriptive pooled κ combined these areas after harmonizing MMAT “can’t tell” and JBI “unclear” as an uncertainty category. This pooled statistic summarizes agreement across heterogeneous appraisal criteria and is not a measure of a single quality scale. A sensitivity calculation excluded the MMAT screening items. Consensus ratings were used to describe methodological findings, whereas agreement statistics were calculated from the preconference ratings.

### Protocol and registration

The review was preregistered on 6 February 2026 on the Open Science Framework (OSF; https://osf.io/8ntrb/) as a PRISMA-ScR-oriented scoping review. The Levesque framework was selected during initial planning, alongside the three target populations. Supplementary Material S2 describes protocol alignment and the implemented evidence-identification, selection, appraisal and synthesis procedures. The associated project is available at https://osf.io/fv2ap/.

### Reporting guidelines

Reporting of this scoping review was guided by PRISMA-ScR [23]. The checklist records the reporting of implemented procedures and remaining documentation gaps. The PRISMA-ScR checklist is provided as Supplementary Material S7. Reporting of generative-AI use was additionally assessed using the GAMER statement [29], and the GAMER checklist is provided as Supplementary Material S8.

## Results

The three original Elicit reports generated structured extraction outputs for 50 records: 31 from the perinatal report, 17 from the older-adult report, and two from the broader mental-health report. The dedicated youth-focused report returned 5,000 platform-ranked records, advanced 20 records to Elicit-supported full-text screening, and proposed three sources for extraction. Human verification retained Martínez-Jaime et al. [8] as direct empirical evidence and classified Werlen et al. [30] and Barbo and Petrucka [31] as adjacent review-level evidence. Of the ten records for which Elicit lacked full-text access, six were considered potentially relevant at title/abstract level and were subsequently retrieved and assessed; three were retained as adjacent evidence and three were excluded. The remaining four were excluded during human title/abstract screening and were not sought for retrieval.

Forward citation tracking in Web of Science identified 2,423 citing records. Population-specific filters yielded 185 youth-related, 48 perinatal, and 67 older-adult hits, corresponding to 300 route-specific hits before cross-filter deduplication. PubMed “Cited by” added no further candidate records. The targeted PubMed update retrieved two records, neither eligible as direct evidence. PMID 41,709,297 corresponds to Williams-Ridgway et al., retained as adjacent evidence because its mixed target age range of 10–25 years did not provide separate findings for ages 0–17. It was also identified through the youth Elicit and citation routes and was counted only once in the final map. PMID 41,878,391 was a physical-activity intervention protocol without a mental-health-service-access focus or substantive Levesque application and was excluded. The reported aggregate flow comprised 372 candidate-record entries (50 + 20 + 300 + 2) before deduplication, five duplicate removals, 367 unique records screened by title and abstract, and 297 exclusions at that stage. All 70 reports sought were retrieved; JCC assessed them in full text and excluded 33. The final evidence map retained 37 sources: 23 empirical primary studies, 11 reviews or evidence syntheses, one study protocol, and two conceptual or framework-level sources. Ten sources met all criteria for the direct population-specific synthesis. Supplementary Material S5 reports aggregate route counts and a partial retrospective account of candidate-level decisions; a complete historical screening log is unavailable.

**Fig. 1:**
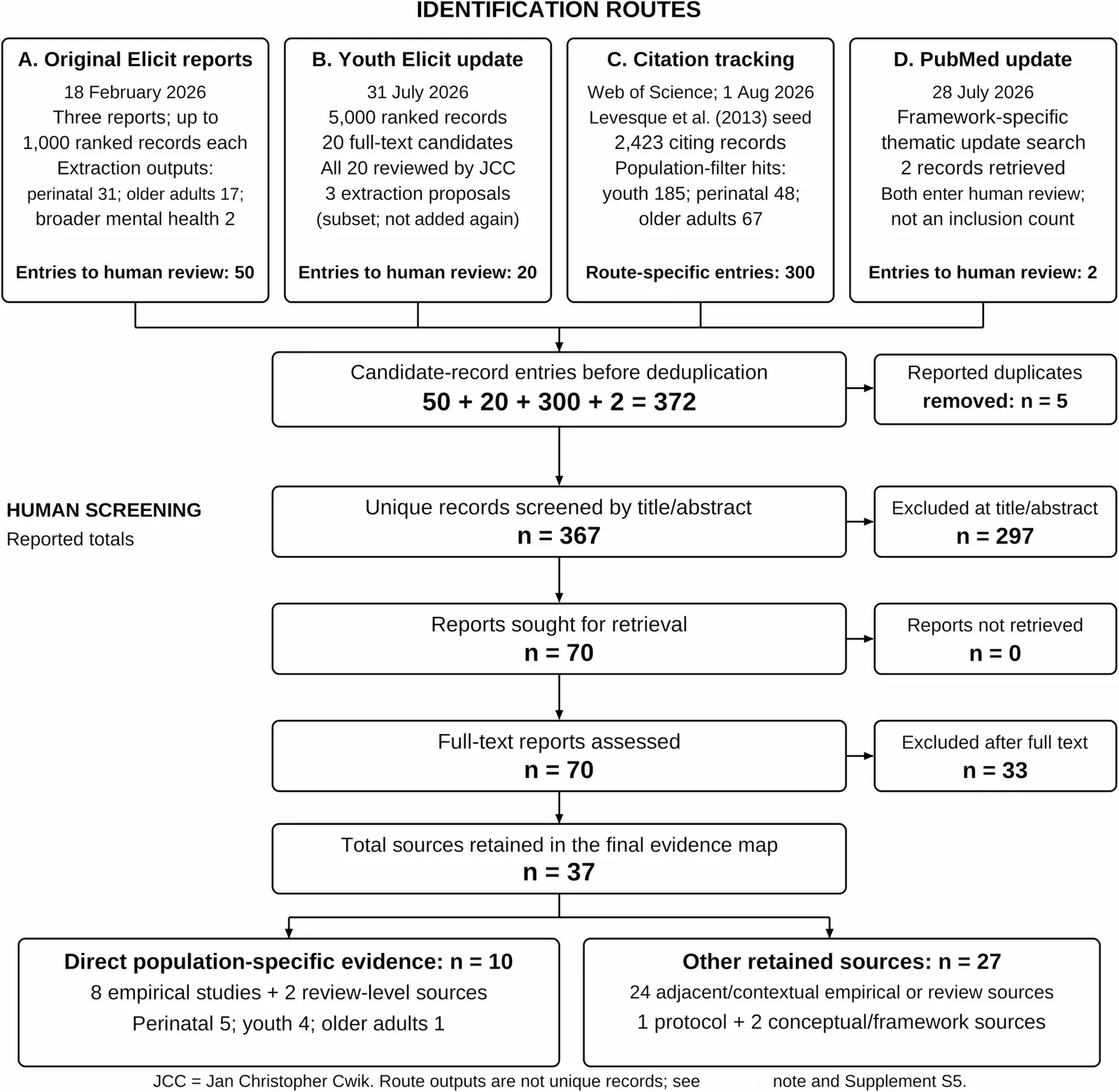
Identification routes, reported human screening, and final evidence roles. Note: Only the four route-entry counts are added (50 + 20 + 300 + 2 = 372). All 20 youth candidates entered human verification; the three extraction proposals are not an additional count. PubMed “Cited by” added no candidates, but its examined total was not recorded. Counts below deduplication are reported aggregates, not a complete record-level audit. Adjacent/contextual evidence is separate from the ten direct sources. Supplementary Material S5 documents route overlap and the available decisions

### Overview of the evidence base

The updated direct evidence base comprised ten sources. Perinatal mental-health access was represented by three empirical studies and two review-level sources; youth access by four empirical studies; and older-adult access by one empirical study. The fourth youth-focused Elicit report identified one already included direct study and two adjacent review-level applications. Human follow-up of records unavailable to Elicit retained three adjacent youth sources, one of which had also been retrieved by the PubMed update. The updated youth evidence therefore extended beyond ADHD diagnostic delay to include racial and ethnic disparities, primary-care consultation models, service availability and directory accuracy, intervention strategies, and access barriers affecting refugee and asylum-seeking youth. Direct and adjacent sources were analyzed separately throughout (see Table 1).

**Table 1:** Direct, adjacent, and contextual Levesque-informed sources included in the narrative synthesis

Population / domain	Source	Design, setting, and sample (where available)	Role in synthesis	Levesque framework use	Main access contribution
Perinatal	**Ash et al. 2026** [5]	Empirical primary study; qualitative focus groups; Black perinatal women; southeastern United States; *n* = 43	Direct empirical evidence	Conceptual and analytical framework	Medical mistrust, stigma, transparency of screening, and culturally responsive trauma-informed care emerged as cross-cutting access issues.
Perinatal	**Salameh et al. 2025** [6]	Empirical primary study; descriptive qualitative telephone interviews; Syrian refugee women with postpartum depression symptoms; Türkiye; *n* = 15	Direct empirical evidence	Supply-side dimensions used in thematic analysis	Knowledge gaps, stigma, language, cultural fit, transport, childcare, time constraints, and screening gaps restricted access.
Perinatal	**Viveiros & Darling 2018** [32]	Empirical primary study; qualitative study; women receiving continuity-of-care midwifery; *n* = 16	Direct empirical evidence	Levesque-informed analysis of barriers and facilitators	Five themes concerned cultural values, knowledge, relationships, flexibility, and system gaps. Barriers included stigma and fear, broken referral pathways, distance, limited specialist capacity, baby-centredness, six-week discharge, and cost; continuity, community, and advocacy facilitated access.
Perinatal	**Viveiros & Darling 2019** [16]	Scoping review; 26 publications; midwifery and perinatal mental health	Direct review-level evidence	Barriers mapped onto all ten dimensions	Early pathway barriers were common, especially approachability and the ability to perceive need.
Perinatal	**Marshman et al. 2023 **[33]	Rapid review; 31 articles included from 539 screened references	Direct review-level evidence	Barriers mapped to Levesque supply-side dimensions	Stigma operated at societal, institutional, and individual levels. Obstetricians, midwives, and patients experienced overlapping barriers to detection, discussion, referral, and connection to support.
Perinatal	**Berger et al. 2020** [28]	Study protocol; qualitative; Switzerland; planned *n* = 24 women with a prior perinatal mental disorder plus three interprofessional focus groups of 5–8 professionals	Contextual protocol; planned application	Interview guide and analysis informed by all ten dimensions	The planned study addresses normalization of symptoms, stigma, service fragmentation, and the need for improved care coordination.
Perinatal	**de Jong-Bardelmeijer et al. 2026** [34]	Empirical primary study; cross-sectional questionnaire; women aged ≥ 18 years, 12–30 months postpartum; Netherlands; *n* = 1,268 responses	Adjacent empirical evidence; general postpartum health-care utilization	Determinants derived from Levesque and the Andersen model	Unawareness of available postpartum health-care services was associated with lower utilization. The study illustrates quantitative use of Andersen-derived determinants partly aligned with Levesque demand-side access dimensions, without a validated Levesque scale.
Older adults	**Schwarz et al. 2022** [9]	Empirical primary study; qualitative interviews; Austria; three chronic-condition cases. Older-adult mental-illness case: *n* = 10 interviewees (two service users, two carers, five providers, one researcher); target population > 65 years, excluding dementia.	Direct empirical evidence	A priori use of five dimensions and abilities	Low or distorted perception of need, stigma, limited social-psychiatric services, poor coordination across health and social care, financial burden, and the need for continuity and trust shaped access.
Older adults	**Kurpas et al. 2018** [19]	Empirical primary study; qualitative framework analysis; Polish frail-older-adult care stakeholders; five focus groups; *n* = 44	Adjacent empirical evidence; general frail-older-adult care interface	A posteriori use of all dimensions and abilities	Psychological-support gaps, staff shortages, waiting times, remote clinics, costs, and the need for health-social care integration were identified.
Older adults	**Dableh et al. 2025** [18]	Empirical primary study; qualitative inquiry; Lebanon; adults aged 60–92 years, informal caregivers, and service providers; *n* = 57	Adjacent empirical evidence; primary health care	A priori use of all dimensions and abilities	Primary-care access barriers included information, transport, mobility, finances, provider attitudes, cognitive and sensory limitations, and continuity.
Older adults	**Dableh et al. 2025** [17]	Empirical primary study; co-design; Lebanon; older people, family members, and primary health-care providers; *n* = 13 plus three individual advisor interviews	Adjacent implementation evidence	Levesque-aligned dimensions; explicit use unclear in the report	The study developed prototypes for depression and cognitive screening in primary-care centers and an awareness campaign.
Dementia / older adults	**Bartlett et al. 2025** [35]	Empirical primary study; mixed-methods analysis; England; survey of 32 social-farm managers, four online focus groups, and 14 single or dyad interviews with people living with dementia and supporters	Adjacent/contextual empirical evidence; dementia/social-care setting	A priori navigational guide	Travel and carer dependence affected access; the analysis emphasized intersectionality and access within services.
Youth mental health	**Martínez-Jaime et al. 2024** [8]	Empirical primary study; retrospective pathway study; Mexico City tertiary psychiatric hospital; *n* = 177 child/adolescent–caregiver dyads with diagnosed ADHD; pathway ages reported by caregivers.	Direct empirical evidence	Primary theoretical framework; survey and access pathway	Demand-side abilities were operationalized as diagnostic milestones. Diagnostic delay and observed sex differences concerned already diagnosed hospital attendees; people who never reached diagnosis were not represented.
Youth mental health	**Daskalska et al. 2025** [36]	Empirical primary study; qualitative interviews concerning access for Black and Latino adolescents with anxiety or depression; *n* = 10 interviewees (one adolescent, three parents, six professionals).	Direct empirical evidence	Levesque-informed interpretive framework	Six themes addressed first points of contact, provider preferences, culturally appropriate, trauma-informed and family-centered care, community awareness, and service availability.
Youth mental health	**Daskalska et al. 2024** [37]	Empirical primary study; mixed-methods evaluation of the Wisconsin Child Psychiatry Consultation Program; anonymous, unlinked provider surveys at baseline (*n* = 342) and follow-up (*n* = 114), with free-text responses.	Direct empirical evidence	Provider-reported access barriers and program findings interpreted using an adapted Levesque framework	Follow-up respondents reported higher confidence and skill in managing childhood anxiety and depression. Referral, waiting-time, and insurance barriers persisted; the evaluation did not establish improved patient access.
Youth mental health	**Adams 2024** [38]	Empirical primary study; quantitative descriptive survey; 117 community mental health centers and 117 federally qualified health centers	Direct empirical evidence	Patient-Centered Access to Care Framework informed study design and access domains	Approximately 10% of centers were closed and 20% did not provide outpatient mental-health care. Community mental health centers had more clinicians but longer waits, and online service directories were often inaccurate or outdated.
Youth mental health	**Werlen et al. 2020** [30]	Systematic review and meta-analysis; 34 randomized controlled trials	Adjacent review-level evidence	Interventions and outcomes mapped to Levesque dimensions	School-based interventions often improved knowledge and attitudes but less often actual utilization; appointment reminders and motivational strategies improved first-appointment attendance. Classified as adjacent because eligibility extended to participants under 19 years or mixed samples.
Youth refugees	**Barbo & Petrucka 2024** [31]	Integrative review; 29 sources; youth refugees and asylum seekers	Adjacent review-level evidence	Deductive mapping to all five dimension-ability pairs	Barriers included low mental-health literacy, stigma, cultural mismatch, language, service rules and age cut-offs, waits, costs, and insufficient provider competence. Classified as adjacent because included samples extended beyond 0–17 years.
Refugee minors	**Fathi Afshar 2019** [39]	Empirical primary study; qualitative master’s thesis; refugee children, unaccompanied minor aliens, and health-care providers	Adjacent empirical evidence	Levesque-informed provider interviews	The study highlighted health literacy, communication, care coordination, and timely access, but focused on general health care rather than mental-health access alone.
Youth / young adults	**Williams-Ridgway et al. 2026** [40]	Empirical primary study; qualitative interviews; 12 health-care professionals; target service-user group aged 10–25 years	Adjacent empirical evidence	Levesque bidirectional access framework informed the interview schedule and interpretation	Gatekeeping, service invisibility, inaccessible locations, language and cultural mismatch, workforce diversity, training, awareness, shame, and stigma shaped access to eating-disorder services.
Forcibly displaced youth	**Yılmaz et al. 2025** [41]	Overview of reviews; 42 reviews; approximate age range 10–24 years	Adjacent review-level evidence	Levesque-guided five-stage narrative synthesis	The synthesis identified low mental-health literacy, stigma, limited autonomy, legal insecurity, unstable housing, weak support, service fragmentation, and a lack of culturally and linguistically responsive care.
Adolescent / young-adult health	**Harper et al. 2018** [7]	Conceptual analysis/commentary; United States; no empirical sample	Contextual conceptual evidence	Supplementary framework alongside a social-ecological model	Confidentiality, youth-friendly services, schools, parental roles, and declining care contact in young adulthood were emphasized.

Note: Table 1 presents all ten direct sources and selected adjacent or contextual sources discussed in the main narrative synthesis. The complete 37-source evidence matrix is provided in Supplementary Material S6. “Direct” denotes population and topic relevance, not necessarily first-person reporting

The mapped studies described barriers involving recognition, interpretation, and navigation before formal service contact. These accounts do not establish a common temporal sequence or a ranking of barriers across populations. This pattern reinforces the value of pathway-oriented access frameworks in mental health research, where delayed recognition of need, stigma, uncertainty, and fragmented referral structures may prevent access long before formal treatment contact occurs.

The framework was generally used to classify barriers or structure qualitative analysis; validated measurement was uncommon. One exception was the ADHD diagnosis study, which operationalized demand-side abilities as temporal milestones along the diagnostic pathway [8]. Another quantitative perinatal study incorporated Levesque-derived determinants into an analysis of postpartum health care utilization [32]. Validated Levesque-specific measurement remained limited in the retained perinatal evidence.

Table 2 summarizes how the ten Levesque dimensions were represented in the mapped mental-health access evidence. Only a minority of direct or closely aligned sources explicitly attempted to represent the complete model, either by mapping all ten dimensions or by considering both service dimensions and population abilities. Several sources instead focused on selected domains, for example supply-side dimensions in perinatal refugee care or demand-side milestones in the ADHD diagnostic pathway. The table organizes findings across the evidence map; individual studies did not necessarily measure all ten dimensions.

**Table 2:** Integrated synthesis of Levesque dimension-ability pairs across selected life-course stages

Levesque dimension-ability pair	Pathway-oriented question	Integrated findings from direct population-specific evidence	Adjacent/contextual extensions and remaining framework-use gaps
**Approachability /** **ability to perceive**	Can people recognize a need for care and identify relevant, trustworthy services and entry points?	**Perinatal**: Symptoms were often normalized or poorly recognized, and services or pathways were not readily visible. Transparent screening, proactive information, continuity with midwives, and knowledgeable professionals supported recognition and disclosure [5, 6, 16, 32, 33]. **Youth**: ADHD recognition depended strongly on caregivers and teachers; Black and Latino teens, parents, and professionals described limited community awareness and uncertainty about first points of contact. Public directories could be inaccurate or outdated, and listed centers could be closed or not provide outpatient mental-health care [8, 36, 38]. **Older adults**: Low or distorted perception of need and limited visibility of social-psychiatric services delayed access [9]. **Integrated interpretation**: Across the three life-course groups, studies described barriers before formal service contact; their relative frequency or importance was not estimated. Service existence alone did not ensure that need, service relevance, or an appropriate entry point could be recognized.	**Adjacent/contextual extension**: Adjacent perinatal and older-adult studies likewise linked service unawareness, information barriers, and limited health literacy to lower use or delayed navigation [18, 34]. Refugee and minoritized-youth sources added unfamiliarity with health systems, low mental-health literacy, and service invisibility; intervention evidence showed that knowledge and attitudes improved more consistently than actual utilization [30, 31, 39–41]. **Remaining gap**: Few studies jointly measured service approachability and the ability to perceive need using validated or explicitly linked indicators.
**Acceptability /** **ability to seek**	Are services socially, culturally, and personally acceptable, and can people decide to seek help and disclose need safely?	**Perinatal**: Stigma, fear of judgment or consequences, medical mistrust, language and cultural mismatch, and uncertainty about screening inhibited disclosure and help-seeking. Relational continuity, advocacy, and culturally responsive, trauma-informed care facilitated safer disclosure [5, 6, 32, 33]. **Youth**: Provider preferences, cultural responsiveness, trauma-informed and family-centered care, community awareness, and the roles of parents, teachers, and primary-care professionals shaped willingness and opportunities to seek care [8, 36, 37]. **Older adults**: Stigma and trust in providers and services affected willingness to seek care and sustain contact [9]. **Integrated interpretation**: Similarly labelled barriers had life-stage-specific meanings: perinatal fear concerned disclosure and possible consequences for motherhood; youth help-seeking was strongly mediated by family, school, and confidentiality conditions; and later-life access depended on trust and whether mental-health needs were considered legitimate and treatable.	**Adjacent/contextual extension**: Refugee, forcibly displaced, and minoritized-youth evidence highlighted shame, confidentiality concerns, legal insecurity, limited autonomy, cultural and linguistic mismatch, and the need for cultural humility and workforce diversity [7, 31, 40, 41]. Older-adult adjacent evidence added provider attitudes and communication barriers [18]. **Remaining gap**: Acceptability and ability to seek were commonly discussed together rather than operationalized as separate but interacting service- and population-side constructs.
**Availability and accommodation /** **ability to reach**	Are staff, appointments, referral routes, and locations available in practice, and can people physically, digitally, and organizationally reach them?	**Perinatal**: Broken referral pathways, limited specialist capacity, distance, transport, childcare and time constraints, early discharge from midwifery care, and insufficient professional training restricted access [6, 32, 33]. **Youth**: Referral knowledge and gatekeeping, long waits, insurance restrictions, closed or non-providing centers, and inaccurate directories made nominal availability differ from usable access. Follow-up providers reported higher confidence and skill, while system-level waits and coverage constraints persisted [8, 37, 38]. **Older adults**: Limited social-psychiatric services and poor coordination across health and social care restricted reach, transfer, and continuity [9]. **Integrated interpretation**: Gatekeepers and service interfaces differed by stage - maternity and primary-care services in the perinatal period, caregivers, schools, and primary care in youth, and health-social care interfaces in later life - but bottlenecks repeatedly accumulated at referral and coordination points.	**Adjacent/contextual extension**: Older-adult sources added staff shortages, waiting times, remote clinics, transport and mobility limitations, carer dependence, and weak health-social care integration [18, 19, 35]. Youth sources added GP gatekeeping, inaccessible locations, restrictive rules or age cut-offs, fragmented systems, unstable housing, and legal constraints [31, 39–41]. **Remaining gap**: Objective capacity, directory accuracy, actual appointment availability, and the population ability to reach services were rarely examined within the same study.
**Affordability /** **ability to pay**	Can care be obtained without prohibitive direct or indirect costs, and do people have the resources and coverage needed to use it?	**Perinatal**: Direct costs and indirect burdens such as transport, childcare, and time could impede access, particularly where specialist services were distant or fragmented [6,16 , 32]. **Youth**: Insurance coverage remained a barrier in the consultation-program context, while safety-net accessibility depended on insurance acceptance and the availability of free or sliding-scale options [37, 38]. **Older adults**: Financial burden constrained access within the older-adult mental-illness case study [9]. **Integrated interpretation**: Affordability interacted with service availability and practical reach. Removing direct fees alone could not ensure timely or appropriate care when capacity, referral, transport, or cultural-fit barriers remained.	**Adjacent/contextual extension**: Adjacent older-adult evidence described costs and financial constraints within frail-older-adult and primary-care pathways [18, 19]. Refugee-youth evidence emphasized competing resettlement needs, legal entitlements, and broader economic precarity [31, 39, 41]. **Remaining gap**: Affordability and ability to pay were among the least consistently operationalized pairs and were usually described qualitatively rather than assessed with validated or longitudinal measures.
**Appropriateness /** **ability to engage**	Is care clinically, culturally, developmentally, and contextually suitable, and can people participate, continue, and benefit?	**Perinatal**: Appropriateness depended on continuity, informed choice, advocacy, direct referral capacity, specialist competence, and culturally responsive, trauma-informed care. Stigma at patient, professional, and institutional levels could disrupt discussion, referral, and sustained engagement [5, 6, 32, 33]. **Youth**: Young people and families valued culturally appropriate, trauma-informed, family-centered care and appropriate provider characteristics. Follow-up providers reported higher confidence and skill, but engagement remained constrained by waits and insurance; the diagnosed ADHD sample showed sex differences in caregiver-reported pathway timing [8, 36, 37]. **Older adults**: Trust, continuity, coordination, and care tailored to complex mental-health and social-care needs supported engagement [9]. **Integrated interpretation**: Across stages, sustained engagement depended on relational fit, continuity, and coordination rather than on service entry alone.	**Adjacent/contextual extension**: Intervention evidence suggested that appointment reminders and motivational strategies can improve first-appointment attendance, whereas knowledge-focused programs less consistently changed utilization [30]. Refugee and minoritized-youth evidence emphasized low-threshold, culturally and linguistically responsive, family-inclusive services and more diverse workforces [31, 40, 41]. Older-adult adjacent evidence added cognitive and sensory accommodation, intersectionality, carer support, and within-service access [18, 35]. **Remaining gap**: Few studies followed engagement longitudinally, tested interventions beyond initial attendance, or linked appropriateness and ability to engage using validated paired measures.

The broader health care access literature shows a similar pattern. A scoping review of 31 empirical applications of the Levesque framework found that the framework was more often used a posteriori to organize or analyze data than a priori to develop tools, and that only 11 studies examined both health-system dimensions and corresponding population abilities [12]. A UK adult mental health mapping review showed a parallel measurement imbalance: among 152 studies, access was most often measured as health care utilization or health care seeking, whereas perception of need, desire for care, and health care consequences were rarely addressed [13]. Only 17 studies discussed applying or developing a theoretical framework, underscoring limited theory-driven operationalization in mental health access research [13].

### Applications by population group

#### Perinatal mental health

Five sources met the direct perinatal criteria: three empirical primary studies and two review-level sources. Ash et al. [5] identified medical mistrust, stigma, concerns about screening transparency, and the need for culturally responsive and trauma-informed care among Black perinatal women. Salameh et al. [6] identified knowledge gaps, stigma, language and cultural mismatch, transport, childcare, time constraints, and screening gaps among Syrian refugee women with symptoms of postpartum depression. The qualitative study by Viveiros and Darling [33] showed how stigma and fear, broken referral pathways, distant service locations, limited specialist capacity, baby-centered service models, discharge from midwifery care at six weeks postpartum, and cost could restrict access. Continuity, community connection, advocacy, informed choice, and knowledgeable midwives facilitated disclosure and navigation. Their subsequent scoping review [16] mapped barriers across all ten Levesque dimensions and showed that barriers at the stages of approachability and ability to perceive need were particularly recurrent. The rapid review by Marshman et al. [34], which included 31 articles, extended these findings across patient, midwife, and obstetrician perspectives. It mapped barriers to Levesque’s supply-side dimensions and showed that stigma operated at societal, institutional, and individual levels, affecting detection, discussion of mental-health concerns, referral, and connection to available supports. Together, the direct perinatal evidence suggests that access depends not only on symptom recognition and nominal service availability but also on relational continuity, referral authority, specialist capacity, and whether women can disclose concerns without fear of judgment or adverse consequences.

Adjacent and contextual perinatal evidence reinforced, but was not used to establish, these direct population-specific claims. Unawareness of available postpartum services was associated with lower general health-care utilization [32]. Broader reviews and provider or workforce studies identified recurrent barriers, limited specialist availability, inadequate professional training, and inconsistent screening and referral procedures [10, 11, 15, 35]. Implementation and intervention studies highlighted transparent screening linked to referral pathways, integrated and relationship-centered care, digital options, and affordability mechanisms as potential facilitators [14, 36–39]. A Levesque-informed meta-synthesis of maternity-care access among African-born women in Australia further illustrated how culturally unsafe communication, discrimination, limited information, mistrust, navigation difficulties, waiting times, and discontinuity may shape access during pregnancy and the postnatal period [40]. Because this synthesis concerned maternity-care rather than mental-health care specifically, it was used only for contextual interpretation.

#### Older-adult mental health

One source met all criteria for direct older-adult evidence. Schwarz et al. [9] examined access for independently living adults aged over 65 years with pre-existing mental illness, drawing mainly on stakeholder interviews alongside two service-user interviews, and identified low or distorted perception of need, stigma, limited social-psychiatric services, poor coordination across health and social care, financial burden, and the importance of continuity and trust.

Adjacent older-adult evidence addressed broader primary-care, frailty, dementia, social-care, and inequality contexts [17–19, 41–43]. These sources added information barriers, staff shortages, waiting times, transport and mobility constraints, provider attitudes, cognitive and sensory limitations, carer dependence, financial inequality, and weak health–social-care integration. Because these studies did not all concern population-specific access to mental-health services, they were used only to extend interpretation and did not contribute to direct cross-population claims.

#### Children and adolescents

Four empirical studies met the direct youth criteria. Martínez-Jaime et al. [8] operationalized the ADHD diagnostic pathway and described diagnostic delay, observed sex differences, and teachers’ role in symptom recognition among already diagnosed hospital attendees, using caregiver-reported pathway ages. Daskalska et al. [44] identified six themes shaping access for Black and Latino teens with anxiety or depression: the pandemic context, first points of contact, preferred professional characteristics, culturally appropriate trauma-informed and family-centered care, community awareness, and service availability. A second study by Daskalska et al. [45] evaluated the Wisconsin Child Psychiatry Consultation Program and found higher self-reported primary-care provider confidence and skill in the follow-up sample, while persistent barriers included limited referral knowledge, long waiting times, and insurance gaps. Adams’ [46] survey of safety-net health centers showed that formal service listings did not necessarily represent usable access: some centers were closed or did not offer outpatient mental-health care, centers with more clinicians could still have longer waits, and online directories were often inaccurate or outdated.

Adjacent youth evidence from the fourth Elicit workflow broadened the interpretation without contributing to the direct 0–17 count. Werlen et al. [30] showed that school-based interventions frequently improved knowledge and attitudes, whereas improvements in actual service utilization were less common; appointment reminders and motivational strategies were more effective for first-appointment attendance. Barbo and Petrucka [31] mapped compounding barriers for refugee and asylum-seeking youth across all five Levesque dimension–ability pairs, including low mental-health literacy, stigma, cultural and linguistic mismatch, restrictive service rules, waits, financial strain, and limited provider competence. Human follow-up of records unavailable to Elicit retained adjacent evidence on general health-care navigation for refugee minors, ethnic inequalities in eating-disorder services, and forced-displacement-related barriers [47–49].

### Contextual framework and adult mental-health evidence

A systematic mapping review of inequalities in access to UK adult NHS mental health services provides the closest mental-health-specific cross-cutting evidence, and is used as contextual evidence in the present synthesis. Synthesizing 152 primary studies, the review used the Levesque framework to classify access measures and barriers and showed that evidence was concentrated on utilization, referral, and service contact [13]. The perception of need and the consequences of access were rarely measured. Frequently reported barriers included stigma and discrimination, limited-service appropriateness, availability constraints, lack of trust, and difficulties recognizing symptoms, with the most common barriers aligning with the ability to reach and the ability to seek. These findings support the manuscript’s methodological argument about access measurement, but they should not be read as direct evidence for perinatal women, children/adolescents, or older adults (see Table 1).

### Methodological quality appraisal and evidence classification

Methodological appraisal covered 34 of the 37 sources: 23 empirical primary studies (13 qualitative, five quantitative non-randomized, two quantitative descriptive, and three mixed-methods studies) and 11 reviews or evidence syntheses. One study protocol and two conceptual or framework-level sources were not formally appraised. The ten direct population-specific sources formed a subset of the appraisal set. Appraisal informed interpretation but did not determine inclusion or direct, adjacent, or contextual classification.

Before consensus, agreement was 95.2% across 312 source–criterion pairs (297/312; descriptive pooled κ = 0.895). Agreement was 94.4% for the main MMAT ratings including screening (152/161; κ = 0.853), 93.3% for additional mixed-methods component ratings (28/30; κ = 0.870), and 96.7% for JBI (117/121; κ = 0.937). Excluding the 46 MMAT screening pairs yielded 94.4% agreement (251/266; pooled κ = 0.890). The 15 remaining differences were resolved by consensus (Table 3).

Final MMAT judgments supported the appropriateness of the qualitative approach, derivation of findings, and substantiation of interpretations in all 13 qualitative studies. Adequacy of data collection was uncertain in four studies and coherence across study components in three. Among the five quantitative non-randomized studies, participant representativeness and control of confounding were each rated “no” in four studies and “can’t tell” in one. Low nonresponse bias could not be established in either quantitative descriptive study. None of the three mixed-methods studies met the criterion requiring the constituent components to satisfy their respective quality criteria, although the adequacy of integration differed across studies.

Among the 11 reviews, recurrent negative judgments concerned breadth of information sources (7/11), appropriateness of critical appraisal criteria (6/11, with one further unclear judgment), and consideration of publication bias (8/11, with one further unclear judgment). These findings were considered in relation to each review’s purpose and design. They support cautious interpretation of the mapped patterns and limit population-level or causal conclusions. Supplementary Table S1 provides all final item-level judgments and relevance classifications.

**Table 3:** Methodological appraisal and agreement before consensus

Appraisal area	*n*	Agreement *n*/*N* (%)	Cohen’s κ	Remaining differences
MMAT main criteria including screening	23	152/161 (94.4)	0.853	9
Additional mixed-methods components	3¹	28/30 (93.3)	0.870	2
JBI review checklist	11	117/121 (96.7)	0.937	4
All appraisal areas	34	297/312 (95.2)	0.895²	15

Note: n = number of sources. Ten appraised sources were direct population-specific evidence (eight empirical studies and two reviews). One protocol and two conceptual/framework sources were not appraised. ¹ These three studies are included among the 23 MMAT studies; component ratings are additional criteria, not additional sources. ² Descriptive pooled unweighted κ with CT and Unclear harmonized. All JBI NA ratings were recoded to N. Excluding screening: 251/266 agreements (94.4%), κ = 0.890

### Thematic synthesis across Levesque dimensions

#### Approachability and ability to perceive: barriers before formal service contact

Across the selected populations, studies reported barriers before contact with formal services. These included difficulties recognizing symptoms as warranting care, uncertainty about which services were available or appropriate, and stigma or mistrust that discouraged disclosure and help-seeking. Services could therefore be formally available while remaining difficult to identify, interpret, or approach. This descriptive pattern does not establish that these barriers occur first, or are most important, in every access pathway.

Within the direct perinatal evidence, distress was often normalized as part of pregnancy, childbirth, or motherhood, and women lacked clear information about when symptoms warranted care or how to enter appropriate care pathways [6, 16, 33, 34]. Adjacent perinatal evidence similarly indicated that unawareness of available services and broader information gaps were associated with lower utilization or delayed navigation [11, 32]. In the direct older-adult study, low or distorted perception of need and limited visibility of social-psychiatric services delayed access [9]; adjacent older-adult studies added information barriers, limited preventive orientation, and difficulties recognizing cognitive needs [17, 18, 42].

The updated direct youth evidence identified three related forms of service invisibility: limited community awareness of mental-health resources, inaccurate or outdated service directories, and failure of first-contact professionals to identify or route need appropriately [44–46]. Adjacent refugee and minoritized-youth sources further showed that unfamiliarity with host-country health systems, limited mental-health literacy, and culturally shaped understandings or expressions of distress could make formally available services difficult to recognize as relevant [31, 47–49]. In ADHD pathways, caregivers often did not recognize core symptoms as indicating a mental-health problem, while teachers served as important facilitators of recognition [8]. Taken together, the evidence suggests that service availability alone does not ensure that need, service relevance, or an appropriate entry point can be recognized.

#### Acceptability and ability to seek: stigma, mistrust, and cultural fit

Acceptability barriers were prominent across all three life-course groups. Perinatal women faced stigma, fear of being judged as a bad mother, concerns about child protection involvement, discomfort with pharmacotherapy during pregnancy or breastfeeding, and cultural or language barriers [6, 10, 11, 38]. Black perinatal women experienced medical mistrust that cut across approachability, acceptability, and engagement [5]. Syrian refugee women faced mental illness stigma, refugee-related vulnerability, language barriers, and cultural preferences in provider choice [6]. Older adults faced stigma around mental illness, fear of treatment, cultural norms, and negative expectations about providers [9, 18, 42]. For adolescents and young adults, confidentiality and youth-friendly care were central acceptability issues [7]. In the adolescent and young-adult conceptual evidence, acceptability concerned whether services protected privacy while supporting age-appropriate autonomy. Confidentiality concerns may discourage disclosure or help-seeking when young people expect sensitive information to be shared with parents, schools, or insurers, or when the limits of confidentiality are unclear. Youth-friendly care therefore includes clear explanations of confidentiality and its limits, opportunities to speak with clinicians without caregivers present where appropriate, nonjudgmental and developmentally appropriate communication, flexible access arrangements, and calibrated parental involvement. The updated direct studies provide empirical evidence on professional characteristics, racial/ethnic representation, trauma-informed and family-centered care, stigma, trust, and school- or family-mediated help-seeking. Adjacent evidence adds culturally specific shame, confidentiality concerns, language barriers, and fear of discrimination among refugee and minoritized ethnic youth.

#### Availability, accommodation, and ability to reach: services exist, but pathways fail

Availability barriers were frequently structural. In perinatal care, studies reported specialist workforce shortages, long waiting lists, fragmented referrals, inconsistent screening, limited provider training, and lack of mandated screening [10, 15, 16, 35]. The older-adult report identified shortages of social psychiatric services, high staff turnover, remote clinics, long waiting times, transport problems, mobility restrictions, and poor coordination across health and social care [9, 18, 19]. In youth ADHD care, the distance to specialized services and long wait times contributed to delayed diagnosis [8]. Service availability cannot be inferred from staffing or directory presence alone. Adams [46] found closures, non-provision of child outpatient services, longer waits in better-staffed centers, and inaccurate directories. Daskalska et al. [45] reported higher provider confidence at follow-up alongside persistent waits, referral gaps, and insurance restrictions; the design did not establish a causal program effect. In perinatal care, broken referrals, distant services, limited capacity, and time-limited midwifery follow-up interrupted progression [33].

#### Affordability and ability to pay: necessary but insufficient

Financial barriers recurred, but affordability alone was insufficient. Perinatal women faced direct and indirect costs, including transport, childcare, time, and the consequences of limited referral options, while policy-level changes in insurance coverage affected psychotherapy utilization without necessarily ensuring adequate treatment intensity [6, 33, 39]. Older-adult evidence described treatment costs, financial precarity, economic crises, and inequitable access to community and specialist services [18, 19, 42, 43]. Insurance restrictions and the acceptance of insurance or sliding-scale arrangements emerged as direct youth-system barriers [45, 46]. Adjacent refugee-youth evidence further showed that care costs competed with housing, food, transport, employment, and resettlement needs [31, 47, 49]. Financial protection therefore needs to be combined with service availability, culturally appropriate pathways, and continuity mechanisms; otherwise, utilization may increase without improving engagement or the adequacy of care.

#### Appropriateness and ability to engage: the endpoint is meaningful care, not contact

Appropriateness emerged as a central endpoint of access. Perinatal studies called for perinatal-specific competence, trauma-informed care, culturally responsive services, transparent screening, and reliable referral pathways [5, 10, 11, 14, 36]. Older-adult studies emphasized continuity, coordination, care navigators, primary care integration, social prescribing, services suitable for complex needs, and accommodations for cognitive or sensory limitations [9, 17–19, 41]. In dementia-related access, Bartlett et al. argued that access must include not only entry into a service but access to meaningful activities within the service, and that intersectionality should be incorporated into Levesque-based analysis [41]. Youth studies similarly suggest that school-based detection and developmentally appropriate, confidentiality-sensitive services are necessary for sustained engagement [7, 8]. Overall, appropriate access requires both provider capability and service fit. Consultation-program participants reported higher confidence and skill in the follow-up sample, but the unlinked, uncontrolled comparison does not establish a program effect or improved patient access [45]. Direct qualitative youth evidence emphasized culturally responsive, trauma-informed, and family-centered care [44], while adjacent qualitative evidence highlighted workforce diversity and cultural-humility training [48]. Adjacent intervention evidence suggested that appointment reminders and motivational strategies could improve first-appointment attendance [30]. In the direct perinatal evidence, continuity, advocacy, and informed choice supported disclosure and navigation [33], while referral capacity and connection to available supports were also central [34].

#### Methodological gaps

Four methodological gaps stand out (see Table 4). First, the framework is more often used as a descriptive coding structure than as a basis for measurement. Second, coverage of service dimensions and population abilities is uneven; the ADHD access study, for example, measured demand-side temporal milestones without detailed service characteristics [8]. Third, few studies test how dimensions interact. For example, stigma may reduce help-seeking, but its effect may depend on service cultural fit, confidentiality, affordability, and referral design. Fourth, intervention studies rarely evaluate access outcomes across all stages, making it difficult to know whether proposed facilitators improve approachability, utilization, or sustained engagement.

A conceptually distinct gap concerned the fidelity and completeness with which the Levesque framework was used. Studies varied in whether the framework was applied a priori to shape research questions, data collection, or measurement, or a posteriori to organize and interpret findings; some cited it only as a supplementary conceptual reference. Few studies reported explicit coding rules, addressed overlap between dimensions, linked corresponding service dimensions and population abilities, or examined all ten components. Future applications should report when and how the framework entered the research process, whether it was modified, how each dimension was operationally defined, and whether the analysis was deductive, inductive, or combined.

These gaps are consistent with contextual evidence from both broader health care access and adult mental-health research. Empirical applications of the Levesque framework have often represented framework components unevenly, focusing on either service dimensions or population abilities, and some access phenomena have proved difficult to assign to a single category [12]. A similar pattern appears in adult mental health services, where access is often operationalized as utilization despite evidence that stigma, mistrust, service availability, appropriateness, and recognition of need shape earlier and later access stages [13]. This is especially important in mental health care because barriers such as stigma, mistrust, transport, waiting time, cost, and continuity often interact across several dimensions.

**Table 4:** Manuscript-level research agenda

Gap	Why it matters	Suggested next study design
Validated measurement	Most studies classify barriers or quantify utilization but do not measure Levesque dimensions with validated scales; broader applications also often represent the framework only partially (12), and adult UK mental health research is dominated by utilization rather than need, perception, or consequences (13).	Develop and validate population-sensitive Levesque instruments for mental health access, including perinatal, older-adult, and youth modules that distinguish potential access from realized access.
Supply-side operationalization	Approachability, acceptability, availability, affordability, and appropriateness are often discussed but not measured as service characteristics.	Link patient surveys with service audits, workforce data, referral metrics, cost data, and cultural safety indicators.
Interaction of dimensions	Barriers rarely operate in isolation; stigma, affordability, transport, and service fit may compound each other.	Use longitudinal pathway models, mixed methods, and causal diagrams to examine how dimensions interact over time.
Equity and intersectionality	Marginalised groups face compounded barriers that can be obscured by single-axis analyses; underrecorded or underexamined characteristics and missing routine demographic data can hide inequalities (13).	Design studies powered or purposively sampled for intersections of race, migration, language, age, gender, rurality, disability, income, religion, sexual orientation, and social capital; improve routine data completeness and patient self-report.
Intervention evaluation	Many proposed facilitators are descriptive rather than tested.	Evaluate integrated care, navigation, screening-to-referral pathways, digital tools, and culturally responsive training using access outcomes at multiple stages.
Framework fidelity and completeness	Incomplete or post hoc use limits comparability and may obscure the paired service–population structure of the framework.	Report a priori versus post hoc use, operational definitions, coding rules, modifications, overlapping dimensions, and whether all ten components were examined.
Representation of affected people	Caregiver, professional, and organizational accounts may describe different aspects of access from affected people’s own experiences; population-specific evidence is not necessarily first-person evidence.	Distinguish service users, informants, and decision-makers. Use accessible direct participation and separate or dyadic interviews; examine divergent accounts and include people who have not reached services.

## Discussion

The updated evidence base changes the interpretation of the youth and perinatal clusters. Youth evidence is no longer limited to a single diagnostic-pathway study and a conceptual commentary. Direct applications now span diagnostic timing, predominantly parent- and professional-informed accounts of access for racially and ethnically minoritized adolescents, primary-care consultation models, and service availability. Together, these studies show that youth access depends on the interaction of recognition by families and schools, the capability of first-contact providers, the accuracy of service information, referral and insurance arrangements, workforce capacity, and culturally acceptable care. The finding that better-staffed centers could still report longer waits, and that service directories could be inaccurate, illustrates why availability should not be inferred from nominal service presence alone.

The synthesis also challenges a narrow service-supply model of access. Adding services may not improve access if people cannot identify them, trust them, reach them, afford them, or remain engaged. Conversely, improving mental health literacy or screening may have limited value without referral capacity and appropriate care. Perinatal screening, for example, is a gateway to care only when accompanied by clear referral coordination and available, culturally responsive support [14–16]. This may be particularly important for migrant women, for whom broader maternity-care evidence points to culturally unsafe communication, discrimination, limited information, trust, and continuity as access-relevant mechanisms [40]. Similarly, teachers’ detection of ADHD symptoms can facilitate access, but only if school, primary care, and specialist pathways are linked [8]. For older adults, primary care and community services may be natural access points, but they require continuity, coordination, and accommodations for multimorbidity, cognitive limitations, and transport barriers [9, 17–19].

The framework’s life-course value lies in its ability to identify both shared and population-specific barriers. Stigma and low mental health literacy recur across all groups, but the content of stigma differs: fear of being judged as an incompetent mother in perinatal care, fear or shame around late-life mental illness among older adults, and confidentiality concerns among adolescents and young adults. Availability barriers are also population-specific: perinatal psychiatry workforce shortages, social psychiatric and integrated care gaps for older adults, and specialist diagnostic bottlenecks for youth ADHD. The framework can therefore support both general access theory and tailored intervention design.

The added value of the cross-population comparison lies in showing how the same nominal access dimension may be produced by different mechanisms. In the perinatal evidence, access was mediated through maternity–primary-care–mental-health interfaces and concerns about motherhood-related judgment. Youth access depended more strongly on caregivers, schools, developmental autonomy, and confidentiality-sensitive services. Later-life access was shaped by coordination across primary, geriatric, mental-health, community, and social care and by mobility, cognitive, and sensory accommodations. A label such as acceptability, availability, or ability to seek should therefore not be assumed to represent an identical mechanism across populations. Because the evidence base is small and uneven, particularly for youth and older adults, these patterns remain provisional comparative propositions.

A further distinction concerns whose perspective is represented. Classification as direct population-specific evidence indicates that a source addresses access for the eligible population; it does not necessarily indicate a first-person account of access. In the youth evidence, ADHD pathway ages were reported retrospectively by caregivers [8]; the qualitative adolescent study interviewed one adolescent, three parents, and six professionals [44]; the consultation-program evaluation elicited provider perspectives [45], while the facility survey addressed organizational conditions [46]. The sole direct older-adult study included two service users among ten interviewees in its mental-illness case group and excluded dementia [9]. Adjacent dementia evidence was also heterogeneous: one study interviewed caregivers and professionals without people with dementia themselves [42], whereas Bartlett et al. included people with dementia in two individual and twelve dyadic interviews [41].

Caregiver and professional accounts provide evidence about gatekeeping, service coordination, and the practical support needed to obtain care. Their interpretations may nevertheless differ from affected people’s experiences of stigma, confidentiality, autonomy, perceived need, or acceptable care. This distinction is particularly relevant when help-seeking and decisions are mediated by parents, institutions, or carers. Future access studies should identify separately the intended service user, the informant, and the person initiating or making care decisions. Developmentally appropriate and cognitively accessible interviews, supported communication, and analyses of agreement and divergence within dyads can improve representation of these perspectives. The examples identified here were not based on systematic respondent-type coding of all mapped sources and do not establish the comparative prevalence of proxy reporting across populations.

The fourth youth-focused Elicit report also added an important distinction between direct and adjacent evidence. Intervention research suggested that improvements in mental-health knowledge and attitudes did not automatically translate into realized service use, whereas appointment reminders and motivational strategies could facilitate first attendance [30]. Refugee-focused reviews showed how legal insecurity, unstable housing, language, stigma, cultural mismatch, financial pressure, and fragmented systems could accumulate across the access pathway [31, 49]. Adjacent empirical sources additionally illustrated challenges in navigating host-country health systems and accessing culturally appropriate services among refugee minors and minoritized ethnic youth [47, 48]. These sources did not all meet the strict 0–17-year criterion, but they strengthen the framework-based proposition that barriers are sequential, interacting, and shaped by intersecting social positions.

The expanded perinatal cluster further emphasizes that relational continuity can shape access as well as care quality. Continuity-of-care midwifery can create safe conditions for disclosure, provide information and advocacy, and support navigation, while broken referral pathways, limited specialist capacity, distance, cost, and discharge from midwifery care can interrupt access after need has been recognized. The rapid review indicates that stigma and access barriers are shared across women, midwives, and obstetricians and operate at individual, institutional, and societal levels. This supports a systems interpretation in which improving screening alone is unlikely to be sufficient without referral authority, trained staff, service capacity, and coordinated pathways. These interpretations are supported by the direct continuity-of-care study [33], the scoping review [16], and the rapid review [34].

The appraisal qualifies both intervention claims and the interpretation of unmet need. The consultation-program evaluation used anonymous baseline and follow-up samples that could not be linked and measured providers’ self-reported confidence and skill; it did not establish improved patient access or clinical outcomes [45]. Service-based recruitment can omit people whose pathway ended before contact [8], while incomplete facility responses and missing service information can obscure availability [46]. Mapping a reported facilitator therefore identifies a plausible intervention target unless its effect on access has been directly evaluated. These limitations make it especially important to distinguish observed service use, stakeholder explanations, and causal effects.

Across life-course stages, the added studies therefore sharpen the comparative contribution of the review. Perinatal access is strongly mediated by continuity, disclosure safety, maternity-service referral pathways, and specialist capacity. Youth access is mediated by developmental dependence, schools, families, primary-care capability, service information, insurance, and culturally appropriate specialist services. Later-life evidence remains the least developed and is shaped by coordination across health and social care and by mobility, cognitive, and sensory needs. The same Levesque label—such as approachability, availability, or appropriateness—thus represents different mechanisms and intervention targets across populations.

For health systems, the findings imply that interventions should target multiple stages of the access process. Single-stage interventions risk shifting bottlenecks downstream. Awareness campaigns may increase perceived need without increasing capacity; screening may identify distress without referral routes; insurance expansion may increase initial utilization without treatment intensity; and telehealth may improve reach while excluding people with low digital access or comfort. Multi-component interventions - such as integrated perinatal mental health pathways, primary care-based older-adult mental health screening with care navigation, school-linked diagnostic pathways, and culturally responsive community outreach - are more consistent with the Levesque model.

For research, the main priority is operationalization. Mental health access studies should define which Levesque [4] dimensions are being measured, specify whether outcomes reflect awareness, help-seeking, first contact, treatment initiation, affordability, continuity, or engagement, and test whether supply- and demand-side dimensions jointly predict access outcomes. Mixed-methods designs are especially promising because they can quantify pathways while preserving the lived experience and contextual detail that explain why barriers arise. This requires explicit integration of the qualitative and quantitative strands and adequate methods within each component; collecting both forms of data alone does not provide that integration.

The added framework-level evidence suggests that this operationalization challenge is not unique to mental health research. Across health care access studies using the Levesque model, researchers have reported partial framework use and category overlap [12]. Mental health access research should therefore treat the framework less as a checklist of isolated barriers and more as a pathway model in which early recognition, acceptability, reachability, affordability, and sustained engagement are empirically linked.

The adult mental health mapping review shows the same problem within mental health services: relying on contact or utilization data alone can conceal unmet need and pathway inequities, especially when routine demographic data are incomplete [13].

Table 5 summarizes the comparative interpretation of mental-health access across the selected life-course stages.

**Table 5:** Comparative interpretation of mental-health access at selected life-course stages

Comparative feature	Perinatal	Children/adolescents	Older adults
Barriers before formal service contact	Normalization of distress; limited awareness of services; fear of disclosure	Recognition by caregivers/teachers; community awareness; accurate service information; first-contact navigation	Low mental-health literacy; normalization in later life
Principal gatekeepers	Midwives, obstetricians, primary care, family	Parents/caregivers, teachers/schools, primary-care providers, consultation programs and referral systems	Family, primary care, community and social-care providers
Critical service interface	Maternity–midwifery/obstetrics–primary care–mental health	School–primary care–child psychiatry consultation–specialist mental health	Primary–geriatric–mental health–social care
Population-specific acceptability condition	Safe disclosure, fear of maternal judgment, cultural fit, continuity	Confidentiality and autonomy; racial/ethnic representation; trauma-informed, family-centered and culturally responsive care	Mental-health stigma, age-related expectations, trust
Conditions for sustained engagement	Continuity, advocacy, direct referral pathways, specialist capacity	Accurate navigation, insurance coverage, short waits, developmentally appropriate communication, reminders/motivational support	Continuity and mobility, cognitive and sensory accommodation
Evidential qualification	Three direct empirical studies and two direct reviews; still heterogeneous	Four direct empirical studies; caregiver-reported pathways, mainly stakeholder interviews, provider surveys, and facility data. First-person youth accounts were limited.	One direct empirical study with two service-user interviews; adjacent dementia studies varied in direct and supported participation.

### Strengths and limitations

A strength of this review is its use of a paired service–population framework to examine the stages of access from recognition of need through sustained engagement. The preregistered comparison of selected life-course stages made it possible to examine differences in gatekeepers, service interfaces, autonomy, and acceptability conditions. Separating direct empirical, review-level, adjacent, and contextual evidence also reduced the risk of treating sources with different levels of relevance as equivalent. Independent appraisal by JCC and BR, preservation of preconference ratings, and source-based consensus resolution strengthened the transparency of the methodological assessment.

Although the AI-assisted workflow is documented, documentation does not eliminate the methodological risks associated with semantic retrieval, relevance ranking, automated preliminary screening, and draft extraction. These procedures may be affected by corpus coverage, query wording, and platform updates and may result in missed records, inappropriate recommendations, or incomplete extraction. Complex extraction tasks are additionally vulnerable to errors in interpreting study constructs [50]. Misclassification of Levesque use remains a potential consequence for this review. An evaluation of Elicit found that extracted values, supporting quotations, and reasoning were not fully reproducible across user accounts and extraction modes [51]. We mitigated these risks through the available query and report documentation, forward citation tracking, a dedicated youth search, deduplication, human title and abstract screening, full-text assessment, and author-led Levesque coding and synthesis. Verification of retained sources could not establish how many eligible records had been missed during automated preselection. Study selection was not independently duplicated; residual selection and classification error therefore cannot be excluded. No complete record-level screening log was maintained. The reported aggregate counts and available retrospective source-specific decisions therefore cannot support a full independent audit of exclusions or reason-specific frequencies.

The prespecified Levesque framework guided the review from initial planning. Evidence identification began with Elicit-assisted reports and was supplemented by citation searches and human verification. These steps broadened the evidence base and addressed the original youth-search asymmetry, but do not establish comprehensive coverage of mental-health-access research in the three populations. The 2,423 citing records and population-filter counts were route-level search outputs, not unique human-screened records. Limitations of corpus coverage, automated preselection and incomplete historical documentation should inform interpretation of the mapped evidence.

The route-level arithmetic in Fig. 1 is consistent with the reported aggregate total, but does not independently verify record-level deduplication or the placement of every retained source within the screening flow. Some sources were identified through more than one route, and the source inventory also includes a predefined framework seed. Their complete relation to the historical screening counts cannot be reconstructed from the available documentation.

The fourth Elicit route also illustrated the importance of human follow-up when platform full text was unavailable. Of the ten records for which Elicit lacked full-text access, six were considered potentially relevant based on title and abstract, retrieved, and assessed in full text; three were retained as adjacent evidence, and three were excluded. The remaining four records were excluded during human title and abstract screening and were therefore not sought for retrieval. No report sought for retrieval remained unavailable.

The direct evidence base remained small and heterogeneous. The perinatal cluster comprised three empirical primary studies and two review-level sources; the youth cluster comprised four direct empirical studies that differed substantially in design and setting; and the older-adult cluster comprised only one direct empirical study. Qualitative studies provided essential contextual and experiential evidence and were not a limitation in themselves. Remaining limitations concerned incomplete representation and linking of service dimensions and population abilities, limited validated measurement, scarcity of longitudinal and pathway-level intervention evaluations, and the particularly sparse direct older-adult evidence base. Appraisal identified limitations in representativeness, confounder control, mixed-methods component quality, and review procedures. High inter-rater agreement indicates consistency in applying the appraisal criteria, not uniformly strong underlying evidence. The descriptive pooled κ combines different tools and study designs; the area-specific estimates provide the more differentiated account. Uneven representation of affected people’s own perspectives further limits comparisons of lived access experiences across populations. Funding of individual included sources was not systematically charted, so potential patterns related to source funding could not be assessed.

## Conclusion

Within a heterogeneous but expanded evidence base, the Levesque framework provided a useful comparative vocabulary for conceptualizing mental-health access as a pathway from recognition of need to sustained engagement. The updated perinatal and youth evidence shows that nominal service availability is insufficient when information is inaccurate, referral routes are fragmented, first-contact providers lack capacity, insurance or cost barriers persist, or care is not culturally and developmentally acceptable. The mapped patterns differ across selected life-course stages because access is mediated by different gatekeepers, service interfaces, and relational conditions. These observations remain exploratory and should not be interpreted as estimates of barrier prevalence or comparative importance. Future research should link population abilities with measurable service characteristics, evaluate pathway-level interventions, and report age-specific and intersectional findings that permit direct life-course comparison. It should also distinguish affected people’s accounts from caregiver and professional perspectives and enable direct participation where developmentally and cognitively appropriate.

## Supplementary Information

Below is the link to the electronic supplementary material.

Supplementary Material 1

Supplementary Material 2

Supplementary Material 3

Supplementary Material 4

Supplementary Material 5

Supplementary Material 6

Supplementary Material 7

Supplementary Material 8

Supplementary Material 9

Supplementary Material 10

Supplementary Material 11

Supplementary Material 12

## Data Availability

No datasets were generated or analysed during the current study.

## Declarations

### Preregistration

The review protocol was registered on the Open Science Framework on 6 February 2026 (https://osf.io/8ntrb/).

### AI use declaration

AI-assisted tools were used in evidence identification, preliminary screening and extraction, preparation of documentation, and manuscript drafting and revision. Elicit supported semantic retrieval, relevance ranking, application of predefined screening questions, and generation of preliminary structured extraction outputs. The authors defined the review questions, population boundaries, eligibility criteria, and analytical framework and made the final decisions concerning source inclusion, evidence classification, full-text verification, mapping to the Levesque dimensions, methodological appraisal, narrative synthesis, and interpretation. Generative AI tools—ChatGPT (OpenAI; GPT-5.5 Pro on 6 May 2026 and GPT-5.6 Pro during the July–August 2026 revision)—were used to support manuscript structuring, translation, linguistic refinement, preparation of transparency documentation, and organization of editor and reviewer comments. All AI-assisted outputs were reviewed, revised, or rejected by the authors. AI tools were not assigned responsibility for scientific decisions, source verification, eligibility, methodological appraisal, evidence classification, or conclusions. During the September 2026 revisions, including the revision on 24 September 2026 using GPT-6 Astra Ultra (author-reported), ChatGPT additionally supported appraisal-workbook analysis, calculation and checking of item-level agreement statistics based on human ratings, comparison of manuscript claims with source materials, and revision and consistency checking of the manuscript and supplementary materials; the authors retained responsibility for the analysis and interpretation. Available task-level documentation of the AI-assisted workflow (52), including tools and purposes, prompts or prompt summaries, output use, human oversight, source and claim verification, data-protection considerations, and unavailable historical metadata, is provided in Supplementary Material S3. Available Elicit research questions, screening criteria, extraction instructions, and report documentation are provided in Supplementary Material S4. Confidential editorial correspondence is not reproduced in publicly available prompt logs. The authors assume full responsibility for the accuracy, originality, integrity, and content of the manuscript and supplementary materials. The authors retained responsibility for checking these outputs and approving the final manuscript.
