# Supplementary material for: Patient-centered access to mental health care at selected life-course stages: a scoping review using the Levesque framework
Source: BMC Health Serv Res. 2026 Oct 22;26:1464. doi: 10.1186/s12913-026-15895-z (PMC13693167; doi:10.1186/s12913-026-15895-z)
Supplement: Supplementary file 1 [file 12913_2026_15895_MOESM1_ESM.pdf]

Which empirical studies (2013–present) apply the Levesque et al. (2013) patient-centred access to health care framework to examine mental health care access among older adults (aged 65 years and older)? Include quantitative, qualitative, or mixed-methods studies. The primary outcome must be mental health care access (e.g., access to psychiatric services, psychotherapy, counseling, geriatric mental health services, or community mental health programs). For each study, report: Study design Country Population characteristics (age range, mean age) Type of mental health service How the Levesque framework is used Which access dimensions are examined Main findings specifically related to mental health care access

Only three studies — Schwarz et al. (2021) in Austria, Kurpas et al. (2018) in Poland, and Dableh et al. (2025a) in Lebanon — directly apply the Levesque framework to mental health care access among older adults aged 65+, all using qualitative designs, and they converge on barriers of low mental health literacy, stigma, limited service availability, financial constraints, and inappropriate service design, but no quantitative studies applying the framework to this specific intersection were identified.

Abstract

Of 17 sources reviewed, only three studies directly apply the Levesque et al. (2013) patient-centred access framework to examine mental health care access among older adults aged 65 and older: Schwarz et al. (2021), a qualitative study of 25 experts in Austria focusing on older adults with pre-existing mental illness [1, 1, 1]; Kurpas et al. (2018), a qualitative focus group study of 44 participants including frail older adults aged 65+ in Poland examining psychological support needs [2, 2, 2]; and Dableh et al. (2025a), a qualitative study of 57 participants including community-dwelling older adults aged 60–92 in Lebanon addressing emotional and cognitive barriers to primary care access [3, 3, 3]. Two additional studies are closely aligned — Dableh et al. (2025) co-designed depression and cognitive screening solutions for older people in Lebanon [4, 4], and Bartlett et al. (2025) examined access to social farms for people with dementia in England [5, 5] — though neither confirmed participants as aged 65+. The remaining sources apply the framework to mental health access in non-older-adult populations (e.g., refugees [6], perinatal women [7], forensic service users [8]) or to general healthcare access in older adults without a primary mental health outcome [9, 10].

Across the directly relevant studies, consistent barriers to mental health care access for older adults emerge: low mental health literacy and limited ability to perceive need [1, 3, 4], stigma reducing care-seeking [1, 11], poor service availability and geographical or transport barriers [2, 3, 5], financial constraints [2, 3], and inappropriateness of existing services for older adults' complex needs [1–3]. All directly relevant studies are qualitative [1–3], and none provides quantitative access measures framed within the Levesque model. The evidence base applying this framework simultaneously to mental health care access and older adults aged 65+ is thus very limited, concentrated in European and Middle Eastern settings, and lacks quantitative operationalization — representing a substantial gap given the framework's demonstrated utility across other populations and health domains [12].

Flow Diagram

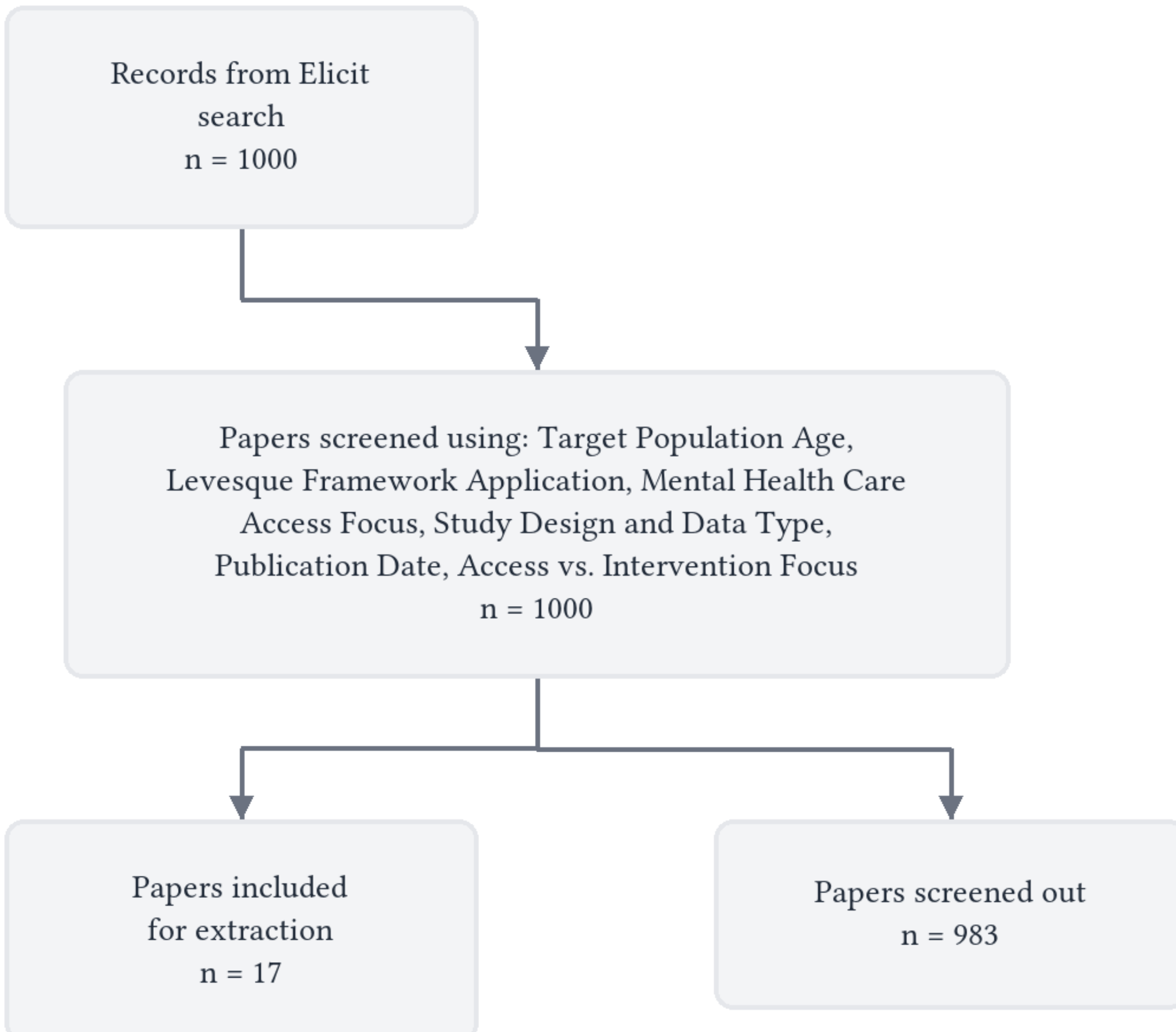

Paper search

We performed a semantic search across over 138 million academic papers from the Elicit search engine, which includes all of Semantic Scholar and OpenAlex.

We ran this query: "Which empirical studies (2013–present) apply the Levesque et al. (2013) patient-centred access to health care framework to examine mental health care access among older adults (aged 65 years and older)? Include quantitative, qualitative, or mixed-methods studies. The primary outcome must be mental health care access (e.g., access to psychiatric services, psychotherapy, counseling, geriatric mental health services, or community mental

health programs). For each study, report: Study design Country Population characteristics (age range, mean age) Type of mental health service How the Levesque framework is used Which access dimensions are examined Main findings specifically related to mental health care access"

The search returned 1,000 total results from Elicit.

We retrieved 1000 papers most relevant to the query for screening.

Screening

We screened in sources based on their abstracts that met these criteria:

- **Target Population Age**: Does this study focus on older adults aged 65 years and older, OR does it report results separately for participants ≥65 years, OR do older adults (≥65 years) comprise the majority (≥50%) of the study population?
- **Levesque Framework Application**: Does this study explicitly reference and apply the Levesque et al. (2013) patient-centred access to healthcare framework as a theoretical foundation, analytical framework, or organizing structure?
- **Mental Health Care Access Focus**: Does this study examine access to mental health services as a primary focus (including psychiatric services, psychotherapy, counseling, geriatric mental health services, community mental health programs, or other mental health interventions)?
- **Study Design and Data Type**: Is this study an empirical study using quantitative, qualitative, or mixed-methods approaches with original data, OR a systematic review/meta-analysis that synthesizes empirical studies?
- **Publication Date**: Was this study published in 2013 or later?
- **Access vs. Intervention Focus**: Is this study primarily focused on examining access to mental health care rather than solely evaluating the effectiveness of mental health interventions?

Papers that failed any strict criterion were automatically excluded. For the remaining papers, we considered all screening questions together and made a holistic judgement about whether to screen in each paper.

Data extraction

We asked a large language model to extract each data column below from each paper. We gave the model the extraction instructions shown below for each column.

- **Study Details**:

  Extract basic study information to confirm eligibility and provide context, including:

- Study design (quantitative, qualitative, mixed-methods)
- Country where study was conducted
- Year of publication
- Study time period/data collection dates
- Sample size

- **Older Adult Population**:

  Extract population characteristics specifically focusing on older adults (65+ years), including:

- Age range of participants

- Mean age (if provided)
- Percentage of sample that is 65+ years old
- Other relevant demographic characteristics (gender, ethnicity, socioeconomic status, rural/urban)
- Any specific older adult subgroups studied (e.g., community-dwelling, nursing home residents, those with dementia)

- **Mental Health Service Type**:

  Extract details about the specific mental health services or access issues examined in the study, including:

- Type of mental health service (psychiatric services, psychotherapy, counseling, geriatric mental health services, community mental health programs, etc.)
- Service setting (primary care, specialized mental health facilities, community-based, telehealth/virtual)
- Specific mental health conditions addressed (depression, anxiety, dementia-related behavioral issues, etc.)
- Level of care (prevention, screening, treatment, ongoing care)

- **Levesque Framework Application**:

  Extract how the Levesque et al. (2013) patient-centred access framework is applied in the study, including:

- Whether framework was used a priori (to design study/tools) or a posteriori (to organize/analyze data)
- Which of the 5 access dimensions are examined (approachability, acceptability, availability and accommodation, affordability, appropriateness)
- Which of the 5 population abilities are examined (ability to perceive, ability to seek, ability to reach, ability to pay, ability to engage)
- How the framework guided data collection, analysis, or interpretation
- Any modifications or adaptations made to the framework

- **Mental Health Access Findings**:

  Extract main findings specifically related to mental health care access among older adults, including:

- Key barriers to mental health access identified for older adults
- Facilitators or enablers of mental health access for this population
- Access outcomes or measures reported (utilization rates, unmet need, delays in care, satisfaction)
- Differences in access across older adult subgroups
- Policy or practice implications specifically for improving mental health access among older adults
- Any quantitative results (effect sizes, odds ratios, percentages) related to access

Results

Characteristics of Included Studies

This review identified 17 sources that apply or discuss the application of the Levesque et al. (2013) patient-centred access to health care framework in the context of health or mental health care access. The sources vary considerably in the degree to which they directly address the intersection of the Levesque framework, mental health care access, and older adults aged 65 years and older. The majority of studies do not simultaneously satisfy all three criteria specified in the research question; rather, they address one or two of these domains, providing indirect or partial evidence. The table below summarizes all included sources.

Study	Full Text Retrieved?	Study Design	Country	Population	Mental Health Service Focus	Levesque Framework Use	Relevance to Research Question
Amika Shah et al., 2025	No	Qualitative [11]	Canada [11]	13 PLWHF, 9 clinicians; age not specified [11]	General mental health services in heart failure remote management [11]	A posteriori; approachability, availability, affordability; ability to perceive, seek, reach, pay [11]	Indirect: mental health access but population not specified as 65+
Chandrika Ramakrishnan et al., 2025	Yes	Qualitative [13]	Singapore [13]	22 participants (caregivers, providers, policymakers); mean age 49 [13]	Community dementia care services [13]	A posteriori; all 5 dimensions and abilities examined [13]	Indirect: dementia care relevant to older adults, but participants are primarily caregivers
Kiran Saluja et al., 2025	Yes	Qualitative [14]	Canada [14]	32 primary care patients; majority over 50, age 65+ not specified [14]	Community-based mental health services (anxiety, panic attacks, addiction) [14]	A priori; all 5 dimensions and abilities; adapted for community resources [14]	Indirect: includes mental health but population not confirmed as 65+
Anthony Cu et al., 2021	Yes	Scoping review [12]	Multiple (71% HIC) [12]	31 studies reviewed; elderly mentioned in one study [12]	Not specified for mental health [12]	Both a priori (11 studies) and a posteriori (20 studies); all dimensions and abilities [12]	Indirect: framework review, minimal focus on older adults or mental health

Study	Full Text Retrieved?	Study Design	Country	Population	Mental Health Service Focus	Levesque Framework Use	Relevance to Research Question
M. Hynie et al., 2022	Yes	Mixed-methods [6]	Canada [6]	108 interviews; refugee newcomers aged 20–56 [6]	Virtual mental health services [6]	A priori; all 5 supply- and demand-side factors [6]	Not directly relevant: population is young refugees, not older adults
Hayley J. Lowther-Payne et al., 2023	Yes	Systematic mapping review [15]	UK [15]	152 studies; older adults mentioned but not the primary focus [15]	Adult NHS mental health services (secondary care, IAPT) [15]	A priori; all 5 stages of access and population abilities [15]	Indirect: covers mental health inequalities broadly, age is one dimension but older adults not specifically profiled
Marcia J Ash et al., 2025	No	Qualitative [7]	United States [7]	43 Black perinatal women [7]	Mental health services for depression and traumatic stress [7]	A posteriori; all 5 dimensions and abilities [7]	Not relevant: population is perinatal women
Lars Dumke et al., 2024	No	Scoping review of reviews [16]	High-income countries [16]	Refugees and asylum seekers; no older adult focus [16]	Mental health care for refugees [16]	A posteriori; approachability, acceptability, availability, appropriateness; ability to perceive, seek, reach, engage [16]	Not relevant: population is refugees/asylum seekers

Study	Full Text Retrieved?	Study Design	Country	Population	Mental Health Service Focus	Levesque Framework Use	Relevance to Research Question
Saydeh Dableh et al., 2025	No	Qualitative (co-design) [4]	Lebanon [4]	13 older people, family members, and providers; age not specified [4]	Screening for depression and cognitive problems at primary care [4]	Not explicitly described as using Levesque but references approachability, acceptability, appropriateness, availability, and abilities to perceive, seek, pay, engage [4]	Direct: older adults, mental health screening, and access framework aligned with Levesque dimensions
Abhijith A Kumar et al., 2025	No	Scoping review [9]	WHO Southeast Asia region [9]	39 articles on older adults; age not specified [9]	General healthcare; mental health not specifically mentioned [9]	A priori; dimensions not fully specified [9]	Indirect: older adults and Levesque but mental health not a primary focus
Sonja Maria et al., 2024	Yes	Scoping review (mixed-methods) [17]	US, Australia, Canada, Thailand, Brazil [17]	21 articles on LGBTQIA+ in rural areas; no older adult focus [17]	General mental healthcare in rural settings [17]	A posteriori; all 5 dimensions and abilities [17]	Not relevant: population is rural LGBTQIA+ individuals, not older adults
Marichelle C. Leclair et al., 2022	Yes	Quantitative [8]	Canada [8]	753 NCRMD individuals; age not specified [8]	Psychiatric services (inpatient and outpatient) [8]	A priori; seeking, reaching, receiving care [8]	Not relevant: forensic population, not older adults

Study	Full Text Retrieved?	Study Design	Country	Population	Mental Health Service Focus	Levesque Framework Use	Relevance to Research Question
D. Kurpas et al., 2018	Yes	Qualitative [2]	Poland [2]	44 participants including frail and non-frail older adults aged 65+ [2]	Psychological support/counseling for caregivers [2]	A posteriori; all 5 dimensions and abilities [2]	Direct: older adults 65+, Levesque framework, psychological support as mental health service
Ruth Bartlett et al., 2025	No	Mixed-methods [5]	England [5]	People with dementia; ethnicity noted (White British, South Asian); age not specified [5]	Community-based social farms for people with dementia [5]	A priori as navigational guide; suggested modification for intersectionality [5]	Direct: dementia population (likely older adults), social care as mental health-relevant service, Levesque framework
Andrea Mejía-Grueso et al., 2025	No	Quantitative [10]	Colombia [10]	Adults aged 60+; stratified by sex, region, residence, income [10]	General health services; mental health not specified [10]	A posteriori; all 5 abilities evaluated with composite score [10]	Indirect: older adults and Levesque, but mental health not a primary focus
T. Schwarz et al., 2021	Yes	Qualitative [1]	Austria [1]	25 expert interviews; older people with pre-existing mental illness (likely 65+) [1]	Mental health services for older adults with pre-existing mental illness (excl. dementia) [1]	A priori; all 5 dimensions and abilities [1]	Direct: older adults with mental illness, Levesque framework, mental health care access

Study	Full Text Retrieved?	Study Design	Country	Population	Mental Health Service Focus	Levesque Framework Use	Relevance to Research Question
Saydeh Dableh et al., 2025a	Yes	Qualitative [3]	Lebanon [3]	57 participants; older adults aged 60–92, 53% aged 71–79; community-dwelling [3]	General primary care; emotional and cognitive barriers discussed but not specific mental health services [3]	A priori; all 5 dimensions and abilities [3]	Partially direct: older adults 65+, Levesque, but mental health access is not the primary outcome

Among the 17 sources, only a small subset directly addresses all three criteria of the research question: application of the Levesque framework, a focus on mental health care access, and a population of older adults aged 65 years and older. Schwarz et al. (2021) is the most clearly aligned study, focusing explicitly on older adults with pre-existing mental illness in Austria and applying all five Levesque dimensions [1, 1]. Kurpas et al. (2018) examines frail older adults aged 65 and over in Poland and identifies psychological support needs as a key gap [2, 2]. Dableh et al. (2025) uses a co-design approach in Lebanon focusing on depression and cognitive screening for older people at primary healthcare centres [4, 4]. Dableh et al. (2025a) provides detailed data on community-dwelling older adults aged 60–92 accessing primary care in Lebanon, with emotional and cognitive barriers discussed, though mental health services per se are not the primary outcome [3, 3]. Bartlett et al. (2025) examines access to social farms for people with dementia in England, a population that is overwhelmingly older, using Levesque's framework as a navigational guide [5, 5]. Ramakrishnan et al. (2025) examines dementia care access in Singapore, though participants are principally caregivers rather than older adults themselves [13, 13].

The remaining sources contribute relevant but indirect evidence. Several focus on mental health access using the Levesque framework but in non-older-adult populations, including refugees [6, 16], forensic service users [8], perinatal women [7], and LGBTQIA+ communities [17]. Others examine older adults with the Levesque framework but do not target mental health services specifically [9, 10]. The scoping review by Cu et al. (2021) provides a methodological overview of Levesque framework applications across healthcare settings, noting only one study on the elderly and no specific focus on mental health [12, 12]. The systematic mapping review by Lowther-Payne et al. (2023) addresses mental health inequalities in the UK broadly but does not isolate older adults as a population [15, 15].

Thematic Analysis

Given that the included studies are predominantly qualitative or employ qualitative components, and the research question is oriented toward mapping the application of a conceptual framework rather than measuring a quantitative effect, a thematic analysis is presented below. Themes are organized around the Levesque framework's dimensions of access and corresponding population abilities, drawing on findings from the studies most relevant to older adults and mental health.

Approachability and Ability to Perceive

A recurring finding across studies is that older adults face substantial barriers related to the approachability of mental health services and their own ability to perceive mental health needs. In the Austrian context, Schwarz et al. (2021) reported that older adults with mental illness often lack perception of their own health needs, contributing to underutilization of services [1]. Shah et al. (2025) similarly identified low mental health literacy as a patient-level barrier among people living with heart failure, a population that skews older [11]. In Lebanon, Dableh et al. (2025) found that a lack of preventive strategies for mental health and cognitive abilities was the key access barrier identified by co-designers, leading to proposals for depression and cognitive screening at primary healthcare centres [4]. Dableh et al. (2025a) reinforced this finding, showing that perception of healthcare needs among older adults was enabled by acute symptoms, free services, literacy, and familial support, but hindered by a lack of information about available services [3]. In Singapore, Ramakrishnan et al. (2025) identified that lack of information and awareness limited the perceived need for dementia care services among caregivers [13]. The scoping review by Cu et al. (2021) confirmed that the ability to perceive was the most frequently represented population ability across all Levesque framework applications [12].

These converging findings suggest that across diverse cultural and healthcare contexts, information deficits and low mental health literacy constitute fundamental barriers to older adults recognizing and acting on mental health needs.

Acceptability and Ability to Seek

Stigma and cultural norms emerged as prominent barriers to seeking mental health care across multiple studies. Schwarz et al. (2021) identified stigma as a key barrier for older adults with mental illness in Austria [1]. Shah et al. (2025) reported stigma as a patient-level barrier reducing the ability to seek care among heart failure patients [11]. In Singapore, Ramakrishnan et al. (2025) found that cultural norms and values influenced the decision to seek dementia care services, with particular challenges for ethnic minority caregivers [13]. The UK-based review by Lowther-Payne et al. (2023) identified ”ability to seek” — specifically stigma and discrimination — as one of the most frequently cited barriers to adult mental health service access [15]. Among rural LGBTQIA+ populations, societal stigma and discrimination were also prominent deterrents to mental health service use [17]. In Lebanon, Dableh et al. (2025a) noted that providers' attitudes, poor service organization, and negative perceptions constrained healthcare seeking among older adults [3].

Fear and mistrust represent additional dimensions of the acceptability barrier. In the study of Black perinatal women, medical mistrust spanned several access dimensions and influenced both care-seeking and disclosure [7]. While this population is not older adults, the mechanism of mistrust is broadly applicable. In Lebanon, fear of medical treatment was noted as a barrier among older adults [3].

Availability, Accommodation, and Ability to Reach

Limited availability of services and logistical barriers to reaching care were identified as critical obstacles. Kurpas et al. (2018) documented poor availability of health and social care services for frail older adults in Poland, including high staff turnover, shortages of trained personnel, long waiting times for specialist and rehabilitation services, and geographically remote clinics [2]. Schwarz et al. (2021) reported inadequate social psychiatric services and poor coordination among providers as barriers for older adults in Austria [1]. In Lebanon, Dableh et al. (2025a) identified transport issues, mobility restrictions, staff and resource shortages, and service delivery challenges as barriers to reaching healthcare for older adults [3]. Bartlett et al. (2025) found that access to social farms for people with dementia in England was heavily dependent on independent travel means, with those accessing farms being overwhelmingly White British with access to a car or a carer who could drive [5].

The quantitative study by Leclair et al. (2022) — though not focused on older adults — demonstrated that geographical considerations were powerful determinants of reaching specialized mental health care: individuals living outside major urban centres were 2.6 times as likely to reach psychiatric services but also made 2.1 times greater use of emergency mental health services [8]. This pattern of geography mediating access to specialized mental health care is consistent with the qualitative findings in older adult populations, where rural remoteness and transportation difficulties repeatedly surface as barriers.

Virtual service delivery has been explored as a potential solution to availability barriers. Hynie et al. (2022) found that while virtual mental health services improved reach for some refugee populations, they also introduced challenges related to technology access and comfort, with older adults noted as particularly uncomfortable with camera use [6]. This suggests that telehealth solutions for older adults require careful design to account for digital literacy and technology access.

Affordability and Ability to Pay

Financial barriers to mental health care access were reported across multiple settings. Shah et al. (2025) identified lack of full insurance coverage and high cost of psychological services as patient-level barriers [11]. Kurpas et al. (2018) documented that treatment costs created barriers and inequities in the Polish system for frail older adults [2]. Ramakrishnan et al. (2025) found that financial constraints and high costs forced alternate care arrangements for dementia caregivers in Singapore [13]. In Lebanon, Dableh et al. (2025a) reported that while the economic recession drove older adults toward lower-cost public primary healthcare centres, lack of funds and financial resources still hindered utilization [3]. Saluja et al. (2025) identified high costs as a barrier for primary care patients accessing community resources, including mental health services, in Canada [14]. The Colombian study by Mejía-Grueso et al. (2025) found that individuals from higher-income households consistently experienced better access to healthcare services, with disparities persisting from 2015 to 2023 [10].

Appropriateness and Ability to Engage

Several studies highlighted that even when services are reached, their appropriateness for older adults with mental health needs is often inadequate. Kurpas et al. (2018) described an acute medical system inappropriate for patients with complex needs, alongside a low-functioning social care system, and recommended integration of health and social care with a care coordinator [2]. Schwarz et al. (2021) emphasized the importance of continuity of care and trust and suggested primary healthcare models and social prescribing as facilitators of appropriate engagement [1]. In Lebanon, Dableh et al. (2025a) found that positive relationships, literacy, and personal abilities facilitated healthcare engagement, while cognitive and sensory limitations, poor relationships, and lack of care continuity, coordination, comprehensiveness, and patient-centredness constrained it [3]. Bartlett et al. (2025) recommended incorporating intersectionality into the Levesque framework to better capture the diversity of access experiences among people with dementia, noting that access to specific facilities and activities within a service — not just to the service itself — matters for appropriateness [5].

The Dableh et al. (2025) co-design study in Lebanon resulted in two prototypes to improve appropriateness: a plan for depression and cognitive screening as a new primary care service, and a social media awareness campaign about preventive mental health strategies [4]. These co-designed solutions reflect an attempt to enhance both service-level appropriateness and population-level engagement capacity simultaneously.

Synthesis

The evidence base applying the Levesque framework to mental health care access among older adults aged 65 and older is remarkably sparse. Of 17 sources reviewed, only three — Schwarz et al. (2021), Kurpas et al. (2018), and Dableh et al. (2025a) — clearly include older adults aged 65 years and older and address mental health-related access using the Levesque framework [1, 1, 2, 2, 3, 3]. Two additional studies (Dableh et al., 2025; Bartlett et al., 2025) are closely aligned but either address older adults without specifying the age threshold [4] or focus on a condition (dementia) that predominantly affects older adults without confirming participants' ages [5]. Several other studies apply the framework to mental health access in non-older-adult populations or to general healthcare access in older adults, providing useful but indirect evidence.

The apparent scarcity of directly relevant empirical work has multiple explanations. First, the Levesque framework, while widely adopted since 2013, has been applied predominantly in maternal and child health, refugee health, and chronic disease contexts rather than in geriatric mental health [12]. Second, mental health access research among older adults has tended to use other theoretical models or no explicit framework at all, as suggested by the UK mapping review's finding that most studies on mental health inequalities relied on routinely collected utilization data rather than theoretically informed access measures [15]. Third, studies of older adult healthcare access using Levesque often subsume mental health within broader health service assessments rather than treating it as a distinct outcome [3, 9, 10].

Despite the limited number of directly applicable studies, the findings that do exist show notable consistency across countries and healthcare systems. Barriers cluster around low mental health literacy and limited ability to perceive need [1, 3, 4, 11], stigma reducing the ability to seek care [1, 11, 13], poor availability and geographical barriers reducing the ability to reach services [1–3, 5], financial constraints limiting the ability to pay [2, 3, 11, 13], and service designs inappropriate for the complex needs of older adults limiting meaningful engagement [1–3]. This consistency across Poland, Austria, Lebanon, England, Singapore, and Canada suggests that these barriers are not artifacts of particular healthcare systems but reflect structural features of how mental health services are organized and how aging intersects with help-seeking behaviour.

A methodological observation warrants attention. Nearly all studies with relevance to the research question employed qualitative designs [1–4], with the exception of Bartlett et al. (2025), which used mixed methods [5]. No study provided quantitative measures of mental health care access (e.g., utilization rates, odds ratios) specifically for older adults framed within the Levesque model. The quantitative study by Leclair et al. (2022) demonstrated that the Levesque framework can be operationalized for quantitative analysis of mental health service access [8], and the Colombian study by Mejía-Grueso et al. (2025) created composite access scores for older adults [10], but neither combined all three elements of the research question. This represents a significant methodological gap: the Levesque framework's multi-dimensional structure is well suited to quantitative measurement of access, yet this potential remains unrealized in geriatric mental health research.

The framework itself has been applied with varying degrees of completeness. Some studies examined all five dimensions and all five abilities [1–3], while others selectively examined a subset [8, 11]. Bartlett et al. (2025) proposed an important modification — incorporating intersectionality and within-service access to specific activities — to make the framework more suitable for social care research relevant to older adults [5]. This adaptation reflects a broader recognition that access to mental health-relevant services for older adults involves not only reaching a service but also navigating its internal offerings, which standard Levesque dimensions may not fully capture.

In summary, the direct application of the Levesque framework to mental health care access among older adults aged 65 and older remains an underdeveloped area of inquiry. The existing evidence, drawn primarily from qualitative studies in European and Middle Eastern settings, identifies a consistent set of multi-level barriers and points toward

integrated, person-centred service models with enhanced screening, care coordination, and attention to financial and geographic accessibility as potential facilitators. Quantitative operationalization of the Levesque framework for geriatric mental health access, and application in low- and middle-income country settings where population aging is accelerating, represent the most pressing gaps in the current evidence base.

References

1. T. Schwarz, A. Schmidt, J. Bobek, Joy Ladurner (2021) Barriers to Accessing Health Care for People with Chronic Conditions: A Qualitative Study. https://doi.org/10.21203/rs.3.rs-959131/v1

2. D. Kurpas, H. Gwyther, Katarzyna Szwamel, et al (2018) Patient-centred access to health care: a framework analysis of the care interface for frail older adults. BMC Geriatrics. https://doi.org/10.1186/s12877-018-0960-7

3. Saydeh Dableh, Kate Frazer, M. Azar, et al (2025) Barriers and enablers to primary health care center access for older people in Lebanon: A qualitative inquiry. PLoS ONE. https://doi.org/10.1371/journal.pone.0335073

4. Saydeh Dableh, Kate Frazer, M. Azar, et al (2025) Maximizing older people's access to primary health care centers in Lebanon: a co-design approach. Health Promotion International. https://doi.org/10.1093/heapro/daaf077

5. Ruth Bartlett, Alexandra Kaley, Nazmul Hussain, et al (2025) Access to Social Farms for People With Dementia Living at Home in England: A Mixed-Methods Analysis Using Levesque's Conceptual Framework. Health & Social Care in the Community. https://doi.org/10.1155/hsc/5593382

6. M. Hynie, Annie Jaimes, Anna Oda, et al (2022) Assessing Virtual Mental Health Access for Refugees during the COVID-19 Pandemic Using the Levesque Client-Centered Framework: What Have We Learned and How Will We Plan for the Future? International Journal of Environmental Research and Public Health. https://doi.org/10.3390/ijerph19095001

7. Marcia J Ash, B. Woods-Jaeger, Stella Udoetuk, et al (2025) Barriers and Facilitators to Accessing Mental Health Supports Among Black Perinatal Women: Application of the Patient-centered Access Framework. Journal of Racial and Ethnic Health Disparities. https://doi.org/10.1007/s40615-025-02428-3

8. Marichelle C. Leclair, Yanick Charette, M. Seto, et al (2022) Barriers and facilitators of access and utilization of mental health services among forensic service users along the care pathway. BMC Health Services Research. https://doi.org/10.1186/s12913-022-08848-9

9. Abhijith A Kumar, Anu Mohan, K. Rakshitha, et al (2025) Factors influencing healthcare access among older adults in Southeast Asia: a scoping review guided by the levesque framework. BMC Health Services Research. https://doi.org/10.1186/s12913-025-13838-8

10. Andrea Mejía-Grueso, Juliana Mejía-Grueso, Erika Geraldine Guarín Navas, et al (2025) Inequalities in access to health services among older adults in Colombia, 2015–2023. Cogent Gerontology. https://doi.org/10.1080/28324897.2025.2570145

11. Amika Shah, Anam Shahil Feroz, Robert P Nolan, et al (2025) Access to mental health services for people living with heart failure: a qualitative study. BMJ Open. https://doi.org/10.1136/bmjopen-2025-098866

12. Anthony Cu, Sofia Meister, Bertrand Lefebvre, V. Ridde (2021) Assessing healthcare access using the Levesque's conceptual framework– a scoping review. International Journal for Equity in Health. https://doi.org/10.1186/s12939-021-01416-3

13. Chandrika Ramakrishnan, Chetna Malhotra (2025) Challenges to accessing community dementia care services: a qualitative study. BMC Health Services Research. https://doi.org/10.1186/s12913-025-12895-3

14. Kiran Saluja, Adiba Mahbub, Alain Gauthier, et al (2025) Understanding patient barriers and enablers to accessing community resources: a qualitative study to inform navigation service delivery. BMC Primary Care. https://doi.org/10.1186/s12875-025-03029-z

15. Hayley J. Lowther-Payne, Anastasia Ushakova, Adelaide Beckwith, et al (2023) Understanding inequalities in access to adult mental health services in the UK: a systematic mapping review. BMC Health Services Research. https://doi.org/10.1186/s12913-023-10030-8

16. Lars Dumke, Sarah Wilker, Tobias Hecker, Frank Neuner (2024) Barriers to accessing mental health care for refugees and asylum seekers in high-income countries: A scoping review of reviews mapping demand and supply-side factors onto a conceptual framework. Clinical Psychology Review. https://doi.org/10.1016/j.cpr.2024.102491

17. Sonja Maria, Pauletta Irwin, P. Gillan, et al (2024) Navigating Mental Health Frontiers: A Scoping Review of Accessibility for Rural LGBTIQA+ Communities. Journal of Homosexuality. https://doi.org/10.1080/00918369.2024.2373798
