# Supplementary material for: Patient-centered access to mental health care at selected life-course stages: a scoping review using the Levesque framework
Source: BMC Health Serv Res. 2026 Oct 22;26:1464. doi: 10.1186/s12913-026-15895-z (PMC13693167; doi:10.1186/s12913-026-15895-z)
Supplement: Supplementary file 2 [file 12913_2026_15895_MOESM2_ESM.pdf]

Which empirical studies (2013–present) apply the Levesque et al. (2013) patient-centred access to health care framework to examine mental health care access among pregnant, perinatal, or postpartum women? Include quantitative, qualitative, or mixed-methods studies. The primary outcome must be mental health care access (e.g., access to psychiatric services, psychotherapy, counseling, perinatal mental health programs). For each study, report: Study design Country Population (pregnant, perinatal, postpartum) Type of mental health service How the Levesque framework is used Which access dimensions are examined Main findings related specifically to mental health care access Study design Population (pregnant, perinatal, postpartum) Mental health setting How the Levesque framework is used (citation only, conceptual framing, operationalization, measurement, intervention design, etc.) Which access dimensions (Approachability, Acceptability, Availability, Affordability, Appropriateness; Ability to perceive, seek, reach, pay, engage) are analyzed

Five empirical studies (Ash et al., 2025; Salameh et al., 2024; Viveiros et al., 2019; Berger et al., 2020; de Jong-Bardelmeijer et al., 2025) explicitly apply the Levesque framework to perinatal mental health care access, all using it as a conceptual or analytical lens rather than a validated measurement tool, and consistently finding that approachability, acceptability, and availability are the most compromised dimensions — driven by normalization of perinatal distress, stigma, cultural and language barriers, workforce shortages, and fragmented referral pathways — though the overall evidence base for this specific framework-population intersection remains small and largely qualitative.

Abstract

Of 31 sources screened, only five studies explicitly applied the Levesque et al. (2013) patient-centred access framework to examine mental health care access among perinatal women: Ash et al. (2025) used it to analyze focus group data from Black perinatal women in the U.S. [1], Salameh et al. (2024) structured thematic analysis of Syrian refugee women's barriers to postpartum depression services around its five supply-side dimensions [2], Viveiros et al. (2019) mapped barriers from 26 publications onto the framework's ten dimensions in a scoping review [3], Berger et al. (2020) used it to design both interview guides and analysis for a Swiss study of perinatal mental disorder care [4], and de Jong-Bardelmeijer et al. (2025) derived determinants from it for a quantitative analysis of postpartum healthcare utilization in the Netherlands [5]. All five employed the framework as a conceptual or analytical lens rather than operationalizing it with validated measurement scales. Approachability and ability to perceive were the most frequently compromised dimensions, with normalization of perinatal distress [3, 4], lack of awareness of services [2, 5], and stigma [4, 6] identified as near-universal demand-side barriers. Acceptability barriers were especially prominent for refugee and minority populations, including language barriers, cultural mismatch, and medical mistrust [1, 2, 7]. Availability constraints — including workforce shortages, broken referral pathways, and long wait times — were documented across settings [3, 8, 9].

The broader literature not applying the Levesque framework corroborated these patterns and provided additional

quantitative context: fewer than 20% of women with postpartum depression or anxiety access interventions [10], 21 U.S. states have fewer than 1 perinatal psychiatrist per 5,000 births [8], and policy interventions targeting affordability increased psychotherapy utilization but not treatment intensity [11]. Proposed facilitators include integrated care models [12], digital health interventions [10], culturally responsive training [1, 13], and relationship-centred care [14], though evidence for their effectiveness remains predominantly descriptive. The empirical application of the Levesque framework in perinatal mental health remains limited in both quantity and depth, with no studies testing causal pathways between framework dimensions and access outcomes, highlighting a gap between the framework's theoretical utility and its current operationalization in this field.

Flow Diagram

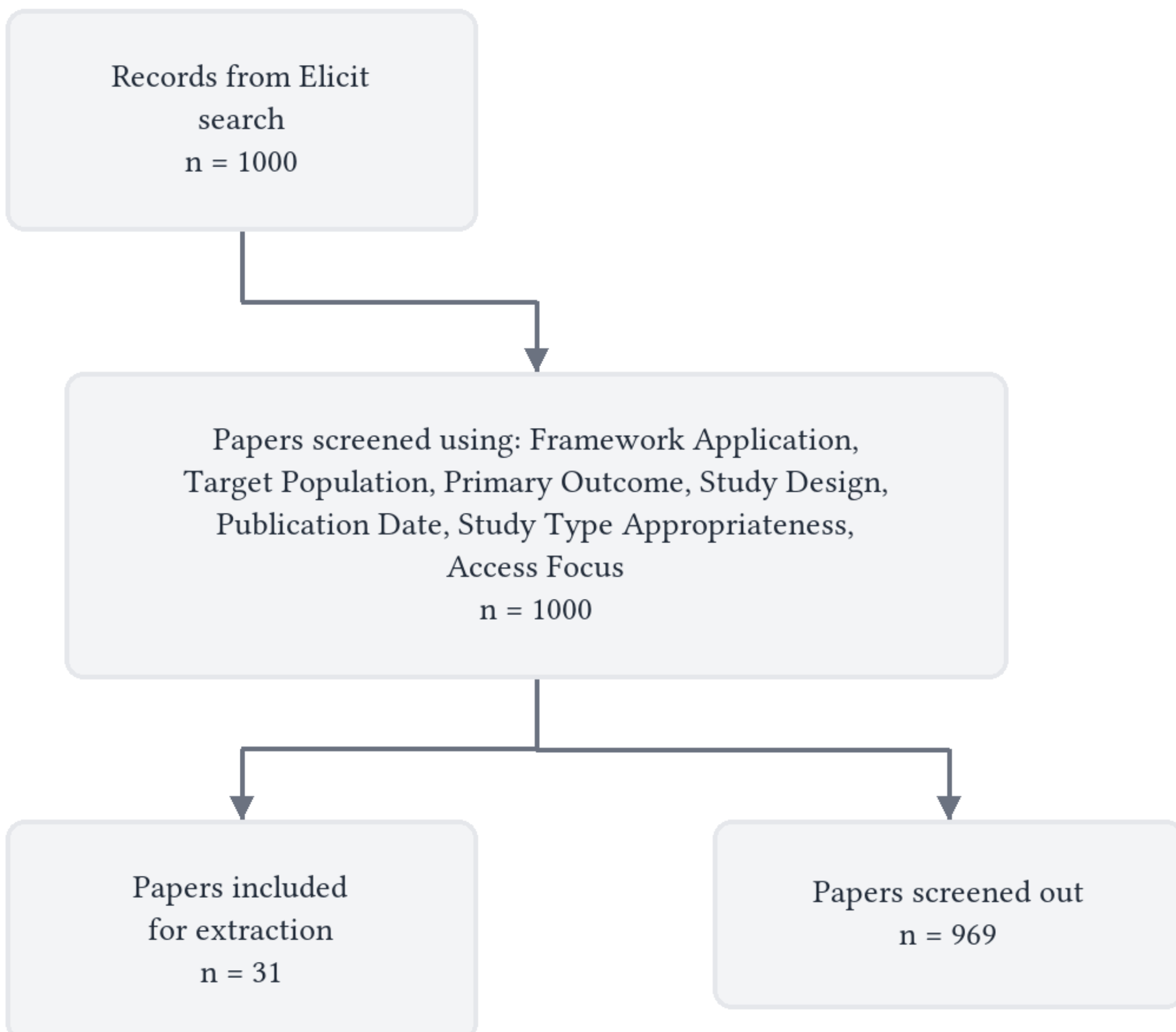

Paper search

We performed a semantic search across over 138 million academic papers from the Elicit search engine, which includes all of Semantic Scholar and OpenAlex.

We ran this query: "Which empirical studies (2013–present) apply the Levesque et al. (2013) patient-centred access to health care framework to examine mental health care access among pregnant, perinatal, or postpartum women? Include quantitative, qualitative, or mixed-methods studies. The primary outcome must be mental health care access (e.g., access to psychiatric services, psychotherapy, counseling, perinatal mental health programs). For each study, report: Study design Country Population (pregnant, perinatal, postpartum) Type of mental health service How the Levesque framework is used Which access dimensions are examined Main findings related specifically to mental health care access Study design Population (pregnant, perinatal, postpartum) Mental health setting How the Levesque framework is used (citation only, conceptual framing, operationalization, measurement, intervention design, etc.) Which access dimensions (Approachability, Acceptability, Availability, Affordability, Appropriateness; Ability to perceive, seek, reach, pay, engage) are analyzed"

The search returned 1,000 total results from Elicit.

We retrieved 1000 papers most relevant to the query for screening.

Screening

We screened in sources based on their abstracts that met these criteria:

- **Framework Application**: Does this study explicitly apply, reference, or utilize the Levesque et al. (2013) patient-centred access to healthcare framework?
- **Target Population**: Does this study focus on pregnant women, women during the perinatal period, or postpartum women?
- **Primary Outcome**: Is mental health care access a primary outcome in this study (including access to psychiatric services, psychotherapy, counseling, or perinatal mental health programs)?
- **Study Design**: Is this an empirical study using quantitative, qualitative, or mixed-methods approaches, or a systematic review/meta-analysis?
- **Publication Date**: Was this study published in 2013 or later?
- **Study Type Appropriateness**: Is this study NOT a theoretical paper, commentary, editorial, opinion piece, case report, case series with fewer than 10 participants, or conference abstract without full-text availability?
- **Access Focus**: Does this study focus on access to mental health care rather than primarily evaluating treatment effectiveness or clinical outcomes?

Papers that failed any strict criterion were automatically excluded. For the remaining papers, we considered all screening questions together and made a holistic judgement about whether to screen in each paper.

Data extraction

We asked a large language model to extract each data column below from each paper. We gave the model the extraction instructions shown below for each column.

- **Study Design**:

  Extract study design and methodology, including:

- Research design (qualitative, quantitative, mixed-methods, systematic review, meta-analysis, etc.)

- Data collection methods
- Sample size
- Study timeframe

- **Population Characteristics**:

  Extract details about the study population, including:

- Specific perinatal stage (pregnant, perinatal, postpartum, or combination)
- Gestational timing if specified (e.g., first trimester, within 12 months postpartum)
- Any special population characteristics (e.g., refugees, adolescents, specific ethnic groups, high-risk)
- Geographic location/country

- **Mental Health Services**:

  Extract the type of mental health services or care being accessed, including:

- Specific services (psychiatric services, psychotherapy, counseling, perinatal mental health programs, screening, etc.)
- Service setting (primary care, specialty mental health, community-based, digital/telehealth)
- Level of care (prevention, treatment, ongoing support)

- **Levesque Framework Application**:

  Extract specifically how the Levesque et al. (2013) patient-centred access framework is used in the study, including:

- Citation only vs. conceptual framing vs. operationalization vs. measurement tool
- Whether used for data collection design, analysis framework, or interpretation
- How explicitly the framework guides the study methodology
- Any modifications or adaptations made to the original framework

- **Framework Dimensions Examined**:

  Extract which specific dimensions of the Levesque framework are analyzed, using the 10-dimension model: Supply-side: Approachability, Acceptability, Availability & Accommodation, Affordability, Appropriateness Demand-side: Ability to perceive, Ability to seek, Ability to reach, Ability to pay, Ability to engage

- List all dimensions explicitly studied
- Note if study focuses on subset or examines all dimensions
- Identify any dimensions that are primary vs. secondary focus

- **Access Barriers**:

  Extract barriers to mental health care access identified in the study, organized by Levesque framework dimensions where possible, including:

- Specific barriers within each framework dimension
- Whether barriers are individual, system-level, or societal
- Any barriers unique to the perinatal period or specific to mental health (vs. general healthcare) access

- **Access Facilitators**:

  Extract facilitators or enablers of mental health care access identified in the study, including:

- Specific facilitators within each framework dimension
- Strategies or interventions that improve access
- System-level or policy solutions identified
- Any facilitators specific to perinatal mental health care

- **Key Findings**:

  Extract the main findings specifically related to mental health care access among pregnant, perinatal, or postpartum women, including:

- Primary conclusions about access patterns or problems
- Quantitative results (if applicable) such as access rates, effect sizes, statistical significance
- Qualitative themes or insights about access experiences
- Recommendations for improving access
- Any findings that advance understanding of the Levesque framework in this context

Results

Characteristics of Included Studies

The search identified 31 sources spanning a range of study designs, populations, and geographic contexts. These sources were examined for their application of the Levesque et al. (2013) patient-centred access framework to mental health care access among pregnant, perinatal, or postpartum women. The table below summarizes the characteristics of all identified studies.

Study	Full Text Retrieved?	Study Design	Population	Country/Setting	Perinatal Stage	Mental Health Service Focus	Levesque Framework Use
Ash et al. (2025)	No	Qualitative (focus groups) [1]	43 Black perinatal women [1]	Southeastern U.S. [1]	Pregnant and postpartum [1]	Culturally responsive trauma screening and services [1]	Conceptual framing; analysis framework [1]
Salameh et al. (2024)	No	Descriptive qualitative (semi-structured telephone interviews) [2]	15 Syrian refugee women [2]	Not specified [2]	Postpartum [2]	Psychosocial services, PPD screening [2]	Conceptual framing; analysis framework [2]

Study	Full Text Retrieved?	Study Design	Population	Country/Setting	Perinatal Stage	Mental Health Service Focus	Levesque Framework Use
Viveiros et al. (2019)	No	Scoping review (26 publications) [3]	Midwives and midwifery clients [3]	High-resource settings [3]	Perinatal [3]	Screening, PMH discussion [3]	Conceptual framing; mapping barriers onto framework [3]
Berger et al. (2020)	Yes	Qualitative (interviews and focus groups); protocol [4]	Women with PMD history (n=24) and providers (n=18–24) [4]	Switzerland [4]	Perinatal [4]	Psychiatric services, psychotherapy, counseling [4]	Conceptual framing; data collection design and analysis framework [4]
de Jong-Bardelmeijer et al. (2025)	No	Cross-sectional survey (n=1268) [5]	Postpartum women [5]	Netherlands [5]	Postpartum (first year) [5]	General postpartum health care including counseling [5]	Conceptual framing; complementary analysis model [5]
Sambrook Smith et al. (2019)	Yes	Systematic review and meta-synthesis (35 studies) [13]	Women with perinatal mental illness, families, HCPs [13]	United Kingdom [13]	Perinatal [13]	Psychosocial therapies, PMH programs, screening [13]	Not used [13]
Zivin et al. (2024)	Yes	Cross-sectional (837,316 deliveries) [11]	Women with PMADs [11]	United States [11]	Perinatal (12 months pre/post delivery) [11]	Psychotherapy [11]	Not used [11]
Webb et al. (2023)	Yes	Meta-review (32 reviews) [6]	Perinatal women including marginalized groups [6]	25 countries [6]	Perinatal [6]	CBT, psychological therapies, medications, screening [6]	Not used [6]

Study	Full Text Retrieved?	Study Design	Population	Country/Setting	Perinatal Stage	Mental Health Service Focus	Levesque Framework Use
Kingston et al. (2014)	Yes	Cross-sectional telephone survey (n=1207) [15]	General adult population [15]	Alberta, Canada [15]	General public views on perinatal MH [15]	Screening, counseling [15]	Not used [15]
Fisher et al. (2024)	Yes	Qualitative (139 semi-structured interviews) [14]	Women with moderate-severe MH disorders [14]	England [14]	Pregnancy to 24 months postpartum [14]	Specialist perinatal MH assessment and treatment [14]	Not used [14]
O'Mahony et al. (2021)	No	Qualitative systematic review protocol [16]	Perinatal refugee women [16]	Not specified [16]	Perinatal [16]	Mental health care services and social supports [16]	Not mentioned [16]
DeRoche et al. (2023)	Yes	Qualitative online survey (n=435) [9]	Healthcare providers [9]	Canada [9]	Perinatal (provider perspective) [9]	Screening, CBT, collaborative care [9]	Not used [9]
Rahim et al. (2018)	No	Commentary on systematic review [17]	Perinatal women [17]	UK [17]	Perinatal [17]	Not specified [17]	Not mentioned
Baumgartner et al. (2015)	No	Commentary [18]	Pregnant women [18]	Nigeria [18]	Pregnant [18]	Church-based MH screening [18]	Not used [18]
Wilson et al. (2024)	No	Global perspective/narrative review [19]	Perinatal populations [19]	Five continents [19]	Perinatal [19]	Not specified [19]	Not used [19]
Bali et al. (2024)	Yes	Systematic review and meta-synthesis (11 studies, 472 participants) [7]	African-born women in Australia [7]	Australia [7]	Maternity care (not MH-specific) [7]	Not MH-specific [7]	Conceptual framing; analysis framework [7]

Study	Full Text Retrieved?	Study Design	Population	Country/Setting	Perinatal Stage	Mental Health Service Focus	Levesque Framework Use
Dougherty et al. (2020)	Yes	Systematic scoping review (9 articles) [20]	Non-English speaking migrant families [20]	Primarily Australia [20]	Postpartum [20]	Not MH-specific [20]	Conceptual framing; analysis framework [20]
Collado et al. (2025)	No	Quantitative survey (n=674) [21]	Spanish-speaking perinatal Latinas [21]	United States [21]	Perinatal [21]	Peer-led MH support [21]	Not used [21]
Cox et al. (2017)	No	Program description [12]	Perinatal women [12]	Not specified [12]	Perinatal [12]	Integrated perinatal psychiatry [12]	Not used [12]
Koire et al. (2024)	Yes	Cross-sectional (all US states) [8]	Peripartum women [8]	United States [8]	Peripartum [8]	Community-based perinatal psychiatry [8]	Not explicitly used [8]
Weidner et al. (2017)	No	Quantitative (n=147) [22]	Pregnant and postpartum women [22]	Not specified (Germany implied) [22]	Pregnant and postpartum [22]	Specialized psychotherapy consultation [22]	Not used [22]
Narumoto et al. (2024)	No	Cross-sectional [23]	PCPs (provider perspective) [23]	Japan [23]	Postpartum [23]	Primary care-based postpartum MH [23]	Not used
Lewkowitz et al. (2024)	No	Systematic review and meta-analysis of RCTs [10]	Postpartum women [10]	United States [10]	Postpartum (first year) [10]	Digital health interventions [10]	Not used [10]
O'Mahony et al. (2018)	No	Environmental scan [24]	Immigrant women [24]	Western Canada [24]	Postpartum [24]	Community-based MH support [24]	Not used [24]

Study	Full Text Retrieved?	Study Design	Population	Country/Setting	Perinatal Stage	Mental Health Service Focus	Levesque Framework Use
Gardner et al. (2024)	No	Not specified [25]	Ethnic minority women [25]	United Kingdom [25]	Perinatal [25]	Specialist perinatal MH services [25]	Not mentioned
El Alam et al. (2025)	Yes	Qualitative (semi-structured interviews, n=17) [26]	Pregnant women of diverse back-grounds [26]	Lebanon [26]	Pregnant [26]	Antenatal care (not MH-specific) [26]	Conceptual framing and opera-tionaliza-tion; analysis framework [26]
Blackmore et al. (2021)	Yes	Mixed-methods case study [27]	Women of refugee back-ground [27]	Melbourne, Australia [27]	Pregnant [27]	Perinatal MH screening (iCOPE platform) [27]	Not used (COM-B model applied) [27]
Shah et al. (2024)	No	Not specified	Refugee women and birthing people [28]	UK [28]	Perinatal [28]	Perinatal MH services [28]	Not used
Pullyblank et al. (2023)	No	Mixed-methods (survey n=500, interviews) [29]	General healthcare patients (not perinatal-specific) [29]	Not specified (rural US implied) [29]	Not perinatal [29]	Telehealth [29]	Conceptual framing [29]
Triandafilidis et al. (2025)	No	Qualitative (interviews; 8 providers, 6 women) [30]	Women affected by domes-tic/family violence [30]	Australia [30]	Not perinatal-specific [30]	Primary care outreach (trauma-informed) [30]	Conceptual framing for acceptabil-ity [30]

Study	Full Text Retrieved?	Study Design	Population	Country/Setting	Perinatal Stage	Mental Health Service Focus	Levesque Framework Use
Xu et al. (2021)	No	Qualitative (semi-structured interviews, n=32) [31]	Postpartum women with traumatic birth [31]	Not specified [31]	Postpartum [31]	PTSD, depression, anxiety screening and support [31]	Not used [31]

Of the 31 sources identified, only a small subset explicitly applied the Levesque et al. (2013) framework to examine mental health care access among perinatal women. Five studies used the framework as a conceptual or analytical tool in the context of perinatal mental health specifically: Ash et al. (2025) [1], Salameh et al. (2024) [2], Viveiros et al. (2019) [3], Berger et al. (2020) [4], and de Jong-Bardelmeijer et al. (2025) [5]. Several additional studies applied the Levesque framework but focused on general maternity care rather than mental health specifically, including Bali et al. (2024) [7], Dougherty et al. (2020) [20], and El Alam et al. (2025) [26]. Two studies applied the framework outside the perinatal context altogether: Pullyblank et al. (2023) [29] examined telehealth access in a rural general population, and Triandafilidis et al. (2025) [30] investigated acceptability of services for women affected by domestic violence. The remaining sources either did not reference the Levesque framework or used alternative frameworks (e.g., Blackmore et al. [2021] employed the COM-B model [27]; Sambrook Smith et al. [2019] used a multilevel conceptual model [13]).

Study designs ranged from qualitative primary studies using interviews or focus groups [1, 2, 4, 14, 26, 31] to large-scale quantitative cross-sectional studies [5, 8, 11, 15], systematic reviews and meta-syntheses [3, 6, 7, 10, 13, 20], and mixed-methods approaches [27, 29]. Sample sizes varied considerably, from 14 participants in small qualitative studies [30] to over 837,000 deliveries in insurance claims analyses [11]. Geographic coverage was broad, encompassing studies from the United States [1, 8, 11, 21], the United Kingdom [13, 25, 28], Canada [9, 15, 24], Australia [7, 20, 27], Switzerland [4], the Netherlands [5], Japan [23], Lebanon [26], Nigeria [18], and multi-country reviews [6, 19]. Several studies specifically focused on marginalized populations, including Black perinatal women [1], Syrian refugees [2], immigrant and refugee women [7, 16, 24, 27, 28], Spanish-speaking Latinas [21], and ethnic minority women [25].

Thematic Analysis

Given the predominantly qualitative nature of the included evidence, the findings are organized thematically according to the Levesque framework dimensions and cross-cutting themes that emerged across studies.

Application of the Levesque Framework in Perinatal Mental Health Research

Among studies that explicitly applied the Levesque framework, the depth of application varied. Ash et al. (2025) used the framework to inform coding and analysis of focus group data, identifying barriers and facilitators across all five supply-side dimensions and corresponding demand-side abilities [1, 1]. Salameh et al. (2024) similarly structured their thematic analysis according to the five supply-side dimensions (approachability, acceptability, availability,

affordability, appropriateness), identifying 14 subthemes [2, 2]. Viveiros et al. (2019) mapped barriers from 26 publications onto the framework's ten dimensions, determining which points along the access chain were most adversely impacted [3, 3]. Berger et al. (2020) went further by using the framework to inform both interview guide design and thematic analysis, examining all ten dimensions [4, 4]. De Jong-Bardelmeijer et al. (2025) derived seven determinants from the Levesque framework as a complement to Andersen's behavioral model and used logistic regression to analyze them [5, 5].

Notably, no study reported modifying or adapting the original Levesque framework, and none operationalized the framework as a measurement tool with validated scales. The predominant mode of use was as a deductive coding or interpretive lens for qualitative or descriptive data.

Approachability and Ability to Perceive

Problems at the earliest stage of the access pathway — approachability and the ability to perceive a need for care — were among the most frequently identified barriers. Viveiros et al. (2019) found that 21 of 26 publications identified barriers at the beginning of the care-accessing process, suggesting that perinatal mental health care is often unapproachable or that women are unable to perceive their need for care [3]. Salameh et al. (2024) identified lack of knowledge about postpartum depression, misconceptions about treatment, and lack of awareness of available psychosocial services as barriers under approachability [2]. De Jong-Bardelmeijer et al. (2025) reported that unawareness of health care services significantly reduced the odds of utilization (OR 0.40; 95% CI 0.25–0.67) [5].

From the demand side, normalization of perinatal mental health concerns as expected symptoms of pregnancy was a recurring theme [3, 4]. Both Viveiros et al. (2019) and Berger et al. (2020) noted that women commonly attribute mood changes to the adjustment to motherhood, thereby failing to recognize symptoms warranting clinical attention [3, 4]. Webb et al. (2023), while not explicitly applying the Levesque framework, corroborated this finding, identifying lack of understanding about perinatal mental illness and not knowing the role of healthcare professionals as individual-level barriers [6]. Collado et al. (2025) similarly found that low awareness of when to seek help was a primary barrier among Spanish-speaking Latinas [21].

Fear of stigma further compounded these approachability barriers. Berger et al. (2020) identified stigma related to mental illness and feelings of shame as impediments [4], while Fisher et al. (2024) reported fear of judgement as inhibiting initial help-seeking [14]. Webb et al. (2023) highlighted a fear specific to the perinatal context: concern about being judged as a "bad mum" or triggering social services involvement [6]. Kingston et al. (2014) found that stigma associated with perinatal mental health problems and fear of being perceived as an incompetent mother were significant deterrents [15].

Acceptability and Ability to Seek

Cultural and societal norms shaped the acceptability of mental health services across multiple populations. Salameh et al. (2024) found that being a refugee, mental illness stigma, cultural preferences for certain healthcare providers, and language barriers all fell under acceptability concerns [2]. Ash et al. (2025) identified medical mistrust as a pervasive theme spanning several framework dimensions, particularly affecting the likelihood of seeking care and comfort disclosing mental health concerns [1, 1]. Berger et al. (2020) noted lower acceptability of pharmacotherapy during pregnancy and breastfeeding and the societal perception that mothers with severe mental disorders are incapable of caring for their children [4].

Among studies not applying the Levesque framework but addressing the same dimension, Sambrook Smith et al. (2019) identified language barriers and cultural differences as sociocultural barriers in the UK [13]. Gardner et al. (2024) reported that ethnic minority women perceived perinatal services as lacking cultural sensitivity [25]. DeRoche

et al. (2023) documented systemic racism and lack of culturally sensitive care for marginalized groups in Canada, with ethnocentric services and lack of services in languages other than English [9]. Kingston et al. (2014) found that the general public preferred counseling over medication as treatment for perinatal mental health problems, with fear of being prescribed medication as the only option serving as a deterrent [15].

The role of trust and relationship building emerged as a critical facilitator of acceptability. Fisher et al. (2024) identified four key pillars for engagement with community perinatal mental health services: perinatal competence, relationship building, accurate reassurance, and reliability [14]. Compassion, understanding, and consistency in interactional style were essential, with their impact varying by women's context and personal characteristics [14]. Bali et al. (2024), focusing on maternity care for African-born women in Australia, found that feeling valued by healthcare providers and cultural assimilation facilitated access [7].

Availability, Accommodation, and Ability to Reach

System-level availability constraints were documented extensively. Koire et al. (2024) quantified a severe shortage of perinatal psychiatrists in the US, finding that 21 states had fewer than 1 psychiatrist per 5,000 births [8]. This scarcity correlated with higher volumes of Google searches for postpartum depression and was associated with restrictive abortion policies [8]. Salameh et al. (2024) identified transportation barriers, location of services, lack of childcare support, and lack of time as availability-related barriers for refugee women [2]. Viveiros et al. (2019) documented broken referral pathways, lack of specialized services, and inefficiently long wait lists [3]. DeRoche et al. (2023) reported that over 50% of Canadian perinatal care providers lacked specialized mental health training and 87% were not mandated to screen [9].

Sambrook Smith et al. (2019) characterized resource inadequacies and service fragmentation as organizational-level barriers in the UK [13]. Webb et al. (2023) identified logistical barriers including travel costs, lack of childcare, and inconvenient timing of services [6]. Lewkowitz et al. (2024) reported that fewer than 20% of women with postpartum depression or anxiety access interventions, attributing this primarily to the shortage of trained mental health professionals [10].

From the demand side, Berger et al. (2020) noted that lack of affordable childcare affected women's ability to reach care [4], while El Alam et al. (2025) documented time constraints, transportation issues, and staff shortages as logistical challenges [26]. Xu et al. (2021) found that system-level barriers involving lack of communication, education, and resources prevented obstetric professionals from adequately supporting women after traumatic births [31].

Several studies identified strategies to address availability. Digital health interventions were proposed by Lewkowitz et al. (2024) as scalable solutions that overcome barriers such as lack of childcare and unreliable transportation [10]. Cox et al. (2017) described an integrated care model embedding a psychiatric nurse-practitioner in an obstetric clinic, enabling easy referral by perinatal nurses [12]. Weidner et al. (2017) evaluated a specialized consultation service with short waiting times, achieving a 78.4% uptake rate for recommended psychotherapy [22]. Blackmore et al. (2021) implemented a digital screening platform (iCOPE) alongside collaboration with refugee health services and designation of a ”go-to” referral person [27].

Affordability and Ability to Pay

Financial barriers were consistently reported across diverse settings. Salameh et al. (2024) documented financial difficulties and inadequate health insurance coverage among Syrian refugee women [2]. El Alam et al. (2025) described financial constraints exacerbated by Lebanon's economic crisis as affecting both affordability and quality of antenatal care [26]. Wilson et al. (2024) noted that many countries lack universal healthcare coverage, posing a fundamental challenge to perinatal mental health care access [19].

Zivin et al. (2024) provided the most extensive quantitative evidence on the affordability dimension. Using interrupted time series analysis of US private insurance data (837,316 deliveries), they found that the Mental Health Parity and Addiction Equity Act (MHPAEA) was associated with decreased out-of-pocket costs for psychotherapy, while the Affordable Care Act (ACA) was associated with immediate, sustained increases in psychotherapy use among women with perinatal mood and anxiety disorders (step increase of 0.77%, $P < .001$) [11]. However, the policies also led to fewer visits per person, suggesting that improved access did not necessarily translate into adequate treatment intensity [11]. DeRoche et al. (2023) highlighted high out-of-pocket costs and long wait times for both private and publicly funded therapy in Canada [9].

Appropriateness and Ability to Engage

The appropriateness of services — including whether they were tailored to perinatal populations and culturally diverse groups — was a major concern. Salameh et al. (2024) found that Syrian refugee women experienced intermittent services with limited focus on mental health and no screening for postpartum depression [2]. Berger et al. (2020) identified a lack of guidelines for perinatal mental disorder care as a system-level barrier [4]. Dougherty et al. (2020) found that the most significant barriers in their scoping review occurred in the appropriateness domain, with lack of cultural sensitivity being the most frequently cited [20].

Fisher et al. (2024) emphasized that perinatal competence — the demonstration of perinatal-specific knowledge — was critical for women's engagement, yet many previous services lacked perinatally tailored interventions [14, 14]. Gardner et al. (2024) reported that healthcare professionals described limited opportunities and resources for developing cultural competency in UK perinatal mental health services [25]. Bali et al. (2024) similarly found discrimination and unmet cultural needs as provider-side barriers and lack of trust as a user-side barrier [7].

Recommendations to improve appropriateness centered on training and model redesign. Sambrook Smith et al. (2019) recommended development of a competency framework for staff and provision of culturally sensitive information [13]. Ash et al. (2025) emphasized provider training on trauma-informed, culturally responsive care and increased transparency about screening tools [1]. Webb et al. (2023) called for internationally coordinated efforts to provide woman-centred, flexible care delivered by well-trained and culturally sensitive professionals [6]. Viveiros et al. (2019) recommended that midwives engage in additional perinatal mental health training, maintain awareness of community resources, and initiate discussions using validated screening tools [3].

Barriers Specific to Marginalized Populations

A prominent cross-cutting theme was the disproportionate burden of access barriers on marginalized populations. Refugee women faced compounded barriers at multiple Levesque dimensions: stigma related to both mental illness and refugee status [2], language barriers [2, 27], interpreter confidentiality concerns [27], lack of legal protections [28], and financial precarity [2]. Shah et al. (2024) noted that barriers at individual, sociocultural, organizational, and structural levels contributed to widening perinatal mental health inequalities between refugee and host-country populations [28].

Black perinatal women in the US experienced medical mistrust as a dominant barrier that intersected with approachability, acceptability, and ability to seek care [1]. Spanish-speaking Latinas in the perinatal period perceived benefits from peer-led support (improved access, decreased isolation, reduced stigma) but faced barriers including low awareness and confidentiality concerns, with higher depression and anxiety symptoms associated with more perceived barriers [21]. Collado et al. (2025) found that having Medicaid or being uninsured was linked to more favorable perceptions of peer-delivered support, suggesting that women with fewer alternative resources may be especially receptive to non-traditional service models [21].

Indigenous families in Canada were identified by DeRoche et al. (2023) as marginalized within the existing perinatal mental health system, alongside racialized individuals and those from diverse backgrounds who lacked access to culturally appropriate services [9, 9]. African-born women in Australia faced communication issues, difficulty navigating the health system, and discrimination [7], though positive staff attitudes, continuity of midwifery care, and cultural assimilation served as facilitators [7].

Screening as a Gateway to Access

Multiple studies examined screening as a critical entry point into the care pathway. Kingston et al. (2014) found high public acceptability of universal perinatal mental health screening, with 63.0% of the general population agreeing on prenatal screening and 72.7% on postnatal screening; among women of childbearing age, these figures rose to 79.2% and 88.5%, respectively [15]. Blackmore et al. (2021) developed a seven-stage implementation guide for integrating perinatal mental health screening across health settings, using the iCOPE digital platform to streamline efficient screening, with particular attention to women of refugee background [27, 27]. Baumgartner et al. (2015) described a community-based strategy leveraging church settings in Nigeria for screening pregnant women through an existing prevention of mother-to-child HIV transmission program [18].

However, screening alone was insufficient without clear referral pathways. Viveiros et al. (2019) documented inconsistent screening practices and broken referral pathways as system-level barriers [3]. Blackmore et al. (2021) emphasized the need for a designated referral coordinator and clear referral guidelines to ensure screening led to appropriate follow-up [27]. DeRoche et al. (2023) found that 87% of Canadian providers were not mandated to screen, reflecting a systemic gap [9].

Synthesis

The synthesis across these 31 sources reveals substantial convergence in the identification of barriers, but meaningful variation in the depth and specificity of Levesque framework application, the populations studied, and the types of evidence generated.

The apparent tension between high prevalence of perinatal mental health problems and low treatment rates is explained by barriers operating at every stage of the Levesque access pathway, from perception of need through to engagement with care. The finding that approachability and ability to perceive are the most frequently compromised dimensions [3] is consistent across qualitative studies from diverse settings, including high-income countries with ostensibly well-resourced healthcare systems (UK [13], Canada [9], Netherlands [5], Australia [7]) and populations facing additional structural disadvantage (refugees [2, 27], Black women [1], immigrant women [24]). This convergence suggests that the normalization of perinatal distress and mental health stigma represent near-universal demand-side barriers that are compounded but not caused by marginalization.

However, the nature and relative importance of barriers differ systematically by population context. For refugee and immigrant women, acceptability barriers — including language, cultural mismatch, and discrimination — are particularly prominent [2, 7, 20, 27], whereas for the general perinatal population in high-income settings, organizational barriers such as service fragmentation, long wait times, and workforce shortages appear more salient [8, 9, 13]. For Black perinatal women in the US, medical mistrust represents a distinctive barrier that intersects multiple framework dimensions simultaneously [1]. These population-specific patterns indicate that no single intervention can address access comprehensively; rather, strategies must be tailored to the predominant barriers in specific contexts.

The quantitative evidence from Zivin et al. (2024) demonstrates that policy-level interventions targeting affordability can increase service utilization, but the finding of fewer visits per person after the MHPAEA and ACA [11] suggests

that removing financial barriers alone does not ensure adequate treatment engagement. This aligns with the qualitative evidence that barriers are multilevel and interactive [6, 13]. Similarly, Koire et al. (2024) showed that workforce availability is a structural constraint (21 states with < 1 perinatal psychiatrist per 5,000 births) [8] that cannot be addressed through demand-side interventions alone.

The limited depth of Levesque framework application across studies is notable. Most studies using the framework employed it as a post-hoc coding or interpretive lens rather than as an a priori guide for study design or measurement [1–3]. Only Berger et al. (2020) used the framework to inform both data collection instrument design and analysis [4]. No study operationalized the framework's dimensions with validated measurement scales, and no study tested causal pathways between specific dimensions and access outcomes. This represents a significant gap between the framework's theoretical potential and its empirical application in perinatal mental health research. The framework's interactive, bidirectional conceptualization of supply-side and demand-side factors remains largely untested in this population, with studies typically cataloguing barriers within dimensions rather than examining how dimensions interact to produce access or non-access.

The strongest evidence for facilitators points to integrated care models [1, 12], digital health interventions [10, 27], workforce training in perinatal-specific and culturally responsive competencies [1, 3, 6, 13], and relationship-centered care approaches [14]. However, the evidence base for these facilitators is predominantly descriptive or based on small-scale implementation projects, with limited evaluation of effectiveness in improving measurable access outcomes across the Levesque dimensions.

References

1. Marcia J Ash, B. Woods-Jaeger, Stella Udoetuk, et al (2025) Barriers and Facilitators to Accessing Mental Health Supports Among Black Perinatal Women: Application of the Patient-centered Access Framework. Journal of Racial and Ethnic Health Disparities. https://doi.org/10.1007/s40615-025-02428-3

2. Taghreed N. Salameh, S. Sakarya, C. Acarturk, et al (2024) Syrian refugee women's experiences of barriers to mental health services for postpartum depression. Journal of Advanced Nursing. https://doi.org/10.1111/jan.16407

3. Candice Jacquelyn Viveiros, E. Darling (2019) Perceptions of barriers to accessing perinatal mental health care in midwifery: A scoping review. Midwifery. https://doi.org/10.1016/j.midw.2018.11.011

4. Anke Berger, Karin Schenk, A. Ging, et al (2020) Perinatal mental health care from the user and provider perspective: protocol for a qualitative study in Switzerland. Reproductive Health. https://doi.org/10.1186/s12978-020-0882-7

5. Annemarie de Jong-Bardelmeijer, Janneke T. Gitsels, C.J.M. Verhoeven, Esther I. Feijen-de Jong (2025) Prevalence and Determinants of Health Care Utilization Among Dutch Women in the First Year Postpartum. Journal of midwifery & women's health. https://doi.org/10.1111/jmwh.70055

6. Rebecca Webb, Nazihah Uddin, G. Constantinou, et al (2023) Meta-review of the barriers and facilitators to women accessing perinatal mental healthcare. BMJ Open. https://doi.org/10.1136/bmjopen-2022-066703

7. A. G. Bali, V. Vasilevski, Linda Sweet (2024) Barriers and facilitators of access to maternity care for African-born women living in Australia: a meta-synthesis of qualitative evidence. Systematic Reviews. https://doi.org/10.1186/s13643-024-02628-8

8. A. Koire, Mariella Suleiman, Polina Teslyar, Cindy H Liu (2024) Prevalence of Community Perinatal Psychiatrists in the US. JAMA Network Open. https://doi.org/10.1001/jamanetworkopen.2024.26465

9. Christina DeRoche, Amanda Hooykaas, Christine H K Ou, et al (2023) Examining the gaps in perinatal mental health care: A qualitative study of the perceptions of perinatal service providers in Canada. Frontiers in Global Women's Health. https://doi.org/10.3389/fgwh.2023.1027409

10. A. Lewkowitz, A.R. Whelan, N.K. Ayala, et al (2024) The Effect of Digital Health Interventions on Postpartum Depression or Anxiety: A Systematic Review and Meta-Analysis of Randomized Controlled Trials. Obstetric Anesthesia Digest. https://doi.org/10.1097/01.aoa.0001080184.03651.f7

11. K. Zivin, Xiaosong Zhang, A. Tilea, et al (2024) Perinatal Psychotherapy Use and Costs Before and After Federally Mandated Health Insurance Coverage. JAMA Network Open. https://doi.org/10.1001/jamanetworkopen.2024.26802

12. Elizabeth Q. Cox, C. Raines, M. Kimmel, et al (2017) Comprehensive Integrated Care Model to Improve Maternal Mental Health. Journal of Obstetric, Gynecologic and Neonatal Nursing. https://doi.org/10.1016/j.jogn.2017.08.003

13. Megan Rose Sambrook Smith, V. Lawrence, E. Sadler, A. Easter (2019) Barriers to accessing mental health services for women with perinatal mental illness: systematic review and meta-synthesis of qualitative studies in the UK. BMJ Open. https://doi.org/10.1136/bmjopen-2018-024803

14. L. Fisher, A. Davey, G. Wong, et al (2024) Women’s engagement with community perinatal mental health services: a realist evaluation. BMC Psychiatry. https://doi.org/10.1186/s12888-024-05804-1

15. D. Kingston, S. McDonald, S. Tough, et al (2014) Public views of acceptability of perinatal mental health screening and treatment preference: a population based survey. BMC Pregnancy and Childbirth. https://doi.org/10.1186/1471-2393-14-67

16. J. O’Mahony, Shahin Kassam, L. Marcellus (2021) Facilitators and barriers to accessing mental health care services and social supports among perinatal refugee women: a qualitative systematic review protocol. JBI Evidence Synthesis. https://doi.org/10.11124/JBIES-21-00119

17. H. A. Rahim (2018) Barriers to accessing mental health services for women with perinatal mental illness: systematic review and meta-synthesis of qualitative studies in the UK

18. J. Baumgartner, S. Kaaya, H. Siril (2015) Mental health screenings for couples at churches in Nigeria: a strategy for enhancing community-based maternal mental health services in low-resource settings. Social Psychiatry and Psychiatric Epidemiology. https://doi.org/10.1007/s00127-014-0988-8

19. Claire A Wilson, Margaret Bublitz, Prabha Chandra, et al (2024) A global perspective: Access to mental health care for perinatal populations. Seminars in Perinatology. https://doi.org/10.1016/j.semperi.2024.151942

20. Louise Dougherty, J. Lloyd, E. Harris, et al (2020) Access to appropriate health care for non-English speaking migrant families with a newborn/young child: a systematic scoping literature review. BMC Health Services Research. https://doi.org/10.1186/s12913-020-05157-x

21. Anahi Collado, Laurel Hicks, Samuel Hubley, et al (2025) Perceptions of Peer-Delivered Mental Health Support for Anxiety and Depression Among Spanish-Speaking, Perinatal Latinas in the United States. Journal of health care for the poor and underserved. https://doi.org/10.1353/hpu.2025.a959117

22. Kerstin Weidner, Michaela Galle, A. Bittner, et al (2017) [Psychotherapeutic care of women in pregnancy and peripartum: Evaluation and treatment utilization]. Zeitschrift für psychosomatische Medizin und Psychotherapie. https://doi.org/10.13109/zptm.2017.63.2.176

23. Keiichiro Narumoto, Miho Endo, M. Kaneko, et al (2024) Japanese primary care physicians' postpartum mental health care: A cross-sectional study. Journal of general and family medicine. https://doi.org/10.1002/jgf2.700

24. J. O'Mahony, N. Clark (2018) Immigrant Women and Mental Health Care: Findings from an Environmental Scan. Issues in Mental Health Nursing. https://doi.org/10.1080/01612840.2018.1479903

25. Angelene Gardner, S. Oduola, Bonnie Teague (2024) Culturally Sensitive Perinatal Mental Health Care: Experiences of Women From Minority Ethnic Groups. Health Expectations. https://doi.org/10.1111/hex.14160

26. Carla El Alam, Linda Abou-Abbas, Mohamad Shafic Ramadan, M. K. Asmar (2025) Exploring the barriers to accessing antenatal care at the primary health care center level of a tertiary hospital in Lebanon: a qualitative study. BMC Health Services Research. https://doi.org/10.1186/s12913-025-12444-y

27. R. Blackmore, J. Boyle, K. Gray, et al (2021) Introducing and integrating perinatal mental health screening: Development of an equity-informed evidence-based approach. Health Expectations. https://doi.org/10.1111/hex.13526

28. Kalina Shah, Emilie Janssen (2024) BARRIERS FACED BY REFUGEE WOMEN AND BIRTHING PEOPLE ACCESSING UK PERINATAL MENTAL HEALTH SERVICES. The Student Midwife. https://doi.org/10.55975/pqny1935

29. Kristin Pullyblank, M. Scribani, N. Krupa, et al (2023) Exploring Multiple Dimensions of Access to and Preferences for Telehealth Use. Telemedicine Reports. https://doi.org/10.1089/tmr.2023.0049

30. Zoi Triandafilidis, Breanne Hobden, Shannon Richardson, Mariko Carey (2025) "They can build up trust again, so that health is not such a scary place": The acceptability and feasibility of a multidisciplinary primary care outreach service for women affected by domestic, family, and sexual violence. Australian Journal of Primary Health. https://doi.org/10.1071/PY25040

31. Wanlu Xu (2021) Patient Perspectives on Barriers and Facilitators to Mental Health Support after a Traumatic Birth. https://doi.org/10.13028/4QFY-AC94
