# Supplementary material for: Patient-centered access to mental health care at selected life-course stages: a scoping review using the Levesque framework
Source: BMC Health Serv Res. 2026 Oct 22;26:1464. doi: 10.1186/s12913-026-15895-z (PMC13693167; doi:10.1186/s12913-026-15895-z)
Supplement: Supplementary file 3 [file 12913_2026_15895_MOESM3_ESM.pdf]

Which empirical studies and evidence syntheses published from 2013 onward have substantively applied the Levesque et al. (2013) patient-centred access to health care framework to access to, utilization of, or engagement with mental health services, mental-health diagnostic pathways, or treatment pathways among children and adolescents aged 0–17 years?

Three sources substantively applied the Levesque framework to child and adolescent mental health access: a systematic review of RCTs on access-improving interventions (Werlen et al., 2019), an integrative review of barriers among youth refugees and asylum seekers (Barbo & Petrucka, 2024), and a retrospective cohort study of ADHD diagnostic pathways in Mexico City (Martínez-Jaime et al., 2024), collectively indicating that the framework has seen limited but growing application in this population, with existing evidence concentrated on the approachability and appropriateness dimensions while structural dimensions such as affordability and availability remain largely unaddressed experimentally.

Abstract

Three sources published from 2013 onward substantively applied the Levesque et al. (2013) patient-centred access framework to children's mental health access: a systematic review and meta-analysis of 34 RCTs of access-improving interventions for youth under 19 in high-income countries [1], an integrative review of 29 studies on barriers among youth refugees and asylum seekers [2], and a retrospective cohort study of 177 children's ADHD diagnostic pathways at a tertiary hospital in Mexico City [3]. All three used the framework to structure study design, coding, and interpretation rather than citing it in passing [1–3]. The evidence base is small and heterogeneous in design, population, and setting, but converges on several points. Access barriers are multi-dimensional and sequential: school-based programs reliably improved mental health knowledge and attitudes but only 20% improved actual service utilization [1], consistent with the finding that the longest diagnostic delay for ADHD occurred between symptom perception and help-seeking rather than within the health system [3]. Targeted engagement interventions—appointment reminders (OR 3.11) and motivational strategies (OR 3.51)—approximately tripled first-appointment attendance among at-risk youth [1]. Demographic disparities were prominent where measured: girls with ADHD were diagnosed approximately one year later than boys (AHR 0.57) [3], and compounding barriers across all five framework dimensions produced widespread underutilization among refugee youth [2]. Existing RCT evidence addresses only two of the five Levesque dimensions—approachability and appropriateness—while acceptability, affordability, and ability to reach or pay remain untested experimentally in child populations [1], despite qualitative evidence confirming their relevance [2, 3].

Flow Diagram

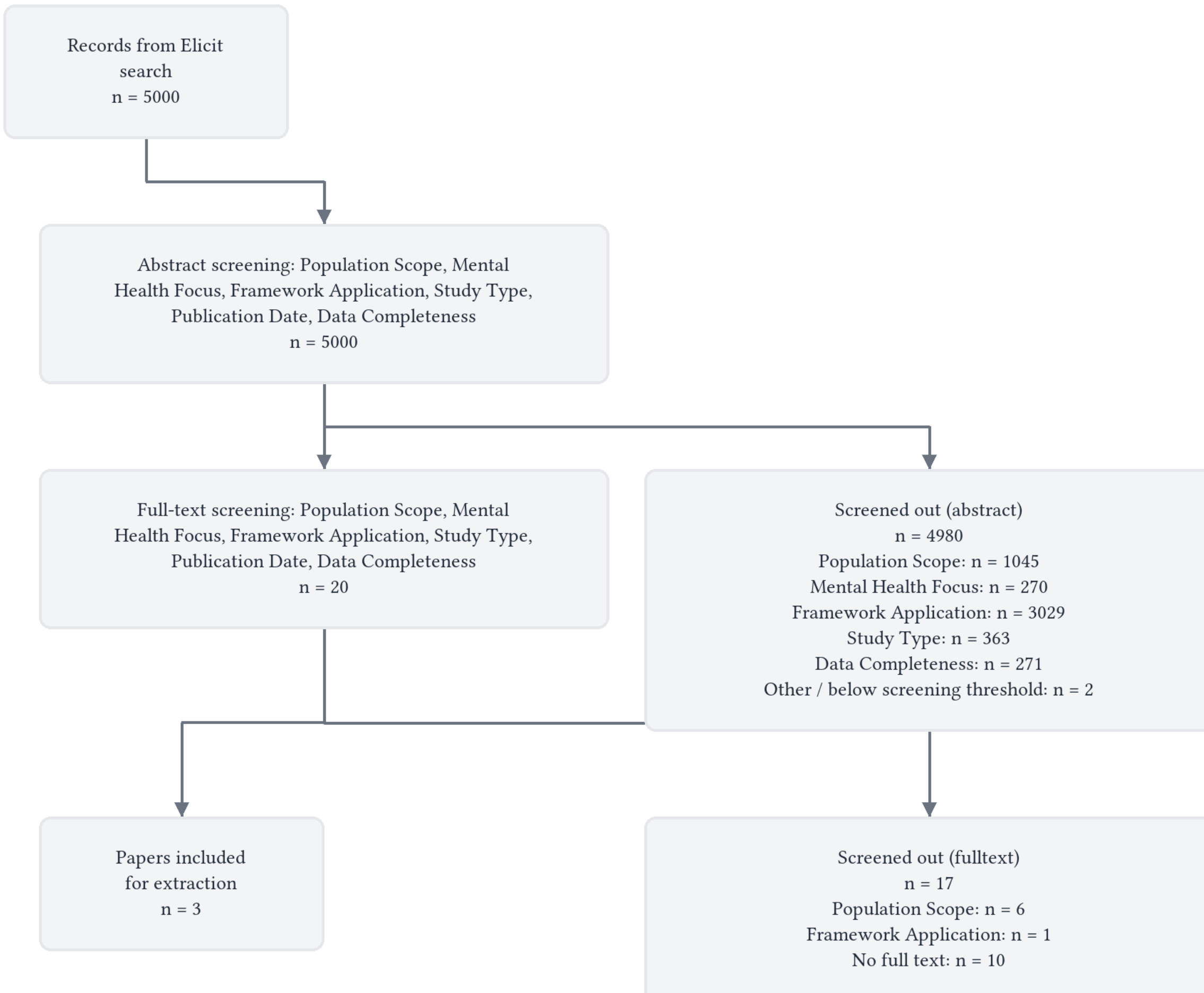

Paper search

We performed a semantic search across over 138 million academic papers from the Elicit search engine, which includes all of Semantic Scholar and OpenAlex.

We ran this query: "Which empirical studies and evidence syntheses published from 2013 onward have substantively applied the Levesque et al. (2013) patient-centred access to health care framework to access to, utilization of, or engagement with mental health services, mental-health diagnostic pathways, or treatment pathways among children and adolescents aged 0–17 years?"

The search returned 5,000 total results from Elicit.

We retrieved 5000 papers most relevant to the query for screening.

Screening

Abstract screening

We screened in sources based on their abstracts that met these criteria:

- **Population Scope**: Does the study focus on children/adolescents aged 0-17 years, OR does it include mixed-age populations with separately reported findings for the 0-17 age group?
- **Mental Health Focus**: Is access to, utilization of, or engagement with mental health services, mental health diagnostic pathways, or treatment pathways a primary or substantial focus of the study?
- **Framework Application**: Does the study explicitly and substantively apply the Levesque et al. (2013) patient-centred access framework to inform study design, research questions, data collection, measurement, analysis, coding, or interpretation (beyond merely citing it)?
- **Study Type**: Is this a completed primary empirical study (qualitative, quantitative, or mixed-methods) OR an evidence synthesis (systematic review, scoping review, or mapping review)?
- **Publication Date**: Was the study published in 2013 or later?
- **Data Completeness**: Is this a completed study with sufficient data (i.e., NOT an ongoing study, conference abstract without full publication, or study with incomplete data)?

Papers that failed any strict criterion were automatically excluded. For the remaining papers, we considered all screening questions together and made a holistic judgement about whether to screen in each paper.

20 papers passed abstract screening and moved to full-text screening.

At abstract screening, the number of papers excluded for each primary reason was:

- **Population Scope**: n = 1045
- **Mental Health Focus**: n = 270
- **Framework Application**: n = 3029
- **Study Type**: n = 363
- **Data Completeness**: n = 271
- **Other / below screening threshold**: n = 2

Full-text screening

We then screened papers based on their full text using these additional criteria:

- **Population Scope**: Does the study focus on children/adolescents aged 0-17 years, OR does it include mixed-age populations with separately reported findings for the 0-17 age group?
- **Mental Health Focus**: Is access to, utilization of, or engagement with mental health services, mental health diagnostic pathways, or treatment pathways a primary or substantial focus of the study?
- **Framework Application**: Does the study explicitly and substantively apply the Levesque et al. (2013) patient-centred access framework to inform study design, research questions, data collection, measurement, analysis, coding, or interpretation (beyond merely citing it)?
- **Study Type**: Is this a completed primary empirical study (qualitative, quantitative, or mixed-methods) OR an evidence synthesis (systematic review, scoping review, or mapping review)?
- **Publication Date**: Was the study published in 2013 or later?
- **Data Completeness**: Is this a completed study with sufficient data (i.e., NOT an ongoing study, conference abstract without full publication, or study with incomplete data)?

Papers that failed any strict criterion were automatically excluded. For the remaining papers, we considered all screening questions together and made a holistic judgement about whether to include each paper in the final analysis.

3 papers passed full-text screening and moved to data extraction.

At full-text screening, the number of papers excluded for each primary reason was:

- **Population Scope**: n = 6
- **Framework Application**: n = 1
- **No full text**: n = 10

Data extraction

We asked a large language model to extract each data column below from each paper. We gave the model the extraction instructions shown below for each column.

- **Framework Application**:

  Extract detailed information about how the Levesque et al. (2013) patient-centred access framework was substantively applied in this study, including:

- Specific ways the framework informed study design, research questions, data collection, measurement, analysis, coding, or interpretation
- Whether framework dimensions were used as organizing themes, coding categories, measurement domains, or analytical structure
- How the framework went beyond a passing citation to actually shape the research approach
- Any adaptations or modifications made to the framework for child/adolescent populations

- **Population Characteristics**:

  Extract details about the child and adolescent population studied (aged 0-17 years), including:

- Age range and mean age
- Sample size for participants aged 0-17 years
- Demographic characteristics (gender, ethnicity, socioeconomic status)
- Clinical characteristics (mental health conditions, severity)
- Whether findings for 0-17 year olds were reported separately from other age groups
- Geographic location and setting (urban/rural, country/region)

- **Mental Health Focus**:

  Extract information about the specific mental health services, diagnostic pathways, or treatment pathways that were the focus regarding access, utilization, or engagement, including:

- Type of mental health services (e.g., specialized services, primary care, school-based, inpatient, outpatient)
- Specific mental health conditions addressed (e.g., ADHD, depression, anxiety, general mental health)
- Whether focus was on diagnostic pathways, treatment pathways, or general service access
- Care settings and service delivery models examined

- **Study Design**:

  Extract the study design and methodology, specifying:

- Type of empirical study (qualitative, quantitative, mixed-methods primary study) or evidence synthesis (systematic review, scoping review, mapping review)
- Specific research design (e.g., cross-sectional survey, cohort study, RCT, thematic synthesis)
- Data collection methods used
- Sample size and sampling approach
- Time period of data collection

- **Levesque Framework Dimensions**:

  Extract which specific dimensions of the Levesque framework were addressed or applied in this study, including:

- Approachability (and ability to perceive)
- Acceptability (and ability to seek)
- Availability and accommodation (and ability to reach)
- Affordability (and ability to pay)
- Appropriateness (and ability to engage)
- Whether all five dimensions were addressed or only specific ones
- How each dimension was operationalized or measured in the child/adolescent context

- **Access Barriers**:

  Extract findings about barriers to access, utilization, or engagement with mental health services among children and adolescents aged 0-17 years, including:

- Specific barriers identified within each Levesque framework dimension
- Barriers unique to child/adolescent populations
- Systemic, provider, or individual-level barriers
- How barriers differed by age, gender, condition, or other characteristics within the 0-17 age range

- **Access Facilitators**:

  Extract findings about facilitators or enablers of access, utilization, or engagement with mental health services among children and adolescents aged 0-17 years, including:

- Specific facilitators identified within each Levesque framework dimension
- Facilitators unique to child/adolescent populations
- Systemic, provider, or individual-level facilitators
- How facilitators differed by age, gender, condition, or other characteristics within the 0-17 age range

- **Key Access Findings**:

  Extract the main findings related to access, utilization, or engagement with mental health services among children and adolescents aged 0-17 years, including:

- Quantitative results (rates, odds ratios, effect sizes) related to access or utilization
- Qualitative themes about access experiences
- Time to access or diagnosis for different pathways
- Disparities in access by demographic characteristics
- Service utilization patterns and engagement rates
- How findings specifically relate to the child/adolescent population

Results

Characteristics of Included Studies

Three sources met the inclusion criteria for this review: two evidence syntheses and one primary empirical study. All three substantively applied the Levesque et al. (2013) patient-centred access framework beyond a passing citation, using it to structure study design, data collection or coding, analysis, and interpretation. The table below summarizes key characteristics.

Characteristic	Werlen et al. (2019)	Barbo & Petrucka (2024)	Martínez-Jaime et al. (2024)
Full text retrieved?	Yes	Yes	Yes
Study type	Systematic review and meta-analysis of RCTs [1]	Integrative literature review [2]	Retrospective cohort study (primary quantitative study) [3]
Population	Children and adolescents <19 years (>50% under 19 if broader age range) [1]	Youth refugees and asylum seekers; flexible definition of "youth," excluding prepubescent children and adults 18+ [2]	177 dyads (child/adolescent with ADHD and primary caregiver), all aged 0–17 [3]
Mental health focus	General mental health, depression, suicidality, substance use, behavioral/conduct problems, ADHD [1]	General mental health including PTSD, depression, anxiety, behavioral problems [2]	ADHD exclusively [3]

Characteristic	Werlen et al. (2019)	Barbo & Petrucka (2024)	Martínez-Jaime et al. (2024)
Service/pathway focus	Access and engagement across the care pathway: school-based programs (earlier steps) and health-care-based engagement interventions (later steps) [1]	General service access and engagement barriers across prevention, screening, evaluation, treatment, and monitoring [2]	Diagnostic access pathway from perception of symptoms to formal ADHD diagnosis at a specialized hospital [3]
Geographic scope	34 RCTs in high-income countries: USA (22), Australia (5), UK (3), Canada (2), Portugal (1), Israel (1); none from LMICs [1]	Host countries: Australia (10), UK (8), USA (4), Canada (3), Belgium, Germany, Netherlands, Sweden (1 each) [2]	Mexico City, Mexico; single urban tertiary children's psychiatric hospital [3]
Included studies/sample	34 RCTs (from 5641 screened records) [1]	29 studies included [2]	177 children/adolescents; 81.4% boys, 18.6% girls; mean age at diagnosis 8.2 years [3]
Levesque dimensions addressed	Primarily approachability/ability to perceive and appropriateness/ability to engage; availability minimally; acceptability, affordability, ability to reach/pay not addressed by any included RCTs [1]	All five dimensions addressed [2]	All five dimensions addressed through the Access Pathway stages and AAcCeDa survey [3]
Framework application	Defined outcomes and mapped each RCT to framework dimensions; figures organized by framework boxes; meta-analyses aligned to utilization step [1]	Deductive coding scheme using framework dimensions as predefined categories; five themes mirror the five dimension–ability pairs [2]	Constructed a five-stage Access Pathway mirroring framework steps; developed and validated the AAcCeDa survey from framework dimensions; measured ages and intervals at each stage [3]
Search/data collection period	Literature searched up to 15 May 2019 [1]	Search completed 22 March 2022 [2]	Data collection period not reported; manuscript accepted April 2024 [3]

The three sources differ substantially in design, population, and scope but converge on their use of the Levesque framework as an organizing and analytical structure for child and adolescent mental health access. Werlen et al. (2019) is the broadest in scope, synthesizing 34 RCTs of interventions to improve access across multiple mental health conditions and high-income country settings [1]. Barbo and Petrucka (2024) offers a qualitative-thematic synthesis of barriers among a specific vulnerable subpopulation—youth refugees and asylum seekers—across 29 studies [2]. Martínez-Jaime et al. (2024) is the only primary empirical study, providing granular, quantitative data on diagnostic delays for ADHD within a single Mexican institution [3]. Notably, two of the three sources are evidence syntheses (reviews), while only one contributes direct empirical evidence.

Barriers to Access

All three sources identified barriers across multiple Levesque framework dimensions, though the depth and specificity of barrier identification varied by study design and population.

Levesque Dimension	Werlen et al. (2019)	Barbo & Petrucka (2024)	Martínez-Jaime et al. (2024)
Approachability / Ability to perceive	Low mental health literacy and stigma among youth; insufficient outreach/screening [1]	Low awareness of services; negative or binary conceptualizations of mental illness; cultural/religious beliefs (e.g., possession) [2]	Caregivers do not recognize ADHD core symptoms; teachers underidentify girls' internalizing symptoms; limited community mental health literacy [3]
Acceptability / Ability to seek	Cultural and gendered norms reducing willingness to seek care; limited adolescent autonomy [1]	Social, institutional, and self-stigma; cultural mismatch; racism/xenophobia; confidentiality concerns especially with interpreters [2]	Latino parents less likely to seek or continue care; single-mother burden; more siblings associated with delays (AHR 0.63) [3]
Availability and accommodation / Ability to reach	Inconvenient locations and hours; burdensome appointment mechanisms [1]	Strict eligibility criteria; service age cutoffs; rules penalizing missed appointments; long waiting lists (months); hours conflicting with school; distant locations; complex referrals; fragmented system [2]	Only one tertiary psychiatric hospital; long waits (95% reported); no referral/counter-referral system; fragmentation between care levels; limited UDEEI school staffing [3]
Affordability / Ability to pay	Out-of-pocket costs; lack of insurance or assets [1]	Financial burden amid competing resettlement priorities (housing, food, employment) [2]	Informal/no employment of caregiver delays diagnosis; formal employment improves timeliness (AHR 1.45) [3]
Appropriateness / Ability to engage	Insufficient provider training/confidence; inadequate reimbursement; limited coordination [1]	Talking therapies seen as culturally inappropriate and retraumatizing; interrogative provider style; low provider training (~25% received refugee-specific training; ~15% felt competent); language barriers [2]	Misdiagnosis/underidentification in girls; psychologists retaining cases without referral to specialists; comorbidities complicating screening [3]

A convergent finding across all three sources is that barriers at the earliest framework stages—approachability and ability to perceive—are fundamental constraints on children's access. Low mental health literacy among both youth and their caregivers was identified as a barrier in all three studies [1–3]. Stigma, whether framed as social attitudes in the general population [1], multi-layered stigma among refugee communities [2], or cultural beliefs among Latino families [3], consistently impeded help-seeking. System-level availability barriers—including fragmented referral pathways, long waiting times, and geographic distance—were prominent in both the refugee-focused review [2] and the Mexico City cohort study [3], while the RCT review noted that virtually no trials had targeted these structural dimensions [1].

Barriers specific to children and adolescents merit particular attention. Dependence on caregivers for recognition, transport, and engagement was highlighted across sources [1, 3]. Among refugee youth, school-hour conflicts with clinic schedules, age cutoffs for services, and parental dynamics (minimizing symptoms or punishing disclosure) created child-specific obstacles [2]. In the ADHD cohort, teacher detection bias toward externalizing behaviors

systematically disadvantaged girls, who were perceived approximately one year later at the initial pathway stage and experienced a total diagnostic delay roughly 0.8 years longer than boys [3].

Facilitators of Access

Levesque Dimension	Werlen et al. (2019)	Barbo & Petrucka (2024)	Martínez-Jaime et al. (2024)
Approachability / Ability to perceive	School-based education, contact-based programs, and screenings improved knowledge and attitudes [1]	Proposed: enhance mental health literacy through schools/communities; frame content as wellbeing [2]	Teachers were the only identified facilitator of recognition; teachers prompt caregivers to seek help [3]
Acceptability / Ability to seek	Not directly tested in included RCTs [1]	Proposed: co-design with refugee youth and communities to ensure cultural fit; stigma reduction [2]	Not specifically identified beyond teacher prompting [3]
Availability and accommodation / Ability to reach	Appointment reminders (phone/letter) increased first attendance (OR 3.11) [1]; onsite mental health in primary care identified as promising [1]	Not specifically identified [2]	Proposed: school-based service models, referral/counter-referral systems, links between schools and primary care [3]
Affordability / Ability to pay	Not tested in included RCTs [1]	Proposed: policy mechanisms such as Mental Health Parity Act [2]	Caregiver formal employment improved diagnostic timeliness (AHR 1.45) [3]
Appropriateness / Ability to engage	Treatment engagement/motivational strategies increased first attendance (OR 3.51); caregiver-directed components for younger children [1]	Proposed: provider cultural humility training; treating youth as partners; adapted communication [2]	Proposed: psychoeducational workshops; training first-level providers for ADHD management and referral [3]

The strongest quantitative evidence for facilitators comes from the meta-analyses in Werlen et al. (2019), which demonstrated that relatively simple interventions—appointment reminders (OR 3.11, 95% CI 2.07–4.67) and motivational/engagement strategies (OR 3.51, 95% CI 2.02–6.11)—approximately tripled the odds of initial appointment attendance among at-risk children and adolescents [1]. Schools emerged as a critical setting across all sources: school-based literacy programs improved knowledge and attitudes [1], and teachers were the only empirically confirmed facilitator in the ADHD diagnostic pathway [3]. However, school-based programs only rarely translated into actual service utilization (20% of universal school-based interventions improved access) [1], suggesting that knowledge gains alone are insufficient without systematic referral mechanisms.

Key Quantitative Access Findings

The following table consolidates the principal quantitative findings on access, utilization, and disparities reported across the sources.

Finding	Source	Value
Pooled OR for first appointment attendance with reminders	Werlen et al. (2019)	3.11 (95% CI 2.07–4.67) [1]

Finding	Source	Value
Pooled OR for first attendance with engagement interventions	Werlen et al. (2019)	3.51 (95% CI 2.02–6.11; $I^2 \approx 70\%$) [1]
Proportion of at-risk interventions improving access	Werlen et al. (2019)	71% (15/21) [1]
Proportion of universal school interventions improving access	Werlen et al. (2019)	20% (1/5) [1]
Proportion of at-risk interventions improving mental health outcomes	Werlen et al. (2019)	33% (2/6) [1]
Proportion with late or worse ADHD diagnosis	Martínez-Jaime et al. (2024)	71% [3]
Proportion with optimal ADHD diagnosis (≤4.5 years)	Martínez-Jaime et al. (2024)	0.6% [3]
Median age at ADHD diagnosis, boys	Martínez-Jaime et al. (2024)	7.33 years [3]
Median age at ADHD diagnosis, girls	Martínez-Jaime et al. (2024)	8.33 years [3]
Median total perception-to-diagnosis delay, boys	Martínez-Jaime et al. (2024)	~1.75 years [3]
Median total perception-to-diagnosis delay, girls	Martínez-Jaime et al. (2024)	~2.54 years [3]
AHR for timely diagnosis in girls vs boys	Martínez-Jaime et al. (2024)	0.57 (95% CI 0.37–0.86) [3]
AHR for timely diagnosis with caregiver formal employment	Martínez-Jaime et al. (2024)	1.45 (95% CI 1.00–2.11) [3]
AHR for timely diagnosis with additional siblings	Martínez-Jaime et al. (2024)	0.63 (95% CI 0.44–0.91) [3]

Two patterns stand out. First, the distinction between improving awareness versus improving actual utilization is striking: universal school programs reliably increased mental health knowledge (80% of studies measuring knowledge showed improvement) and attitudes (67%), yet only 20% improved actual care access, and none improved mental health outcomes [1]. In contrast, targeted interventions for at-risk youth achieved substantially higher rates of improved access (71%) and satisfaction (80%), though even these improved mental health outcomes in only a third of studies [1]. This pattern aligns with the Levesque framework's sequential logic—intervening at the perception stage alone does not ensure progression through seeking, reaching, and utilizing services.

Second, demographic disparities in access are pronounced where measured. In the ADHD cohort, girls were diagnosed approximately one year later than boys (median 8.33 vs. 7.33 years), with their adjusted hazard ratio for timely diagnosis nearly halved (AHR 0.57) [3]. Socioeconomic factors also modulated access: children with more siblings faced longer delays (AHR 0.63), while caregiver formal employment was associated with more timely diagnosis (AHR 1.45) [3]. Among refugee youth, compounding barriers across all five framework dimensions—stigma, system fragmentation, financial constraints, cultural mismatch, and language barriers—produced widespread underutilization of formal services despite high need [2].

Framework Coverage and Adaptations

A notable finding across the three sources concerns the uneven coverage of Levesque framework dimensions in the existing evidence base. Werlen et al. (2019) documented that no included RCTs addressed acceptability, affordability, or youth's ability to reach or pay for services [1], indicating that the experimental literature on children's mental health access is concentrated on approachability and appropriateness while neglecting structural and financial dimensions. Barbo and Petrucka (2024), by contrast, identified substantial barriers across all five dimensions in their qualitative synthesis [2], suggesting that these under-studied dimensions are indeed relevant to children's access experiences. Martínez-Jaime et al. (2024) operationalized all five dimensions through their staged Access Pathway

and survey instrument, although affordability was captured indirectly via socioeconomic proxies rather than direct cost measurement [3].

Each study adapted the Levesque framework to the child/adolescent context differently. Werlen et al. (2019) tailored outcome operationalizations for youth mental health—adding caregiver support under "ability to engage" and targeting younger children through parent-directed interventions versus adolescent-directed interventions [1]. Barbo and Petrucka (2024) applied the framework without stated modification to youth refugees and asylum seekers [2]. Martínez-Jaime et al. (2024) made the most extensive adaptations: anchoring pathway stages to child age, defining ADHD-specific optimal diagnosis thresholds, centering the dyad (child–caregiver) as the unit of analysis, foregrounding the school/teacher as a facilitator, and developing a disorder-specific survey instrument derived from framework dimensions [3].

Synthesis

The three sources present consistent rather than contradictory findings, though they illuminate different facets of children's mental health access. The central convergence is that access barriers are multi-dimensional and sequential: addressing a single Levesque dimension—such as improving knowledge through school-based programs—does not reliably translate into service utilization unless subsequent dimensions (seeking, reaching, engaging) are also addressed [1]. This is reinforced by the ADHD diagnostic pathway data, where the longest delay occurred between perception and search (approximately one year) [3], and by the refugee youth findings showing that even when youth are aware of services, stigma, system rules, and cultural mismatch prevent engagement [2].

The apparent gap between school-based awareness gains and actual utilization improvements in Werlen et al. (2019) and the teacher-as-sole-facilitator finding in Martínez-Jaime et al. (2024) both point toward the same conclusion: schools are effective platforms for the perception stage of the access pathway but require integration with systematic referral and engagement mechanisms to move children through subsequent stages [1, 3]. The one universal school-based intervention that improved actual service access in the RCT review was a screening-plus-referral program, not a literacy-only intervention [1].

The populations and settings across the three sources differ considerably—high-income country general populations [1], refugee youth in host countries [2], and urban Mexican children with ADHD at a single tertiary center [3] —yet the framework dimensions where barriers concentrate are remarkably consistent. This consistency suggests that the Levesque framework captures genuinely generalizable access constructs for child and adolescent mental health, while the specific manifestation of barriers within each dimension varies by context (e.g., cultural stigma in refugee communities versus diagnostic gender bias in ADHD populations).

The evidence base applying the Levesque framework to child and adolescent mental health access remains small: only three sources were identified, of which only one is a primary empirical study. Experimental evidence (RCTs) is concentrated in high-income, English-speaking countries and addresses only two of the five framework dimensions [1]. The sole primary study from a low- or middle-income country provides detailed diagnostic pathway data but is limited to a single condition and institution [3]. Qualitative evidence on barrier experiences is richer across all dimensions but lacks quantification of effect sizes for facilitating interventions [2]. A randomized trial testing a multi-component intervention that simultaneously targets at least three Levesque dimensions (e.g., school-based screening, referral facilitation, and affordability supports) in a low- or middle-income setting would address the most prominent gaps in this evidence base.

References

1. Werlen L, Werlen L, Gjukaj D, et al (2019) Interventions to improve children's access to mental health care: a systematic review and meta-analysis. Epidemiology and Psychiatric Sciences. https://doi.org/10.1017/S2045796019000544

2. Barbo G, Petrucka P (2024) BARRIERS ASSOCIATED WITH INACCESSIBLE MENTAL HEALTH SERVICES FOR YOUTH REFUGEES AND ASYLUM SEEKERS: AN INTEGRATIVE REVIEW. International Journal of Child, Youth & Family Studies. https://doi.org/10.18357/ijcyfs144202421719

3. Martínez-Jaime MM, Reyes-Morales H, Peyrot-Negrete I, Barrientos-Álvarez MS (2024) Access to early diagnosis for attention-deficit/hyperactivity disorder among children and adolescents in Mexico City at specialized mental health services. BMC Health Services Research. https://doi.org/10.1186/s12913-024-11022-y
