# Supplementary material for: Patient-centered access to mental health care at selected life-course stages: a scoping review using the Levesque framework
Source: BMC Health Serv Res. 2026 Oct 22;26:1464. doi: 10.1186/s12913-026-15895-z (PMC13693167; doi:10.1186/s12913-026-15895-z)
Supplement: Supplementary file 4 [file 12913_2026_15895_MOESM4_ESM.pdf]

Find studies that cite or apply the Levesque et al. (2013) patient-centred access to health care framework in the context of mental health services. Include conceptual, empirical, and implementation studies (2013–present). Provide details on how the framework is used and which access dimensions are operationalized.

The Levesque framework has been applied in mental health contexts both as a direct empirical tool—operationalizing demand-side abilities such as ability to perceive, seek, reach, and engage into measurable diagnostic pathway milestones for childhood ADHD—and as a supplementary conceptual reference alongside the social-ecological model for understanding multilevel barriers to adolescent mental health service access, though supply-side dimensions such as Affordability and Appropriateness remain largely unoperationalized in this domain.

Abstract

Two studies applying or citing the Levesque et al. (2013) patient-centred access framework in mental health contexts were identified, spanning empirical and conceptual approaches. Martínez-Jaime et al. (2024) used the framework as its primary theoretical basis to develop a novel survey instrument (the AAcceDa survey) and a five-stage "Access Pathway" measuring the temporal journey to ADHD diagnosis among children and adolescents in Mexico City [1]. This study operationalized four demand-side abilities—ability to perceive, seek, reach, and engage—as discrete age-based milestones, finding that 71% of dyads experienced late diagnosis, with girls diagnosed approximately one year later than boys [1, 1]. Harper et al. (2018) cited the Levesque framework as one of several conceptual models, foregrounding the social-ecological model to discuss multilevel barriers to adolescent and young adult healthcare access in the United States, including confidentiality concerns and declining provider visits from 86% in childhood to 56% in young adulthood [2, 2].

Both studies converge on schools as a critical setting for improving mental health service access [1, 2] and on the importance of relationship-level factors such as caregiver and parental involvement [1, 2]. However, the framework's supply-side dimensions (Approachability, Affordability, Appropriateness) remain largely unoperationalized across both studies [1, 2], indicating that current applications in mental health contexts have primarily leveraged the demand-side of the framework. The empirical operationalization demonstrated by Martínez-Jaime et al. (2024) illustrates that the Levesque framework can be effectively translated into measurable access indicators for mental health services [1], though comprehensive application of all framework dimensions in this domain has yet to be realized.

Flow Diagram

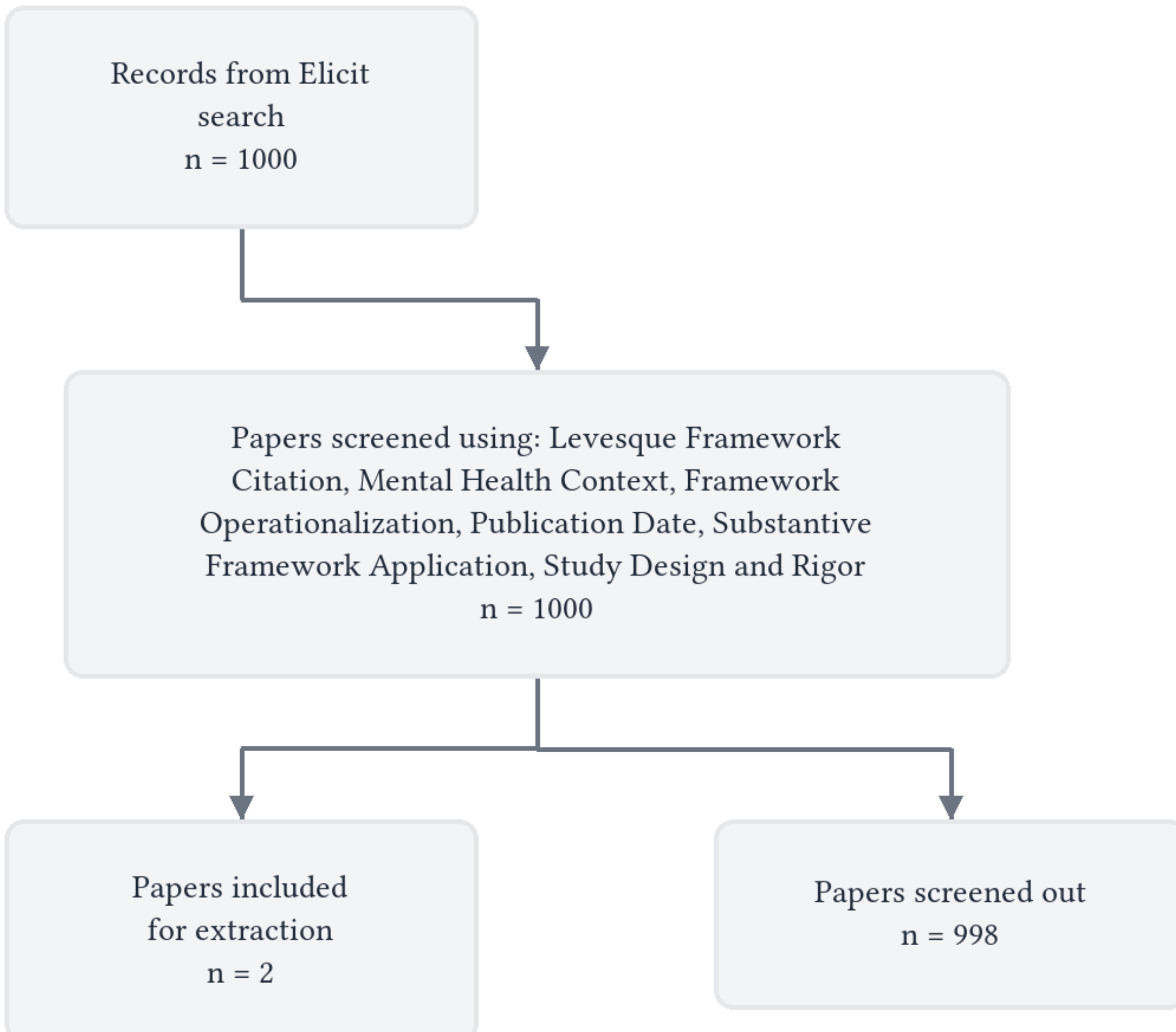

Paper search

We performed a semantic search across over 138 million academic papers from the Elicit search engine, which includes all of Semantic Scholar and OpenAlex.

We ran this query: "Find studies that cite or apply the Levesque et al. (2013) patient-centred access to health care framework in the context of mental health services. Include conceptual, empirical, and implementation studies (2013–present). Provide details on how the framework is used and which access dimensions are operationalized. Are there studies applying the Levesque et al. (2013) access to healthcare framework to mental health services for

children, adolescents, perinatal women, or older adults? Include empirical or conceptual studies (2013–present).”

We applied the following filters: published after 2013; including keywords: children, adolescents.

The search returned 1,000 total results from Elicit.

We retrieved 1000 papers most relevant to the query for screening.

Screening

We screened in sources based on their abstracts that met these criteria:

- **Levesque Framework Citation**: Does this study explicitly cite, reference, or apply the Levesque et al. (2013) patient-centred access to healthcare framework?
- **Mental Health Context**: Is this study conducted within mental health services, programs, or populations?
- **Framework Operationalization**: Does this study provide information about how the Levesque framework is used or which access dimensions are operationalized?
- **Publication Date**: Was this study published in 2013 or later?
- **Substantive Framework Application**: Does this study demonstrate meaningful engagement with the Levesque framework beyond mere passing reference (i.e., includes substantive application, analysis, or discussion of its components)?
- **Study Design and Rigor**: Is this study a conceptual, empirical, or implementation study with appropriate methodological rigor or significant conceptual contribution (e.g., theoretical papers, quantitative/qualitative research, mixed-methods studies, case studies, program evaluations, systematic reviews, meta-analyses)?

Papers that failed any strict criterion were automatically excluded. For the remaining papers, we considered all screening questions together and made a holistic judgement about whether to screen in each paper.

Data extraction

We asked a large language model to extract each data column below from each paper. We gave the model the extraction instructions shown below for each column.

- **Study Type**:

  Extract the type of study and research approach used, specifically noting:

- Study design (conceptual analysis, cross-sectional survey, longitudinal study, intervention trial, implementation study, etc.)
- Whether it's conceptual, empirical, or implementation research
- Research methodology (quantitative, qualitative, mixed methods)
- Data collection approach if empirical

- **Mental Health Context**:

  Extract details about the mental health context where the Levesque framework is being applied, including:

- Target population (age group, specific mental health conditions, demographics)
- Mental health service setting (primary care, specialty mental health, community services, schools, etc.)
- Geographic location and health system context
- Specific mental health issues addressed (ADHD, depression, anxiety, psychosis, etc.)

- **Framework Application**:

  Extract exactly how the Levesque et al. (2013) patient-centred access framework is being used in the study, including:

- Primary purpose (conceptual model, measurement tool, intervention design guide, evaluation framework, etc.)
- Whether it's the main theoretical framework or one of several cited
- Any modifications or adaptations made to the original framework
- How it's integrated with other models or frameworks
- Specific tools or instruments developed based on the framework

- **Access Dimensions**:

  Extract which specific access dimensions from the Levesque framework are operationalized or discussed, noting:

- Supply-side dimensions: Approachability, Acceptability, Availability/Accommodation, Affordability, Appropriateness
- Demand-side abilities: Ability to perceive, Ability to seek, Ability to reach, Ability to pay, Ability to engage
- How each dimension is defined or measured in the mental health context
- Any dimensions that are explicitly excluded or not addressed
- Novel operationalizations specific to mental health services

- **Access Findings**:

  Extract key findings related to mental health service access using the Levesque framework, including:

- Main barriers and facilitators identified for each access dimension
- Quantitative results (timing, rates, disparities) if available
- Qualitative insights about access experiences
- Differences across population subgroups
- Recommendations for improving access based on framework application
- How findings contribute to understanding mental health service access

- **Implementation Details**:

  If the study involves implementation or intervention based on the Levesque framework, extract:

- What specific interventions or changes were implemented to improve access
- Which access dimensions the implementation targeted
- Implementation setting and duration
- Implementation outcomes or process measures
- Barriers and facilitators to implementing framework-based changes
- Sustainability considerations
- If not an implementation study, note 'Not applicable'

Results

Characteristics of Included Studies

Study	Full Text Retrieved?	Study Type	Mental Health Context	Geographic Setting	Target Population
Ma Magdalena Martínez-Jaime et al., 2024	Yes	Retrospective cohort study; empirical; quantitative [1]	ADHD diagnosis at specialized mental health services in a tertiary hospital [1]	Mexico City, Mexico [1]	Children and adolescents with ADHD and their primary caregivers [1]
Christopher R. Harper et al., 2018	No	Conceptual analysis; no new empirical data collection [2]	Depression, suicide, and general preventive health services across primary care and community settings [2]	United States (implied) [2]	Adolescents and young adults [2]

The two included studies differ substantially in design, setting, and purpose. Martínez-Jaime et al. (2024) is an empirical investigation conducted at the only tertiary psychiatric hospital for children and adolescents in Mexico's public health sector [1], whereas Harper et al. (2018) is a conceptual commentary synthesizing existing evidence in the United States context [2]. Both focus on youth populations, though the former targets a specific clinical condition (ADHD) while the latter addresses a broader range of adolescent and young adult health concerns including depression and suicide [2].

Application of the Levesque Framework

Study	Role of Levesque Framework	Main or Supplementary Framework?	Modifications/Adaptations	Tools Developed	Integration with Other Frameworks
Ma Magdalena Martínez-Jaime et al., 2024	Conceptual model guiding the design of an ”Access Pathway” and a data collection instrument [1]	Main theoretical framework [1]	Adapted into the AAcceDa survey (semi-structured questionnaire), validated through face validity [1]	AAcceDa survey (48 dichotomous and polytomous questions) [1]	Integrated with the AAcceDa survey to evaluate access to mental health services [1]

Study	Role of Levesque Framework	Main or Supplementary Framework?	Modifications/Adaptations	Tools Developed	Integration with Other Frameworks
Christopher R. Harper et al., 2018	Referenced as one of several existing frameworks describing factors impacting healthcare utilization [2]	Not the main framework; the social-ecological model is foregrounded [2]	No modifications mentioned [2]	None mentioned [2]	Described as reflecting principles of the social-ecological model [2]

Martínez-Jaime et al. (2024) represents a direct and substantive application of the Levesque framework, using it as the sole theoretical basis for constructing both an analytic pathway and a novel survey instrument [1]. Harper et al. (2018) cites the Levesque framework in passing, positioning it as one of multiple frameworks that align with the social-ecological model's multilevel perspective on healthcare access [2]. This distinction is important: the former operationalizes the framework empirically, while the latter invokes it conceptually without developing specific measures from it.

Access Dimensions Operationalized

Study	Supply-Side Dimensions Addressed	Demand-Side Abilities Addressed	Dimensions Not Addressed	Novel Operationalizations
Ma Magdalena Martínez-Jaime et al., 2024	Not explicitly operationalized [1]	Ability to perceive (age of perception), Ability to seek (age of search), Ability to reach (age of first contact and age of arrival at hospital), Ability to engage (age of diagnosis) [1]	Supply-side dimensions (Approachability, Acceptability, Availability/Accommodation, Affordability, Appropriateness) not explicitly discussed [1]	Access Pathway mapping five temporal stages of the diagnostic journey for ADHD [1]

Study	Supply-Side Dimensions Addressed	Demand-Side Abilities Addressed	Dimensions Not Addressed	Novel Operationalizations
Christopher R. Harper et al., 2018	Acceptability (youth-friendly services, confidentiality), Availability/Accommodation (flexible hours, provider- and clinic-level factors) [2]	Ability to perceive (parental role in encouraging care-seeking), Ability to seek (social media interventions) [2]	Affordability, Appropriateness not explicitly addressed [2]	None specific to mental health [2]

The two studies are complementary in their dimensional coverage. Martínez-Jaime et al. (2024) focuses almost exclusively on demand-side abilities, translating each into a measurable time point along the diagnostic pathway [1]. Harper et al. (2018) attends more to supply-side dimensions—particularly Acceptability and Availability/Accommodation—through its discussion of youth-friendly service models and confidentiality protections [2]. Neither study addresses all ten dimensions of the Levesque framework, and notably, Affordability is absent from both [1, 2].

Key Findings on Mental Health Service Access

Study	Main Barriers	Main Facilitators	Quantitative Findings	Subgroup Disparities	Recommendations
Ma Magdalena Martínez-Jaime et al., 2024	Difficulty identifying core ADHD symptoms; inaccessible specialized care due to distance; long wait times [1]	Teachers detecting symptoms and urging caregivers to seek help [1]	71% of dyads experienced late or highly late diagnosis; girls diagnosed one year later than boys; both genders took one year to seek specialized care and an additional year to receive formal diagnosis [1]	Girls diagnosed later than boys; children with more siblings experienced longer delays; caregiver formal employment facilitated timelier diagnosis [1]	Reorganize diagnostic services starting from schools; improve collaboration between mental health and educational authorities [1]

Study	Main Barriers	Main Facilitators	Quantitative Findings	Subgroup Disparities	Recommendations
Christopher R. Harper et al., 2018	Confidentiality concerns, especially among those with private insurance [2]	Youth-friendly services; parental involvement; school-based health centers; social media interventions [2]	86% of children had a provider visit in the past year vs. 56% of young adults; 96% of children had a regular source of care vs. 75% of young adults [2]	Confidentiality concerns more pronounced among those covered by private insurance vs. Medicaid [2]	Increase youth-friendly providers and clinics; engage parents, schools, and social media [2]

Both studies document substantial access gaps for young people seeking mental health-related services. Martínez-Jaime et al. (2024) provides granular evidence that 71% of children with ADHD in Mexico City received a late diagnosis, with girls systematically disadvantaged relative to boys by approximately one year [1]. The study identifies teachers as the sole facilitator in the diagnostic pathway, given that caregivers often failed to recognize core symptoms as indicative of a mental health problem [1]. Harper et al. (2018) documents a steep decline in healthcare utilization between childhood and young adulthood in the United States, with provider visits dropping from 86% to 56% across this transition [2], and underscores that insurance coverage alone is insufficient to close this gap [2].

Implementation Considerations

Martínez-Jaime et al. (2024) proposes concrete implementation strategies derived from the framework application, including initiating the Access Pathway at schools where symptoms are first detected, utilizing existing infrastructure, and establishing referral systems across primary, secondary, and tertiary care levels [1]. Barriers to implementation include limited caregiver awareness and constrained availability of specialized mental health services, while facilitators include the active role of teachers and the potential for school-based care models [1]. Sustainability is envisioned through leveraging existing infrastructure rather than requiring new investment [1]. Harper et al. (2018) does not report on specific implementation activities, instead offering a conceptual discussion of potential multilevel interventions without empirical implementation data [2].

Synthesis

Despite their differences in design and setting, the two studies converge on several points relevant to applying the Levesque framework in mental health contexts. Both identify schools as a critical organizational setting for improving access—Martínez-Jaime et al. (2024) through evidence that teachers are the primary facilitators of ADHD detection [1], and Harper et al. (2018) through evidence that school-based health centers are a cost-effective means of increasing access [2]. Both also emphasize the importance of relationship-level factors: caregiver employment and family size shape diagnostic timeliness in the Mexico City context [1], while parental involvement in encouraging routine preventive care is highlighted in the U.S. context [2].

The studies differ in how deeply the Levesque framework is operationalized. Martínez-Jaime et al. (2024) demonstrates that the framework can be translated into a concrete measurement instrument (the AAcceDa survey) and a temporally structured Access Pathway with quantifiable endpoints [1, 1]. Harper et al. (2018) treats the framework

as one of several conceptual lenses, subordinating it to the social-ecological model without developing specific measures [2]. This difference reflects the distinction between empirical operationalization and conceptual citation of the framework.

A notable gap across both studies is the limited attention to supply-side dimensions. Martínez-Jaime et al. (2024) focuses almost entirely on demand-side abilities [1], while Harper et al. (2018) addresses Acceptability and Availability/Accommodation but does not operationalize them with specific metrics [2]. Affordability is unaddressed in both studies [1, 2], despite its likely relevance in both the Mexican public health system and the U.S. insurance landscape. The asymmetric coverage of the framework's dimensions suggests that future applications in mental health contexts would benefit from explicitly incorporating supply-side measurement alongside the demand-side temporal pathways that Martínez-Jaime et al. (2024) have effectively demonstrated.

References

1. Ma Magdalena Martínez-Jaime, H. Reyes-Morales, Ixchel Peyrot-Negrete, Mauricio Sebastián Barrientos-Álvarez (2024) Access to early diagnosis for attention-deficit/hyperactivity disorder among children and adolescents in Mexico City at specialized mental health services. BMC Health Services Research. https://doi.org/10.1186/s12913-024-11022-y

2. Christopher R. Harper, Riley J. Steiner, K. Brookmeyer (2018) Using the Social-Ecological Model to Improve Access to Care for Adolescents and Young Adults. Journal of Adolescent Health. https://doi.org/10.1016/j.jadohealth.2018.03.010
