# Supplementary material for: Patient-centered access to mental health care at selected life-course stages: a scoping review using the Levesque framework
Source: BMC Health Serv Res. 2026 Oct 22;26:1464. doi: 10.1186/s12913-026-15895-z (PMC13693167; doi:10.1186/s12913-026-15895-z)
Supplement: Supplementary file 5 [file 12913_2026_15895_MOESM10_ESM.docx]

Supplementary Material S7

# PRISMA ScR reporting checklist

Patient-centered access to mental health care at selected life-course stages: a scoping review using the Levesque framework

This checklist locates the reporting in manuscript version 2.14 and Supplementary Materials S1–S8. “Reported” describes the availability of an account, not certification of methodological quality or full adherence to JBI conduct recommendations. Items 10, 14 and 22 remain partially reported for the reasons stated below.

Locations use section and table names so that the checklist remains usable after final pagination. Items 12 and 16 are optional in PRISMA-ScR and are addressed because critical appraisal was undertaken.

| **Item** | **PRISMA ScR reporting requirement** | **Status** | **Location and implementation** |
| --- | --- | --- | --- |
| 1 | Identify the report as a scoping review. | Reported | Title; Methods — Design. The title identifies the report as a scoping review; descriptive evidence mapping and narrative synthesis describe the analytical approach. |
| 2 | Provide a structured summary including background, objectives, eligibility criteria, sources of evidence, charting methods, results, and conclusions. | Reported | Abstract. Structured summary of the rationale, objective, eligibility and source roles, identification routes, human verification, appraisal, findings and interpretation. |
| 3 | Describe the rationale for the review in the context of what is already known and explain why the questions/objectives lend themselves to a scoping-review approach. | Reported | Introduction. Rationale for the framework and selected life-course comparison, relevant existing syntheses, and the contribution of mapping framework use and access pathways. |
| 4 | Provide an explicit statement of the questions and objectives with reference to key elements. | Reported | Introduction — four review questions; Abstract — Objective. Questions address framework use, pathway stages, actors and service interfaces, and methodological/framework-use gaps. |
| 5 | Indicate whether a review protocol exists; state where it can be accessed and provide registration information. | Reported | Methods — Protocol and registration; S2. Registration dated 6 February 2026 at https://osf.io/8ntrb/; alignment with the registered plan and implemented procedures is described. |
| 6 | Specify eligibility characteristics, including years, language, publication status, and rationale. | Reported | Methods — Eligibility concepts; S2 and S6. Publication from 2013, English/German, target population, mental-health access, substantive framework use and completed findings; direct, adjacent and contextual roles are distinguished. |
| 7 | Describe all information sources and the date of the most recent search. | Reported | Methods — Source reports and searches; S4–S5. Elicit reports: 18 February and 31 July 2026; PubMed update and Cited by: 28 July 2026; Web of Science: 1 August 2026. |
| 8 | Present the full electronic search strategy for at least one database, including limits, so it can be repeated. | Reported | S5 — full PubMed update strategy and Web of Science citation route with all three population filters. S4 reproduces the four Elicit research questions and available screening/extraction instructions and reports. |
| 9 | State the process for selecting sources of evidence. | Reported | Methods — Source reports and searches, Eligibility concepts, and AI-assisted procedures and human oversight; Figure 1; S3–S5. Elicit automatic preselection and JCC’s human screening and final decisions are distinguished; selection was not independently duplicated. |
| 10 | Describe data-charting methods, including whether forms were calibrated and whether charting was independent or duplicated. | Partially reported | Methods — Data extraction and synthesis; S3 and S6. Standardized fields, preliminary Elicit extraction, JCC verification and BR’s independent full-text checking are described. Formal piloting or calibration of the charting form is not documented. |
| 11 | List and define all variables for which data were sought and assumptions/simplifications. | Reported | Methods — Data extraction and synthesis; S4 and S6. Bibliographic data, design, setting, population, pathway, framework use, dimensions, barriers/facilitators and key findings; separate source-type and evidence-role classification. |
| 12 | If done, provide a rationale for critical appraisal, methods used, and how appraisal informed synthesis. | Reported | Methods — Methodological quality appraisal and relevance classification; S1. MMAT/JBI rationale, design-specific criteria, independent ratings, correction of JBI NA to N, consensus procedure and interpretive use; appraisal did not determine inclusion. |
| 13 | Describe the methods of handling and summarizing charted data. | Reported | Methods — Data extraction and synthesis and Analytical Positioning. Descriptive mapping and narrative synthesis using Levesque pairs and four comparative questions; direct claims are distinguished from adjacent/contextual interpretation. |
| 14 | Give numbers screened, assessed, and included, with reasons for exclusions, ideally using a flow diagram. | Partially reported | Results — opening screening-flow paragraphs; Figure 1; S5. Reported totals: 372 entries, 5 duplicates, 367 screened, 297 title/abstract exclusions, 70 retrieved full texts, 33 full-text exclusions, 37 retained. The entry arithmetic is 50 + 20 + 300 + 2 = 372. No complete record-level log or exhaustive reason-specific exclusion list was maintained; the five reported duplicate removals and source-by-source flow cannot be independently audited. |
| 15 | For each source, present characteristics and citations. | Reported | Results — Overview of the evidence base; Table 1; S6. Characteristics and citations for all 37 sources are supplied in S6; Table 1 places the direct and selected adjacent/contextual studies before thematic synthesis. |
| 16 | If done, present critical-appraisal data. | Reported | Results — Methodological quality appraisal and evidence classification; Table 3; S1. Final item-level consensus judgments for 23 empirical studies and 11 reviews, including mixed-methods components; agreement is calculated separately from preconference ratings. |
| 17 | For each included source, present relevant charted data related to the questions/objectives. | Reported | Results — population-specific and thematic synthesis; Tables 1–2; S6. Source-level framework use, relevant access findings and evidence role are charted; direct evidence does not necessarily represent a first-person account. |
| 18 | Summarize and/or present charting results in relation to the review questions and objectives. | Reported | Results — Thematic synthesis across Levesque dimensions and Methodological gaps; Tables 2, 4 and 5; S6. Findings address pathway stages, gatekeepers, interfaces, acceptability and framework operationalization. |
| 19 | Summarize main results, link to review questions/objectives, and consider relevance to key groups. | Reported | Discussion. Interprets the comparative pathway patterns, population-specific mechanisms, respondent perspectives and implications for services and research while retaining evidence-role distinctions. |
| 20 | Discuss limitations of the review process. | Reported | Strengths and limitations. AI retrieval/preselection/extraction risks, single-reviewer selection, incomplete screening documentation, uneven evidence, mixed source types, framework measurement and respondent-perspective limitations. |
| 21 | Provide a general interpretation and potential implications/next steps. | Reported | Conclusion; Discussion; Table 4. Exploratory implications and next steps for paired measurement, pathway-level interventions, age-specific reporting and accessible direct participation. |
| 22 | Describe funding for included sources and the review and the role of funders. | Partially reported | Declarations — Funding. The review received no external funding. Funding and funder roles of individual included sources were not systematically charted and are not reported for each source. |

Checklist source: Tricco AC, Lillie E, Zarin W, et al. PRISMA Extension for Scoping Reviews (PRISMA-ScR): Checklist and Explanation. Annals of Internal Medicine. 2018;169(7):467–473. doi:10.7326/M18-0850.
