# Supplementary material for: Patient-centered access to mental health care at selected life-course stages: a scoping review using the Levesque framework
Source: BMC Health Serv Res. 2026 Oct 22;26:1464. doi: 10.1186/s12913-026-15895-z (PMC13693167; doi:10.1186/s12913-026-15895-z)
Supplement: Supplementary file 6 [file 12913_2026_15895_MOESM11_ESM.docx]

Supplementary Material S8

# **GAMER reporting checklist**

*Patient-centered access to mental health care at selected life-course stages: a scoping review using the Levesque framework*

This checklist locates the reporting of generative-AI use in manuscript V2.14 and its supplements. “Partially reported” identifies unavailable metadata or incomplete historical documentation; it does not assert full GAMER compliance. “Reported” indicates that the requested description is present, not that the AI-assisted workflow has been independently validated. Locations use section names so they remain usable after pagination changes.

| **No.** | **GAMER checklist item** | **Status** | **Reported location** | **Project-specific reporting** |
| --- | --- | --- | --- | --- |
| 1 | Did you use any GAI tools (such as large language models or large visual models) in any section or step of this manuscript or study? | Reported | Methods — AI-assisted procedures and human oversight; AI-use declaration; S3 sections 1–5 and 8 | Elicit supported retrieval, ranking, automatic preselection and draft extraction. ChatGPT supported documentation, manuscript revision, September appraisal-workbook calculations, and the 1 October 2026 editorial consistency revision. Human responsibility is explicitly declared. |
| 2 | Specify the GAI tool(s), versions and/or release dates, and dates/period of use. | Partially reported | Methods — Source reports and searches; AI-assisted procedures and human oversight; S3 sections 2–4 and 9 | Elicit reports: 18 February and 31 July 2026; internal model/version unavailable. ChatGPT: GPT-5.5 Pro on 6 May; GPT-5.6 Pro during July–August; GPT-6 Astra Ultra on 24 September 2026 (author-reported). Other September model versions are not documented. The 1 October 2026 session supported editorial preparation; no independent technical model-version record was retained in the project materials. |
| 3 | Describe prompting techniques used to generate manuscript content or perform analyses and provide unedited responses to prompts. | Partially reported | S3 sections 3–4 and 9; S4 — ELICIT-01 to ELICIT-04 | The four original reports, research questions, screening criteria and extraction instructions are documented. ChatGPT tasks are summarized. A complete set of unedited historical prompts and responses is not provided; private exchanges contain manuscript text and confidential editorial correspondence. |
| 4 | If a new GAI tool was developed or fine-tuned from an existing model, report the original model name and version. | Not applicable | S3 section 2 | No new generative-AI model was developed or fine-tuned for this review. |
| 5 | Describe the role of GAI tools in all phases of the study where used, including manuscript writing. | Reported | Methods — Data extraction and synthesis; AI-assisted procedures and human oversight; AI-use declaration; S3 sections 2–5 and 8 | The report differentiates Elicit automatic preselection from JCC’s final eligibility decisions and 33 human full-text exclusions. BR independently checked extracted data and completed appraisal, but did not independently duplicate screening. September AI support included statistics and revision; appraisal judgments remained human. |
| 6 | Report the specific sections or paragraphs of the manuscript to which GAI tools contributed. | Partially reported | Methods — AI-assisted procedures and human oversight; AI-use declaration; S3 sections 4–5, 8–9 | Task-level contributions to manuscript structure, wording, methods documentation, appraisal results and associated discussion are identified. A complete paragraph-by-paragraph record linking every historical AI output to the final text was not maintained. |
| 7 | Describe how GAI-generated content was verified and, where necessary, modified. | Reported with limits | Methods — Data extraction and synthesis; Methodological quality appraisal and relevance classification; S3 sections 5–6; S1 and S5–S6 | JCC checked retained extraction fields and made final eligibility, evidence-role and framework-coding decisions. BR independently checked extracted data against full texts. JCC/BR made the appraisal judgments and resolved discrepancies. No complete screening list or independent validation of the AI workflow is available; appraisal κ describes human agreement only. |
| 8 | Describe how data privacy and confidentiality were ensured during GAI use. | Partially reported | AI-use declaration; S3 section 7 | No participant-level, patient-level or identifiable clinical data were entered. Authors’ drafts and confidential editorial correspondence were used in a private revision context; private correspondence is excluded from the public log. Historical provider retention/training settings and a separate confidentiality assessment were not documented. |
| 9 | Describe whether and how GAI use may have influenced interpretation, overall accuracy, or conclusions. | Reported with limits | Strengths and limitations; S3 sections 5 and 9 | Potential omissions from retrieval/ranking and automatic preselection, extraction errors and model/platform variability are acknowledged. Human checks and complementary searches address these risks without establishing complete coverage. No comparative analysis quantified the effect of AI use on conclusions; incomplete screening documentation limits retrospective auditing. |

**Checklist source:** Luo X et al. Reporting guideline for the use of Generative Artificial intelligence tools in MEdical Research: the GAMER Statement. BMJ Evid Based Med. 2025;30(6):390–400. doi:10.1136/bmjebm-2025-113825.
