# Supplementary material for: Patient-centered access to mental health care at selected life-course stages: a scoping review using the Levesque framework
Source: BMC Health Serv Res. 2026 Oct 22;26:1464. doi: 10.1186/s12913-026-15895-z (PMC13693167; doi:10.1186/s12913-026-15895-z)
Supplement: Supplementary file 7 [file 12913_2026_15895_MOESM12_ESM.docx]

Supplementary Table S1

Item level methodological appraisal and evidence roles

Patient-centered access to mental health care at selected life-course stages: a scoping review using the Levesque framework

# Scope and appraisal procedure

The evidence map within this scoping review comprises 37 sources: 23 empirical primary-study entries, 11 reviews or evidence syntheses, one study protocol, and two conceptual or framework-level sources. The 34 appraised source entries contribute 312 item judgments: 161 MMAT screening and design-specific judgments, 30 additional mixed-methods component judgments, and 121 JBI judgments. The component rows belong to three of the 23 MMAT entries and are not additional sources.

Empirical entries were appraised with the Mixed Methods Appraisal Tool (MMAT) 2018; reviews were appraised with the JBI Critical Appraisal Checklist for Systematic Reviews and Research Syntheses.

JCC and BR independently appraised the sources and independently revised their assessments before the consensus conference. Agreement below describes those revised preconference judgments. Remaining differences were resolved to produce the final consensus ratings reported in this table. All 312 finalized rating pairs agree; this post-consensus concordance is not an independent reliability estimate.

Appraisal informed interpretation and did not determine inclusion or evidence role. No global MMAT score, combined quality score, or overall quality grade was calculated. Direct evidence comprises eight empirical sources and two review-level sources; directness concerns the eligibility and substantive framework-use criteria and does not necessarily denote first-person participant evidence.

# Preconference agreement

| **Item set** | **Agreement** | **Percent agreement** | **Cohen κ** |
| --- | --- | --- | --- |
| MMAT main items including screening | 152/161 | 94.4% | 0.853 |
| Additional mixed-methods component items | 28/30 | 93.3% | 0.870 |
| JBI review items after coding correction | 117/121 | 96.7% | 0.937 |
| All appraisal items pooled (descriptive) | 297/312 | 95.2% | 0.895 |
| Pooled sensitivity analysis excluding screening | 251/266 | 94.4% | 0.890 |

The sensitivity analysis excludes 46 MMAT screening pairs, all of which agreed. For pooled analyses only, MMAT Cannot tell and JBI Unclear were harmonized as one uncertainty category. Tool-specific results retain the original response categories.

# Rating and role conventions

Y = Yes; N = No; CT = Cannot tell (MMAT); U = Unclear (JBI). S1 and S2 are MMAT screening questions; Q1–Q5 are design-specific criteria; C1–C11 are JBI review criteria. Direct and adjacent empirical roles refer to primary-study entries. Direct, adjacent, and contextual review roles refer to evidence syntheses. Criterion descriptions below are concise labels for the corresponding checklist items.

# S1A MMAT qualitative studies

Final consensus ratings for 13 qualitative sources. S1 concerns the clarity of the research questions; S2 concerns whether the collected data address those questions.

| **ID** | **Source** | **Population** | **Evidence role** | **S1** | **S2** | **Q1** | **Q2** | **Q3** | **Q4** | **Q5** |
| --- | --- | --- | --- | --- | --- | --- | --- | --- | --- | --- |
| E02 | Ash et al. 2026 | Perinatal | Direct empirical | Y | Y | Y | Y | Y | Y | Y |
| E03 | Salameh et al. 2025 | Perinatal | Direct empirical | Y | Y | Y | Y | Y | Y | CT |
| E06 | Schwarz et al. 2022 | Older adults | Direct empirical | Y | Y | Y | Y | Y | Y | CT |
| E14 | Viveiros & Darling 2018 | Perinatal | Direct empirical | Y | Y | Y | Y | Y | Y | Y |
| E16 | DeRoche et al. 2023 | Perinatal | Adjacent empirical | Y | Y | Y | CT | Y | Y | CT |
| E19 | Fisher et al. 2024 | Perinatal | Adjacent empirical | Y | Y | Y | Y | Y | Y | Y |
| E23 | Dableh et al. 2025 (co-design) | Older adults | Adjacent empirical | Y | Y | Y | Y | Y | Y | Y |
| E24 | Dableh et al. 2025 (qualitative inquiry) | Older adults | Adjacent empirical | Y | Y | Y | Y | Y | Y | Y |
| E25 | Kurpas et al. 2018 | Older adults | Adjacent empirical | Y | Y | Y | CT | Y | Y | Y |
| E26 | Ramakrishnan & Malhotra 2025 | Dementia / older adults | Adjacent empirical | Y | Y | Y | Y | Y | Y | Y |
| E27 | Daskalska et al. 2025 | Youth | Direct empirical | Y | Y | Y | CT | Y | Y | Y |
| E34 | Fathi Afshar 2019 | Refugee minors | Adjacent empirical | Y | Y | Y | CT | Y | Y | Y |
| E35 | Williams-Ridgway et al. 2026 | Youth / young adults | Adjacent empirical | Y | Y | Y | Y | Y | Y | Y |

## Qualitative criteria

Q1: suitability of the qualitative approach. Q2: adequacy of data collection. Q3: derivation of findings from the data. Q4: substantiation of interpretations by the data. Q5: coherence of data sources, collection, analysis, and interpretation.

Fisher et al. (E19) is appraised as a qualitative article-level report. Dableh et al. (E23) is a qualitative co-design study; embedded prioritization and voting do not create a separate quantitative component.

# S1A MMAT quantitative studies

## Quantitative nonrandomized studies

Final consensus ratings for five sources. S1 and S2 use the screening definitions on the preceding page.

| **ID** | **Source** | **Population** | **Evidence role** | **S1** | **S2** | **Q1** | **Q2** | **Q3** | **Q4** | **Q5** |
| --- | --- | --- | --- | --- | --- | --- | --- | --- | --- | --- |
| E05 | Martínez-Jaime et al. 2024 | Youth | Direct empirical | Y | Y | N | N | N | N | CT |
| E13 | de Jong-Bardelmeijer et al. 2026 | Perinatal / postpartum | Adjacent empirical | Y | Y | N | Y | Y | N | CT |
| E17 | Koire et al. 2024 | Perinatal | Adjacent empirical | Y | Y | N | N | Y | N | CT |
| E32 | Kingston et al. 2014 | Perinatal | Adjacent empirical | Y | Y | N | N | Y | CT | Y |
| E33 | Zivin et al. 2024 | Perinatal | Adjacent empirical | Y | Y | CT | Y | Y | N | CT |

Nonrandomized criteria — Q1: representativeness of participants. Q2: appropriateness of outcome and exposure or intervention measurements. Q3: completeness of outcome data. Q4: handling of confounding in design and analysis. Q5: whether the intervention or exposure occurred as intended.

## Quantitative descriptive studies

| **ID** | **Source** | **Population** | **Evidence role** | **S1** | **S2** | **Q1** | **Q2** | **Q3** | **Q4** | **Q5** |
| --- | --- | --- | --- | --- | --- | --- | --- | --- | --- | --- |
| E29 | Adams 2024 | Youth | Direct empirical | Y | Y | Y | Y | Y | CT | CT |
| E37 | Mejía-Grueso et al. 2025 | Older adults | Adjacent empirical | Y | Y | Y | Y | N | CT | N |

Descriptive criteria — Q1: relevance of the sampling strategy. Q2: representativeness of the sample. Q3: appropriateness of measurements. Q4: low risk of nonresponse bias. Q5: appropriateness of statistical analysis.

# S1A MMAT mixed methods studies and components

Final consensus ratings for three mixed-methods sources. The main table contains screening and mixed-methods integration criteria; the two component tables add five qualitative and five quantitative judgments for each source.

| **ID** | **Source** | **Population** | **Evidence role** | **S1** | **S2** | **Q1** | **Q2** | **Q3** | **Q4** | **Q5** |
| --- | --- | --- | --- | --- | --- | --- | --- | --- | --- | --- |
| E18 | Blackmore et al. 2022 | Perinatal | Adjacent empirical | Y | Y | Y | CT | CT | CT | N |
| E22 | Bartlett et al. 2025 | Dementia / older adults | Adjacent empirical | Y | Y | Y | Y | Y | Y | N |
| E28 | Daskalska et al. 2024 | Youth | Direct empirical | Y | Y | N | N | N | N | N |

Mixed-methods criteria — Q1: rationale for combining methods. Q2: integration of components. Q3: interpretation of integrated results. Q4: handling of divergent or inconsistent results. Q5: quality of the individual components.

## Qualitative components

| **ID** | **Source** | **Qual Q1** | **Qual Q2** | **Qual Q3** | **Qual Q4** | **Qual Q5** |
| --- | --- | --- | --- | --- | --- | --- |
| E18 | Blackmore et al. 2022 | Y | Y | Y | Y | Y |
| E22 | Bartlett et al. 2025 | Y | Y | CT | Y | Y |
| E28 | Daskalska et al. 2024 | Y | Y | Y | Y | Y |

## Quantitative components

| **ID** | **Source** | **Quantitative category** | **Quant Q1** | **Quant Q2** | **Quant Q3** | **Quant Q4** | **Quant Q5** |
| --- | --- | --- | --- | --- | --- | --- | --- |
| E18 | Blackmore et al. 2022 | Quantitative descriptive | Y | CT | Y | CT | CT |
| E22 | Bartlett et al. 2025 | Quantitative descriptive | Y | N | Y | N | Y |
| E28 | Daskalska et al. 2024 | Quantitative non-randomized | CT | CT | CT | N | CT |

Component items use the qualitative and quantitative definitions on the preceding pages. E18 and E22 use the quantitative descriptive set; E28 uses the quantitative nonrandomized set.

E18 is a linked project-level mixed-methods appraisal using companion empirical reports. Blackmore et al. (2022) is the integration report and is not counted as a new independent sample. These component ratings do not create additional entries in the 37-source evidence map.

# S1B JBI reviews and evidence syntheses

Final consensus ratings for 11 reviews or evidence syntheses. U denotes Unclear. Criterion labels appear on the following page.

| **ID** | **Source** | **Population or domain** | **Evidence role** | **C1** | **C2** | **C3** | **C4** | **C5** | **C6** | **C7** | **C8** | **C9** | **C10** | **C11** |
| --- | --- | --- | --- | --- | --- | --- | --- | --- | --- | --- | --- | --- | --- | --- |
| E07 | Sambrook Smith et al. 2019 | Perinatal | Adjacent review | Y | Y | Y | N | Y | N | N | Y | N | Y | Y |
| E08 | Webb et al. 2023 | Perinatal | Adjacent review | Y | Y | Y | N | U | Y | Y | U | N | Y | Y |
| E09 | Cu et al. 2021 | Framework / general health care | Contextual review | Y | Y | N | Y | N | N | Y | Y | N | N | Y |
| E10 | Lowther-Payne et al. 2023 | Adult mental health | Contextual review | Y | Y | Y | N | N | Y | Y | Y | Y | Y | Y |
| E11 | Werlen et al. 2020 | Youth | Adjacent review | Y | Y | Y | N | Y | Y | Y | Y | N | Y | Y |
| E12 | Barbo & Petrucka 2024 | Youth refugees | Adjacent review | Y | N | Y | Y | N | U | N | U | U | N | Y |
| E15 | Marshman et al. 2023 | Perinatal | Direct review | Y | Y | Y | N | N | Y | N | Y | N | N | Y |
| E20 | Lewkowitz et al. 2024 | Perinatal | Adjacent review | Y | Y | Y | Y | Y | Y | Y | Y | Y | Y | Y |
| E21 | Bali et al. 2024 | Perinatal / migrant maternity care | Adjacent review | Y | Y | Y | N | N | Y | U | Y | N | Y | Y |
| E31 | Viveiros & Darling 2019 | Perinatal | Direct review | Y | Y | Y | Y | N | N | U | Y | N | Y | U |
| E36 | Yılmaz et al. 2025 | Forcibly displaced youth | Adjacent review | Y | Y | Y | N | Y | U | U | U | N | U | Y |

All incorrectly assigned JBI NA ratings were recoded uniformly to N. In the paired preconference data, these were four already concordant NA/NA pairs: E07 C9, E08 C9, E09 C6, and E21 C9. The correction was applied consistently to the preconference and final sets and did not change the number of agreeing pairs. This study-specific correction does not imply that Not applicable is universally prohibited by the JBI checklist.

# S1B JBI review criterion labels

C1 Clarity of the review question.

C2 Suitability of inclusion criteria for the review question.

C3 Appropriateness of the search strategy.

C4 Adequacy of the sources and resources searched.

C5 Appropriateness of the critical appraisal criteria.

C6 Independent critical appraisal by at least two reviewers.

C7 Methods to minimize data extraction errors.

C8 Appropriateness of methods used to combine studies.

C9 Assessment of the likelihood of publication bias.

C10 Support of policy or practice recommendations by the reported data.

C11 Appropriateness of directions for future research.

# S1C Sources not formally appraised

| **ID** | **Source** | **Population or domain** | **Source type** | **Evidence role** | **Reason not appraised** |
| --- | --- | --- | --- | --- | --- |
| E01 | Levesque et al. 2013 | Framework | Conceptual framework paper | Framework-level / conceptual | Originating conceptual framework; no empirical findings to appraise. |
| E04 | Harper et al. 2018 | Youth / young adults | Conceptual commentary | Contextual conceptual | Conceptual commentary; no completed empirical findings. |
| E30 | Berger et al. 2020 | Perinatal | Qualitative study protocol | Protocol evidence | Study protocol; no completed empirical findings. |

These sources support conceptual, contextual, or protocol interpretation and do not contribute to the direct empirical or direct review-level evidence counts.

# S1D Resolution of preconference disagreements

Fifteen item pairs differed after the independent preconference revisions. The table retains each reviewer’s preconference judgment alongside the final consensus judgment. CT and U retain their tool-specific meanings. All other preconference item pairs agreed.

| **ID** | **Source** | **Item set** | **Item** | **JCC before consensus** | **BR before consensus** | **Final consensus** |
| --- | --- | --- | --- | --- | --- | --- |
| E03 | Salameh et al. 2025 | MMAT main | Q5 | Y | CT | CT |
| E06 | Schwarz et al. 2022 | MMAT main | Q5 | Y | CT | CT |
| E16 | DeRoche et al. 2023 | MMAT main | Q5 | Y | CT | CT |
| E25 | Kurpas et al. 2018 | MMAT main | Q2 | Y | CT | CT |
| E17 | Koire et al. 2024 | MMAT main | Q1 | Y | N | N |
| E37 | Mejía-Grueso et al. 2025 | MMAT main | Q2 | CT | Y | Y |
| E13 | de Jong-Bardelmeijer et al. 2026 | MMAT main | Q4 | CT | N | N |
| E18 | Blackmore et al. 2022 | MMAT main | Q3 | Y | CT | CT |
| E28 | Daskalska et al. 2024 | MMAT main | Q1 | Y | N | N |
| E09 | Cu et al. 2021 | JBI | C10 | U | N | N |
| E12 | Barbo & Petrucka 2024 | JBI | C9 | N | U | U |
| E12 | Barbo & Petrucka 2024 | JBI | C10 | U | N | N |
| E21 | Bali et al. 2024 | JBI | C10 | U | Y | Y |
| E22 | Bartlett et al. 2025 | MMAT component | Qual Q3 | CT | Y | CT |
| E28 | Daskalska et al. 2024 | MMAT component | Quant Q5 | CT | N | CT |

The final consensus column is reproduced from the corresponding item-level appraisal tables. The corrected JBI NA/NA pairs were already concordant and are therefore not listed as disagreements.
