# Supplementary material for: Patient-centered access to mental health care at selected life-course stages: a scoping review using the Levesque framework
Source: BMC Health Serv Res. 2026 Oct 22;26:1464. doi: 10.1186/s12913-026-15895-z (PMC13693167; doi:10.1186/s12913-026-15895-z)
Supplement: Supplementary file 8 [file 12913_2026_15895_MOESM5_ESM.docx]

Supplementary Material S2 Protocol alignment and implementation

**Patient-centered access to mental health care at selected life-course stages: a scoping review using the Levesque framework**

This supplement compares the scoping review and its framework-guided evidence mapping with the preserved protocol summary. The Levesque model was planned from the start. The comparison distinguishes that initial choice from the exact registered eligibility wording and documents the implemented search, selection, source classification, and appraisal procedures. It reflects manuscript V2.14, with editorial consistency corrections on 1 October 2026 to the documentation available through 24 September 2026.

# Protocol source and scope

The author identifies the preregistration as 6 February 2026 at https://osf.io/8ntrb. That registration could not be accessed for this revision, so its date and contents have not been independently verified. The broader project at https://osf.io/fv2ap is a separate reference and is not substituted for the identified registration.

The preserved S2 V2.0 summary names the protocol “Access To And Utilisation Of Mental Health Services Among Children And Adolescents, Pregnant And Postpartum People, And Older Adults”. It describes a PRISMA-ScR-oriented scoping review of empirical evidence for children and adolescents aged 0–17 years, pregnant or postpartum people, and adults aged 65 years and older. It describes descriptive synthesis and mapping to Levesque et al. (2013) “where applicable”. These are statements preserved in the summary, not a new verification of the registered text.

# Summary assessment

The life-course populations, descriptive aim, and initial choice of the Levesque model are consistent with the preserved summary and the author’s clarification. The exact relationship between its “where applicable” wording and the manuscript’s substantive-framework-use eligibility requires comparison with the original registered wording. The available account does not establish a later thematic narrowing, a change of model, or when any eligibility decision was made relative to final source selection.

The implemented identification routes, inclusion of separately labelled review and contextual sources, and limits of the selection record differ from or add detail to the preserved summary. The table below states the basis for each comparison. Its status labels do not establish deviations from wording that has not been checked in the registration.

# Detailed comparison

| **Protocol domain** | **Preserved protocol summary** | **Current implementation** | **Comparison and reporting implication** |
| --- | --- | --- | --- |
| **Review aim and hypotheses** | Descriptive mapping of access to and utilization of mental health services across the three populations; no hypothesis testing. | The manuscript maps barriers, facilitators, and framework applications. No inferential hypothesis testing is conducted. | Consistent with the preserved summary. Retain the descriptive purpose. |
| **Population scope** | Children and adolescents aged 0–17 years; pregnant or postpartum people; adults aged 65 years and older. | These remain the target populations. Sources with mixed or adjacent populations are distinguished from direct population-specific evidence. | Target populations are consistent. Adjacent sources require explicit evidence-role labels; their use does not establish a later change in the target populations. |
| **Review type and reporting** | A PRISMA-ScR-oriented scoping review using transparent, reproducible methods. | The manuscript is a scoping review using the Levesque framework, with descriptive evidence mapping and narrative synthesis. Search routes and aggregate selection flow are reported; a complete record-level screening audit is unavailable. | The review designation is consistent with the scoping approach described in the preserved summary. It is based on the questions, extraction, and analysis, not on the number of searches. Incomplete screening documentation and single-reviewer selection remain limitations; full conformity with every JBI recommendation is not claimed. |
| **Search and identification** | Multiple bibliographic databases with predefined terms for mental health services, target populations, and related outcomes. | Three Elicit reports were dated 18 February 2026 and a youth-focused report 31 July 2026. The thematic PubMed search on 28 July retrieved 2 records: 0 direct sources, 1 source retained as adjacent in the final map (also found via ELICIT-04/citation), and 1 excluded protocol. PubMed Cited by, used on 28 July, contributed no additional candidates; this does not mean its displayed list was empty. Web of Science on 1 August returned 2,423 citing records and 185 youth, 48 perinatal, and 67 older-adult hits. S5 documents all three filters. | The implemented sequence differs from the database-led approach described in the preserved summary. Report its actual routes and counts without treating them as proof of comprehensive coverage. The preliminary 31 July Web of Science count of 2,419 is not added to the final counts. |
| **Date and language limits** | Publications from 2013 onwards in English or German. | Year, language, and source type must be distinguished from framework relevance. ELICIT-03 contains both “published after 2013” and “2013 or later”; this wording alone does not establish its precise technical date boundary. | No departure is established by that wording alone. This comparison does not independently verify every source’s compliance or the exact registered boundary. |
| **Empirical eligibility** | Empirical quantitative, qualitative, and mixed-methods evidence on service access or utilization in a target population. | Empirical studies are retained alongside separately classified reviews and evidence syntheses, a study protocol, and conceptual or contextual papers. | The source set extends beyond the empirical-study criterion in the preserved summary. Evidence roles distinguish direct empirical findings from adjacent, review, protocol, and conceptual contributions. |
| **Levesque framework** | Findings mapped to Levesque “where applicable”. | The author confirms that the model was planned from the start. The manuscript applies substantive framework-use eligibility and distinguishes direct and adjacent evidence. | Initial model choice is confirmed. Whether substantive framework use was an explicit registered eligibility condition remains unresolved without the original wording. The available documentation does not establish a model shift, later narrowing, or the timing of eligibility decisions. |
| **Study selection** | Title/abstract and full-text stages; independent screening by two reviewers where feasible, with discrepancy resolution. | Elicit performed automatic preselection. JCC reviewed the articles and excluded those not meeting the criteria; he personally made all 33 final exclusions after human full-text review. The reported human flow comprises 367 screened records and 70 assessed full texts. No screening list was kept. | Human final decisions are confirmed. Independent dual screening is not established. Because the preserved summary makes dual screening conditional, its absence cannot alone establish a protocol departure. Record-level auditability remains incomplete. |
| **Deduplication** | Import records into reference management software and remove duplicates. | The reported flow has 372 candidate-record entries and 367 records after deduplication, corresponding to 5 duplicates. The exact software workflow is not established here. | Deduplication counts are reported; a workflow departure is not established without further procedural evidence. Counts alone do not provide a complete record-level audit. |
| **Data charting** | A standardized charting form covering study characteristics, access/utilization, barriers and facilitators, health literacy, socioeconomic disadvantage, and critical life events. | Study information and Levesque-related findings were extracted and organized. BR independently checked extracted study data against the full texts. | Independent extraction verification is confirmed. Completeness against every field in the preserved summary has not been established; no blanket charting deviation is inferred. |
| **Quality appraisal** | Formal appraisal is not required as an inclusion criterion. | JCC and BR independently completed and revised MMAT ratings for 23 empirical studies and JBI ratings for 11 reviews before consensus. Preconference agreement was 297/312 (95.2%; descriptive pooled Cohen κ = 0.895). S1 reports final item-level ratings; one protocol and two conceptual sources were not appraised. | Appraisal is a methodological addition relative to the preserved summary. It informs interpretation and is not an inclusion rule. Tool-specific agreement estimates accompany the descriptive pooled estimate. |
| **Analysis and synthesis** | Descriptive synthesis and mapping of barriers and facilitators to Levesque dimensions across life-course groups; no effect-size meta-analysis. | The manuscript uses narrative synthesis and Levesque-based mapping across youth, perinatal, and older-adult evidence. No effect-size meta-analysis is conducted. | Consistent with the preserved summary. Keep descriptive patterns separate from causal or pooled-effect claims. |
| **Contextual sources** | Reference lists and relevant reviews may help identify eligible empirical studies. | Selected reviews, protocols, and conceptual or contextual papers also support framework rationale and interpretation. They are labelled separately in the source matrix. | Using these sources as interpretive evidence goes beyond the retrieval role described in the preserved summary. They do not replace direct empirical evidence for a target population. |
| **Interpretation** | Map the extent and characteristics of evidence without hypothesis tests or effect estimation. | Conclusions draw on the included evidence and acknowledge incomplete coverage and record-level auditability. | Consistent with the descriptive purpose, with limits made explicit. The partial retrospective account of selected decisions does not reconstruct an unrecorded screening list. |

# Reporting implications

The scoping review designation reflects the questions and detailed examination of framework applications and access barriers. Additional searches strengthened identification but did not determine the review type. The planned populations and initial use of Levesque are retained. Empirical, adjacent, review, protocol, and conceptual sources remain separately classified; appraisal informs interpretation.

S5 provides selected retrospective decisions, not a complete historical screening log. This limits auditability. S4 presents the four Elicit reports, search questions, screening criteria, and extraction instructions.

# Implemented methods

Evidence identification combined three Elicit reports dated 18 February 2026, a youth-focused report dated 31 July 2026, and non-AI database and citation searches. JCC reviewed the articles and made final eligibility decisions, including all 33 full-text exclusions. BR independently checked extracted study data against full texts. JCC and BR independently completed and revised methodological appraisals before consensus; S1 reports the final ratings and preconference agreement.

# Limitations of protocol comparison and implementation

The implemented searches and inclusion of separately labelled review and contextual sources differ from the preserved protocol summary. No complete screening log was kept, limiting reconstruction of exclusions. The exact registered relationship between mapping findings to Levesque “where applicable” and substantive-framework-use eligibility remains unresolved. These limits do not establish a later change in model or thematic focus.

# Source note

Comparison basis: preserved S2 V2.0 protocol summary, project documentation, and author clarifications through 24 September 2026. The registration at https://osf.io/8ntrb was inaccessible for independent checking; https://osf.io/fv2ap is the separate broader project. S1 documents appraisal, S3 human and AI roles, S5 searches and available decisions, and S6 source classification.
