# Supplementary material for: Patient-centered access to mental health care at selected life-course stages: a scoping review using the Levesque framework
Source: BMC Health Serv Res. 2026 Oct 22;26:1464. doi: 10.1186/s12913-026-15895-z (PMC13693167; doi:10.1186/s12913-026-15895-z)
Supplement: Supplementary file 9 [file 12913_2026_15895_MOESM6_ESM.docx]

Supplementary Material S3

AI assisted procedures and human oversight

Patient-centered access to mental health care at selected life-course stages: a scoping review using the Levesque framework

Manuscript V2.14 · Editorial documentation updated 1 October 2026; historical search and appraisal records through 24 September 2026

# 1 Documentation scope and provenance

This log records the AI-assisted activities, use of outputs and human oversight in the scoping review. It distinguishes documented procedures from unavailable historical details and does not reconstruct missing prompts or model versions. Jan Christopher Cwik (JCC) is the responsible author. The Levesque framework was selected during initial study planning, and the review was preregistered on 6 February 2026 (https://osf.io/8ntrb/). Supplementary Material S2 describes the relationship between the registered plan and the implemented methods. The broader project is identified separately at https://osf.io/fv2ap/.

Elicit was initially selected to obtain a broad literature overview quickly and efficiently. This was the authors’ purpose and practical rationale, not a finding of superior reliability or completeness. Non-AI database and citation searches were subsequently added to extend and check evidence identification.

The documentation covers the February 2026 Elicit reports, the 6 May 2026 documentation session, the July–August searches and revision, the September 2026 appraisal and manuscript updates, and the editorial consistency revision of 1 October 2026. Inputs comprised literature reports, published studies, search exports, appraisal workbooks, manuscript drafts, supplements and private editorial correspondence. No participant-level or patient-level data were processed.

# 2 Tools models and purposes

| **Tool and period** | **Model or version** | **Purpose and control** | **Documentation status** |
| --- | --- | --- | --- |
| Elicit 18 Feb and 31 Jul 2026 | Not displayed or retained | Semantic retrieval, relevance ranking, screening recommendations, and draft extraction. Authors defined the questions and criteria. | All four original report exports, research questions, screening criteria and extraction instructions are documented in S4; underlying model/version is unavailable. |
| ChatGPT 6 May 2026 | GPT-5.5 Pro | AI-documentation preparation and consistency checks; JCC reviewed and revised the text. | Principal request described in the historical documentation; intermediate prompts not separately logged. |
| ChatGPT July–August 2026 | GPT-5.6 Pro | Structure, translation, language, revision planning, tables, figures, and documentation. Authors reviewed suggestions. | Public log summarizes tasks; private editorial correspondence is not reproduced. |
| ChatGPT September 2026 | GPT-6 Astra Ultra 24 September 2026 | Appraisal-workbook analysis and agreement calculations, source comparison, manuscript and supplement revision. Human appraisal judgments remained authoritative. | Model name reported by the author for 24 September; other September model versions are not documented. |

# 3 Elicit activity log

All four original Elicit PDF reports are available. Supplementary Material S4 records their research questions, screening criteria, extraction instructions and report provenance under the identifiers ELICIT-01 to ELICIT-04. Platform outputs below are not counts of unique records independently screened by a human reviewer.

| **ID and date** | **Population and query scope** | **Platform output** | **Human use and adjudication** |
| --- | --- | --- | --- |
| ELICIT-01 18 Feb 2026 | Perinatal mental-health access; complete question available. | Up to 1,000 ranked records; 31 structured extraction outputs. | Contributed to the original evidence set. Retained source information was checked against the original publications; roles are recorded in S6. |
| ELICIT-02 18 Feb 2026 | Older-adult mental-health access; complete question available. | Up to 1,000 ranked records; 17 structured extraction outputs. | Contributed to the original evidence set. Mejía-Grueso et al. (2025) is identifiable in the original report; this establishes an ELICIT-02 route, not necessarily its first or only route. |
| ELICIT-03 18 Feb 2026 | Broader mental-health/Levesque question with documented “children, adolescents” keyword filter. | Up to 1,000 ranked records; two structured extraction outputs. | Identified Martínez-Jaime et al. and Harper et al. The original question was broader than ELICIT-04 but already included a youth-related keyword filter. |
| ELICIT-04 31 Jul 2026 | Dedicated question on substantive framework use in mental-health access for ages 0–17; complete question available. | 5,000 ranked records; 20 full-text candidates; three extraction proposals. | Martínez-Jaime was re-identified. Five adjacent sources were retained across the supplementary PubMed and youth Elicit routes: Werlen, Barbo & Petrucka, Fathi Afshar, Williams-Ridgway, and Yılmaz. Williams-Ridgway had also been identified by PubMed on 28 July and is counted only once. |

Dates above are the recorded project dates. PDF CreationDate metadata is consistent with them but, by itself, establishes export creation rather than independent proof of search execution.

Historical Elicit instructions and final eligibility criteria are distinguishable. ELICIT-01 allowed framework reference; ELICIT-02 allowed a majority aged 65 or older; final direct-evidence criteria required substantive framework use and findings attributable to the target population. ELICIT-03 records both “published after 2013” and “2013 or later”; its precise technical boundary cannot be established from the report alone.

The author confirmed that exclusions “via Elicit” referred to automatic preselection using framework use, data completeness, mental-health focus, and age/target population. JCC personally assessed articles and excluded them when the review criteria were unmet, making all 33 final full-text exclusion decisions. The four stated Elicit criteria do not establish record-level reasons for these final human exclusions. No screening list was kept. The available selected decisions provide a partial retrospective account based on sources, rather than a complete contemporaneous screening record.

The thematic PubMed update on 28 July 2026 retrieved two records. Neither met all direct-evidence criteria. Williams-Ridgway (PMID 41709297) was retained as adjacent evidence in the final map and also identified via ELICIT-04/citation; PMID 41878391 was an ineligible physical-activity intervention protocol. These are final evidence roles, not evidence of exclusive route provenance. PubMed’s “Cited by” function was also used on 28 July and contributed no additional candidates; its displayed total, filters and record-level overlap were not documented. The final Web of Science Core Collection citation search on 1 August returned 2,423 citing records. Population filters yielded 185 youth, 48 perinatal and 67 older-adult hits (300 before deduplication). The exported history and author-supplied syntax document all three filters. S5 records the editions, DOI-to-seed-to-Citations route, preliminary searches and separate zero-result Smart Search; these preliminary attempts are not added to the final counts.

# 4 Generative AI activity and prompt log

These are task summaries, not reconstructed verbatim prompts. Private conversations containing extensive manuscript text and confidential editorial material are not reproduced.

| **ID and period** | **Inputs and task** | **Output use and human control** |
| --- | --- | --- |
| GAI-01 6 May 2026 | Manuscript and documentation templates; prepare project-specific AI documentation without inventing missing details. | Draft documentation reviewed and revised by JCC. Intermediate prompts were not separately logged. |
| GAI-02 July–August 2026 | Manuscript versions, supplements, Elicit report, full texts, editorial comments, and author-provided counts; support revision and documentation. | Suggestions on text, tables, supplements, and Figure 1 were accepted, revised, or rejected by the authors. Scientific decisions remained human. |
| GAI-03 September 2026 | Final appraisal workbooks, full texts, source records, recovered Elicit reports, and author clarifications; calculate/check agreement, compare claims with sources, and revise manuscript and supplements. | JCC and BR supplied the appraisal judgments. Computational and drafting support used those judgments; source comparisons and edits were reviewed by the authors. No independent AI quality rating replaced their assessments. |
| GAI-04 1 October 2026 | Coauthor comments, manuscript V2.13, supplements and supplied figure; editorial consistency checks, clarification of route arithmetic, correction of a duplicated source identity, figure labels and document preparation. | Draft V2.14 prepared for author review. No new search, study inclusion or human appraisal was performed. Unrecoverable historical screening decisions were not reconstructed. Final author approval is required before resubmission. |

# 5 Output use and human verification

| **Process** | **AI contribution** | **Human control and current status** |
| --- | --- | --- |
| Retrieval and ranking | Elicit semantic retrieval and ranking. | Supplemented by database and citation searches. Residual coverage limitations are acknowledged. |
| Screening | Preliminary Elicit recommendations. | Automatic Elicit preselection preceded human screening. The human flow reports 367 records screened and 70 full texts assessed, with no report sought unavailable. JCC personally assessed articles and made all 33 final full-text exclusion decisions when criteria were unmet. No screening list was kept; record-level auditability is incomplete. |
| Extraction | Draft structured study fields. | JCC verified retained extraction fields. BR independently checked extracted study data against the corresponding full texts. JCC led final eligibility and evidence classification; BR’s check was not an independent screening round. |
| Appraisal | September computation and analysis of human ratings. | JCC and BR independently completed and revised MMAT/JBI judgments before consensus; JBI NA was uniformly recoded to N. Final ratings and instrument-specific and pooled descriptive agreement results are reported in S1. |
| Coding and synthesis | Preliminary organization, structure, and language support. | JCC led Levesque coding and narrative synthesis. BR contributed to the overall manuscript and final revision; the authors retained responsibility for interpretation. |

Preconference agreement was 297/312 item pairs (95.2%; descriptive pooled Cohen κ = 0.895). Incorrectly assigned JBI “not applicable” judgments were uniformly recoded to “no”. The analysis uses the independently revised ratings saved before the consensus conference. Final consensus ratings cover all 312 items. Agreement after consensus is not a separate reliability estimate; S1 documents the instrument-specific results, item ratings and preconference discrepancies.

These agreement statistics concern the two human appraisers.

# 6 Literature source and claim verification log

| **ID and category** | **Verification basis** | **Status and use** |
| --- | --- | --- |
| LIT-001 Retained sources and extraction | Original full texts, bibliographic records, and evidence-role criteria. | Retained sources and extracted study data verified against original publications; BR’s independent full-text check is confirmed. Retention follows the final evidence role, not an Elicit recommendation alone. |
| LIT-002 Citation-search candidates | Original publications and common eligibility criteria; S5 route and decision documentation. | Five incremental direct sources were retained across the supplementary citation-search route. The Web of Science history and author-supplied syntax document the database, editions, DOI-to-seed-to-Citations route, final 1 August counts and all three filters. PubMed “Cited by” on 28 July contributed no additional candidates; its total displayed count, any filters and record-level overlap were not documented (S5). |
| LIT-003 Dedicated youth follow-up | Original publications, recovered ELICIT-04 report, and author-provided human flow. | Of ten records without Elicit full-text access, six were retrieved and assessed: three retained as adjacent and three excluded. Four were excluded at human title/abstract screening and not sought. No screening list was kept; their titles/identifiers are not traceable in the available documentation. |
| LIT-004 Contextual and framework sources | Original publications and evidence-role criteria. | Verified and separately labelled; used for conceptual or contextual interpretation. |
| LIT-005 Appraisal and manuscript claims | Final human appraisal cells, agreement calculations, and full-text/source comparisons. | Completed appraisal integrated into the manuscript and S1. Authors reviewed source-to-claim alignment and revised interpretation; BR contributed to the final manuscript review. |

# 7 Data protection and confidentiality

No patient-level, participant-level, or identifiable clinical data were entered. Source material comprised manuscript drafts, supplements, published articles, Elicit reports, search exports, appraisal workbooks, and reviewer/editor correspondence.

Editor and reviewer comments were used privately for revision and are not reproduced verbatim in the public prompt log. No unrelated unreleased third-party manuscript was processed; the authors’ own unreleased drafts and supplements were used.

The public record uses summarized task-level documentation. Private conversation transcripts containing full manuscript passages or confidential editorial correspondence are not included.

Historical provider retention/training settings and a separate confidentiality assessment were not documented.

# 8 AI use declaration

AI-assisted tools supported evidence identification, preliminary screening and extraction, transparency documentation, and manuscript drafting and revision. Elicit was chosen to obtain a broad literature overview quickly and efficiently; subsequent non-AI database and citation searches supplemented this route. This rationale does not establish superior reliability or completeness. Elicit provided semantic retrieval, relevance ranking, screening recommendations, and draft structured extraction. Retained sources and extracted study data were verified against original publications, including an independent full-text check by BR. JCC led final eligibility and evidence classification, Levesque coding, and synthesis. Elicit exclusions referred to automatic preselection. JCC personally assessed the full texts and made all 33 final full-text exclusion decisions. No screening list was kept; available selected decisions are a partial retrospective account based on sources.

ChatGPT (OpenAI; GPT-5.5 Pro on 6 May 2026 and GPT-5.6 Pro during the July–August 2026 revision) supported structure, translation, language, revision planning, and documentation. During September 2026, including the revision on 24 September using GPT-6 Astra Ultra (author-reported), ChatGPT also supported appraisal-workbook analysis, calculation and checking of agreement statistics, full-text/source comparisons, and revision of the manuscript and supplements. JCC and BR made the appraisal judgments independently, revised them before consensus, and resolved remaining differences. BR also contributed to the overall manuscript and final revision. The authors reviewed AI-assisted outputs and retained responsibility for the scientific judgments, interpretation, accuracy, originality, and integrity of the work. No patient-level or identifiable clinical data were entered. Confidential editorial correspondence is excluded from the public prompt log.

# 9 Residual documentation limitations

• Elicit’s internal model/version and other September 2026 ChatGPT model versions were not documented. The author reports GPT-6 Astra Ultra for 24 September 2026. The full private prompt history is not publicly reproduced.

• All four original Elicit reports, their research questions, screening criteria and extraction instructions are documented in S4. Complete historical platform/model metadata and private generative-AI exchanges are not available as a public reproducibility record.

• Author verification of retained sources is documented, but no independent validation of the complete AI-assisted workflow was performed. The human appraisal-agreement statistics do not estimate Elicit retrieval sensitivity, screening accuracy or extraction accuracy. S8 records partial reporting where GAMER items cannot be fully documented.

• No screening list was kept. The 33 final full-text exclusions were human decisions by JCC, but a complete contemporaneous record linking each excluded article to its reason is unavailable. The available selected decisions provide a partial retrospective account based on sources. PubMed’s “Cited by” function was used on 28 July 2026 and contributed no additional candidates; its total displayed citing-record count, any filters, and record-level overlap are not documented. All three 1 August Web of Science population filters are fully documented. No missing records or exclusion frequencies have been inferred.

• Available task-level records describe AI contributions to manuscript sections and study procedures; they do not establish a complete paragraph-by-paragraph provenance history for all versions.
