# Supplementary material for: Patient-centered access to mental health care at selected life-course stages: a scoping review using the Levesque framework
Source: BMC Health Serv Res. 2026 Oct 22;26:1464. doi: 10.1186/s12913-026-15895-z (PMC13693167; doi:10.1186/s12913-026-15895-z)
Supplement: Supplementary file 10 [file 12913_2026_15895_MOESM7_ESM.docx]

Supplementary Material S4

# Elicit search instructions and human adjudication

*Patient-centered access to mental health care at selected life-course stages: a scoping review using the Levesque framework*

This supplement documents the four Elicit search routes used in the scoping review. It reproduces the research questions, screening criteria and data extraction instructions preserved in the original reports, and distinguishes automated platform outputs from subsequent human eligibility and evidence-role decisions.

The complete, unchanged PDF reports accompany the supplementary files in the Elicit_Reports folder of the submission archive. They contain the original flow diagrams, generated study tables, narrative summaries and reference lists. Those reports are historical AI-generated outputs; their inclusion decisions, study details and narrative conclusions should not be read as the final findings of this review. The human-verified evidence matrix is provided in Supplementary Material S6.

# Search dates and platform outputs

| **Route and purpose** | **Search date** | **Ranked records shown in report** | **Platform output** |
| --- | --- | --- | --- |
| ELICIT-01 Perinatal | 18 February 2026 | 1,000 | 31 structured extraction outputs |
| ELICIT-02 Older adults | 18 February 2026 | 1,000 | 17 structured extraction outputs |
| ELICIT-03 Broader question with youth filters | 18 February 2026 | 1,000 | 2 structured extraction outputs |
| ELICIT-04 Dedicated youth update | 31 July 2026 | 5,000 | 20 full-text candidates; 3 extraction proposals |

The search dates above were confirmed by the lead author. PDF CreationDate metadata fall on the same respective dates, but document PDF generation rather than independently establishing execution timestamps. The reports do not provide a separate visible search timestamp. Ranked and automatically screened records are not additional records independently screened by a human, and counts across routes are not deduplicated.

Automated screening in Elicit was a preliminary selection step. JCC made the final human eligibility decisions, including the 33 final full-text exclusions reported in the manuscript. No complete contemporaneous screening list was maintained. Supplementary Material S5 therefore provides the reported flow and the recoverable decisions, rather than a complete historical record of all exclusions.

# Original report provenance

**ELICIT-01.** Elicit - Access to Mental Health Care for Perinatal Women - Report.pdf

Search and screening: p. 3; extraction instructions: pp. 3–5.

**ELICIT-02.** Elicit - Access to Mental Health Care for Older Adults - Report.pdf

Search: pp. 2–3; screening: p. 3; extraction instructions: pp. 3–4.

**ELICIT-03.** Elicit - Patient-Centred Access in Mental Health Services - Report.pdf

Search: pp. 2–3; screening: p. 3; extraction instructions: pp. 3–4.

**ELICIT-04.** Elicit - Empirical Applications of Levesque Framework in Pe - Report(4).pdf

Search: p. 2; screening: p. 3; extraction instructions: pp. 3–5.

The following transcriptions retain the wording and scope of the original questions and instructions. PDF line wrapping, page breaks and word-break hyphens have been normalized for readability. The reports identify Elicit as the semantic retrieval platform and describe its coverage as including Semantic Scholar and OpenAlex. Backend model identifiers, the precise model versions and the numerical screening thresholds are not specified in these PDF reports.

The historical platform questions were not identical to the final human eligibility criteria. For example, ELICIT-01 allowed reference to the framework, whereas final direct evidence required substantive application; ELICIT-02 allowed samples with a majority aged 65 or older, whereas direct evidence required findings attributable to the prespecified population. ELICIT-03 combined a broad question with youth keyword filters. These original settings are retained here so that the retrieval process remains visible; they have not been retrospectively rewritten to match final decisions.

# ELICIT 01 Perinatal mental health care

Source: Elicit - Access to Mental Health Care for Perinatal Women - Report.pdf. Search and screening: p. 3; extraction instructions: pp. 3–5.

## Research question

Which empirical studies (2013–present) apply the Levesque et al. (2013) patient-centred access to health care framework to examine mental health care access among pregnant, perinatal, or postpartum women? Include quantitative, qualitative, or mixed-methods studies. The primary outcome must be mental health care access (e.g., access to psychiatric services, psychotherapy, counseling, perinatal mental health programs). For each study, report: Study design Country Population (pregnant, perinatal, postpartum) Type of mental health service How the Levesque framework is used Which access dimensions are examined Main findings related specifically to mental health care access Study design Population (pregnant, perinatal, postpartum) Mental health setting How the Levesque framework is used (citation only, conceptual framing, operationalization, measurement, intervention design, etc.) Which access dimensions (Approachability, Acceptability, Availability, Affordability, Appropriateness; Ability to perceive, seek, reach, pay, engage) are analyzed

No additional separately applied search filters are listed in this report.

## Automated screening criteria

We screened in sources based on their abstracts that met these criteria:

- Framework Application: Does this study explicitly apply, reference, or utilize the Levesque et al. (2013) patient-centred access to healthcare framework?
- Target Population: Does this study focus on pregnant women, women during the perinatal period, or postpartum women?
- Primary Outcome: Is mental health care access a primary outcome in this study (including access to psychiatric services, psychotherapy, counseling, or perinatal mental health programs)?
- Study Design: Is this an empirical study using quantitative, qualitative, or mixed-methods approaches, or a systematic review/meta-analysis?
- Publication Date: Was this study published in 2013 or later?
- Study Type Appropriateness: Is this study NOT a theoretical paper, commentary, editorial, opinion piece, case report, case series with fewer than 10 participants, or conference abstract without full-text availability?
- Access Focus: Does this study focus on access to mental health care rather than primarily evaluating treatment effectiveness or clinical outcomes?

Papers that failed any strict criterion were automatically excluded. For the remaining papers, we considered all screening questions together and made a holistic judgement about whether to screen in each paper.

## Data extraction instructions

We asked a large language model to extract each data column below from each paper. We gave the model the extraction instructions shown below for each column.

### Study Design

Extract study design and methodology, including:

- Research design (qualitative, quantitative, mixed-methods, systematic review, meta-analysis, etc.)
- Data collection methods
- Sample size
- Study timeframe

### Population Characteristics

Extract details about the study population, including:

- Specific perinatal stage (pregnant, perinatal, postpartum, or combination)
- Gestational timing if specified (e.g., first trimester, within 12 months postpartum)
- Any special population characteristics (e.g., refugees, adolescents, specific ethnic groups, high-risk)
- Geographic location/country

### Mental Health Services

Extract the type of mental health services or care being accessed, including:

- Specific services (psychiatric services, psychotherapy, counseling, perinatal mental health programs, screening, etc.)
- Service setting (primary care, specialty mental health, community-based, digital/telehealth)
- Level of care (prevention, treatment, ongoing support)

### Levesque Framework Application

Extract specifically how the Levesque et al. (2013) patient-centred access framework is used in the study, including:

- Citation only vs. conceptual framing vs. operationalization vs. measurement tool
- Whether used for data collection design, analysis framework, or interpretation
- How explicitly the framework guides the study methodology
- Any modifications or adaptations made to the original framework

### Framework Dimensions Examined

Extract which specific dimensions of the Levesque framework are analyzed, using the 10-dimension model: Supply-side: Approachability, Acceptability, Availability & Accommodation, Affordability, Appropriateness Demand-side: Ability to perceive, Ability to seek, Ability to reach, Ability to pay, Ability to engage

- List all dimensions explicitly studied
- Note if study focuses on subset or examines all dimensions
- Identify any dimensions that are primary vs. secondary focus

### Access Barriers

Extract barriers to mental health care access identified in the study, organized by Levesque framework dimensions where possible, including:

- Specific barriers within each framework dimension
- Whether barriers are individual, system-level, or societal
- Any barriers unique to the perinatal period or specific to mental health (vs. general healthcare) access

### Access Facilitators

Extract facilitators or enablers of mental health care access identified in the study, including:

- Specific facilitators within each framework dimension
- Strategies or interventions that improve access
- System-level or policy solutions identified
- Any facilitators specific to perinatal mental health care

### Key Findings

Extract the main findings specifically related to mental health care access among pregnant, perinatal, or postpartum women, including:

- Primary conclusions about access patterns or problems
- Quantitative results (if applicable) such as access rates, effect sizes, statistical significance
- Qualitative themes or insights about access experiences
- Recommendations for improving access
- Any findings that advance understanding of the Levesque framework in this context

# ELICIT 02 Mental health care for older adults

Source: Elicit - Access to Mental Health Care for Older Adults - Report.pdf. Search: pp. 2–3; screening: p. 3; extraction instructions: pp. 3–4.

## Research question

Which empirical studies (2013–present) apply the Levesque et al. (2013) patient-centred access to health care framework to examine mental health care access among older adults (aged 65 years and older)? Include quantitative, qualitative, or mixed-methods studies. The primary outcome must be mental health care access (e.g., access to psychiatric services, psychotherapy, counseling, geriatric mental health services, or community mental health programs). For each study, report: Study design Country Population characteristics (age range, mean age) Type of mental health service How the Levesque framework is used Which access dimensions are examined Main findings specifically related to mental health care access

No additional separately applied search filters are listed in this report.

## Automated screening criteria

We screened in sources based on their abstracts that met these criteria:

- Target Population Age: Does this study focus on older adults aged 65 years and older, OR does it report results separately for participants ≥65 years, OR do older adults (≥65 years) comprise the majority (≥50%) of the study population?
- Levesque Framework Application: Does this study explicitly reference and apply the Levesque et al. (2013) patient-centred access to healthcare framework as a theoretical foundation, analytical framework, or organizing structure?
- Mental Health Care Access Focus: Does this study examine access to mental health services as a primary focus (including psychiatric services, psychotherapy, counseling, geriatric mental health services, community mental health programs, or other mental health interventions)?
- Study Design and Data Type: Is this study an empirical study using quantitative, qualitative, or mixed-methods approaches with original data, OR a systematic review/meta-analysis that synthesizes empirical studies?
- Publication Date: Was this study published in 2013 or later?
- Access vs. Intervention Focus: Is this study primarily focused on examining access to mental health care rather than solely evaluating the effectiveness of mental health interventions?

Papers that failed any strict criterion were automatically excluded. For the remaining papers, we considered all screening questions together and made a holistic judgement about whether to screen in each paper.

## Data extraction instructions

We asked a large language model to extract each data column below from each paper. We gave the model the extraction instructions shown below for each column.

### Study Details

Extract basic study information to confirm eligibility and provide context, including:

- Study design (quantitative, qualitative, mixed-methods)
- Country where study was conducted
- Year of publication
- Study time period/data collection dates
- Sample size

### Older Adult Population

Extract population characteristics specifically focusing on older adults (65+ years), including:

- Age range of participants
- Mean age (if provided)
- Percentage of sample that is 65+ years old
- Other relevant demographic characteristics (gender, ethnicity, socioeconomic status, rural/urban)
- Any specific older adult subgroups studied (e.g., community-dwelling, nursing home residents, those with dementia)

### Mental Health Service Type

Extract details about the specific mental health services or access issues examined in the study, including:

- Type of mental health service (psychiatric services, psychotherapy, counseling, geriatric mental health services, community mental health programs, etc.)
- Service setting (primary care, specialized mental health facilities, community-based, telehealth/virtual)
- Specific mental health conditions addressed (depression, anxiety, dementia-related behavioral issues, etc.)
- Level of care (prevention, screening, treatment, ongoing care)

### Levesque Framework Application

Extract how the Levesque et al. (2013) patient-centred access framework is applied in the study, including:

- Whether framework was used a priori (to design study/tools) or a posteriori (to organize/analyze data)
- Which of the 5 access dimensions are examined (approachability, acceptability, availability and accommodation, affordability, appropriateness)
- Which of the 5 population abilities are examined (ability to perceive, ability to seek, ability to reach, ability to pay, ability to engage)
- How the framework guided data collection, analysis, or interpretation
- Any modifications or adaptations made to the framework

### Mental Health Access Findings

Extract main findings specifically related to mental health care access among older adults, including:

- Key barriers to mental health access identified for older adults
- Facilitators or enablers of mental health access for this population
- Access outcomes or measures reported (utilization rates, unmet need, delays in care, satisfaction)
- Differences in access across older adult subgroups
- Policy or practice implications specifically for improving mental health access among older adults
- Any quantitative results (effect sizes, odds ratios, percentages) related to access

# ELICIT 03 Broader mental health query with youth filters

Source: Elicit - Patient-Centred Access in Mental Health Services - Report.pdf. Search: pp. 2–3; screening: p. 3; extraction instructions: pp. 3–4.

## Research question

Find studies that cite or apply the Levesque et al. (2013) patient-centred access to health care framework in the context of mental health services. Include conceptual, empirical, and implementation studies (2013–present). Provide details on how the framework is used and which access dimensions are operationalized. Are there studies applying the Levesque et al. (2013) access to healthcare framework to mental health services for children, adolescents, perinatal women, or older adults? Include empirical or conceptual studies (2013–present).

## Additional filters

We applied the following filters: published after 2013; including keywords: children, adolescents.

The report uses both “published after 2013” for the filter and “2013 or later” in the screening question below. The PDF does not resolve this difference in the technical date boundary. The research question above includes the second population-specific sentence shown in the report’s Paper search section; the shorter title on its first page does not contain that sentence.

## Automated screening criteria

We screened in sources based on their abstracts that met these criteria:

- Levesque Framework Citation: Does this study explicitly cite, reference, or apply the Levesque et al. (2013) patient-centred access to healthcare framework?
- Mental Health Context: Is this study conducted within mental health services, programs, or populations?
- Framework Operationalization: Does this study provide information about how the Levesque framework is used or which access dimensions are operationalized?
- Publication Date: Was this study published in 2013 or later?
- Substantive Framework Application: Does this study demonstrate meaningful engagement with the Levesque framework beyond mere passing reference (i.e., includes substantive application, analysis, or discussion of its components)?
- Study Design and Rigor: Is this study a conceptual, empirical, or implementation study with appropriate methodological rigor or significant conceptual contribution (e.g., theoretical papers, quantitative/qualitative research, mixed-methods studies, case studies, program evaluations, systematic reviews, meta-analyses)?

Papers that failed any strict criterion were automatically excluded. For the remaining papers, we considered all screening questions together and made a holistic judgement about whether to screen in each paper.

## Data extraction instructions

We asked a large language model to extract each data column below from each paper. We gave the model the extraction instructions shown below for each column.

### Study Type

Extract the type of study and research approach used, specifically noting:

- Study design (conceptual analysis, cross-sectional survey, longitudinal study, intervention trial, implementation study, etc.)
- Whether it's conceptual, empirical, or implementation research
- Research methodology (quantitative, qualitative, mixed methods)
- Data collection approach if empirical

### Mental Health Context

Extract details about the mental health context where the Levesque framework is being applied, including:

- Target population (age group, specific mental health conditions, demographics)
- Mental health service setting (primary care, specialty mental health, community services, schools, etc.)
- Geographic location and health system context
- Specific mental health issues addressed (ADHD, depression, anxiety, psychosis, etc.)

### Framework Application

Extract exactly how the Levesque et al. (2013) patient-centred access framework is being used in the study, including:

- Primary purpose (conceptual model, measurement tool, intervention design guide, evaluation framework, etc.)
- Whether it's the main theoretical framework or one of several cited
- Any modifications or adaptations made to the original framework
- How it's integrated with other models or frameworks
- Specific tools or instruments developed based on the framework

### Access Dimensions

Extract which specific access dimensions from the Levesque framework are operationalized or discussed, noting:

- Supply-side dimensions: Approachability, Acceptability, Availability/Accommodation, Affordability, Appropriateness
- Demand-side abilities: Ability to perceive, Ability to seek, Ability to reach, Ability to pay, Ability to engage
- How each dimension is defined or measured in the mental health context
- Any dimensions that are explicitly excluded or not addressed
- Novel operationalizations specific to mental health services

### Access Findings

Extract key findings related to mental health service access using the Levesque framework, including:

- Main barriers and facilitators identified for each access dimension
- Quantitative results (timing, rates, disparities) if available
- Qualitative insights about access experiences
- Differences across population subgroups
- Recommendations for improving access based on framework application
- How findings contribute to understanding mental health service access

### Implementation Details

If the study involves implementation or intervention based on the Levesque framework, extract:

- What specific interventions or changes were implemented to improve access
- Which access dimensions the implementation targeted
- Implementation setting and duration
- Implementation outcomes or process measures
- Barriers and facilitators to implementing framework-based changes
- Sustainability considerations
- If not an implementation study, note 'Not applicable'

# ELICIT 04 Dedicated child and adolescent update

Source: Elicit - Empirical Applications of Levesque Framework in Pe - Report(4).pdf. Search: p. 2; screening: p. 3; extraction instructions: pp. 3–5.

## Research question

Which empirical studies and evidence syntheses published from 2013 onward have substantively applied the Levesque et al. (2013) patient-centred access to health care framework to access to, utilization of, or engagement with mental health services, mental-health diagnostic pathways, or treatment pathways among children and adolescents aged 0–17 years?

No additional separately applied search filters are listed in this report.

## Automated screening criteria

The same six questions below appear in both the abstract-screening and full-text-screening sections of the report.

We screened in sources based on their abstracts that met these criteria:

- Population Scope: Does the study focus on children/adolescents aged 0-17 years, OR does it include mixed-age populations with separately reported findings for the 0-17 age group?
- Mental Health Focus: Is access to, utilization of, or engagement with mental health services, mental health diagnostic pathways, or treatment pathways a primary or substantial focus of the study?
- Framework Application: Does the study explicitly and substantively apply the Levesque et al. (2013) patient-centred access framework to inform study design, research questions, data collection, measurement, analysis, coding, or interpretation (beyond merely citing it)?
- Study Type: Is this a completed primary empirical study (qualitative, quantitative, or mixed-methods) OR an evidence synthesis (systematic review, scoping review, or mapping review)?
- Publication Date: Was the study published in 2013 or later?
- Data Completeness: Is this a completed study with sufficient data (i.e., NOT an ongoing study, conference abstract without full publication, or study with incomplete data)?

**Abstract-stage decision rule.** Papers that failed any strict criterion were automatically excluded. For the remaining papers, we considered all screening questions together and made a holistic judgement about whether to screen in each paper.

**Full-text-stage decision rule.** Papers that failed any strict criterion were automatically excluded. For the remaining papers, we considered all screening questions together and made a holistic judgement about whether to include each paper in the final analysis.

## Data extraction instructions

We asked a large language model to extract each data column below from each paper. We gave the model the extraction instructions shown below for each column.

### Framework Application

Extract detailed information about how the Levesque et al. (2013) patient-centred access framework was substantively applied in this study, including:

- Specific ways the framework informed study design, research questions, data collection, measurement, analysis, coding, or interpretation
- Whether framework dimensions were used as organizing themes, coding categories, measurement domains, or analytical structure
- How the framework went beyond a passing citation to actually shape the research approach
- Any adaptations or modifications made to the framework for child/adolescent populations

### Population Characteristics

Extract details about the child and adolescent population studied (aged 0-17 years), including:

- Age range and mean age
- Sample size for participants aged 0-17 years
- Demographic characteristics (gender, ethnicity, socioeconomic status)
- Clinical characteristics (mental health conditions, severity)
- Whether findings for 0-17 year olds were reported separately from other age groups
- Geographic location and setting (urban/rural, country/region)

### Mental Health Focus

Extract information about the specific mental health services, diagnostic pathways, or treatment pathways that were the focus regarding access, utilization, or engagement, including:

- Type of mental health services (e.g., specialized services, primary care, school-based, inpatient, outpatient)
- Specific mental health conditions addressed (e.g., ADHD, depression, anxiety, general mental health)
- Whether focus was on diagnostic pathways, treatment pathways, or general service access
- Care settings and service delivery models examined

### Study Design

Extract the study design and methodology, specifying:

- Type of empirical study (qualitative, quantitative, mixed-methods primary study) or evidence synthesis (systematic review, scoping review, mapping review)
- Specific research design (e.g., cross-sectional survey, cohort study, RCT, thematic synthesis)
- Data collection methods used
- Sample size and sampling approach
- Time period of data collection

### Levesque Framework Dimensions

Extract which specific dimensions of the Levesque framework were addressed or applied in this study, including:

- Approachability (and ability to perceive)
- Acceptability (and ability to seek)
- Availability and accommodation (and ability to reach)
- Affordability (and ability to pay)
- Appropriateness (and ability to engage)
- Whether all five dimensions were addressed or only specific ones
- How each dimension was operationalized or measured in the child/adolescent context

### Access Barriers

Extract findings about barriers to access, utilization, or engagement with mental health services among children and adolescents aged 0-17 years, including:

- Specific barriers identified within each Levesque framework dimension
- Barriers unique to child/adolescent populations
- Systemic, provider, or individual-level barriers
- How barriers differed by age, gender, condition, or other characteristics within the 0-17 age range

### Access Facilitators

Extract findings about facilitators or enablers of access, utilization, or engagement with mental health services among children and adolescents aged 0-17 years, including:

- Specific facilitators identified within each Levesque framework dimension
- Facilitators unique to child/adolescent populations
- Systemic, provider, or individual-level facilitators
- How facilitators differed by age, gender, condition, or other characteristics within the 0-17 age range

### Key Access Findings

Extract the main findings related to access, utilization, or engagement with mental health services among children and adolescents aged 0-17 years, including:

- Quantitative results (rates, odds ratios, effect sizes) related to access or utilization
- Qualitative themes about access experiences
- Time to access or diagnosis for different pathways
- Disparities in access by demographic characteristics
- Service utilization patterns and engagement rates
- How findings specifically relate to the child/adolescent population

# ELICIT 04 platform flow and human adjudication

The original youth report selected 20 candidates for its full-text stage and proposed three sources for extraction. Its automated exclusions and missing-full-text flags describe platform processing and are separate from JCC’s human selection decisions.

| **Platform stage or reason** | **Count** |
| --- | --- |
| Ranked records entering automated abstract screening | 5,000 |
| Excluded at automated abstract screening | 4,980 |
| Population scope / mental health focus / framework application | 1,045 / 270 / 3,029 |
| Study type / data completeness / other or below threshold | 363 / 271 / 2 |
| Candidates entering automated full-text screening | 20 |
| Removed at the platform full-text stage | 17 |
| Population scope / framework application / no platform full text | 6 / 1 / 10 |
| Sources proposed for extraction | 3 |

All 20 ELICIT-04 candidates contributed to the reported human screening pool, not only the three extraction proposals. The three extraction proposals did not constitute three new direct-evidence inclusions. Martínez-Jaime et al. was already represented in the review; the two proposed reviews were retained as adjacent evidence after human assessment. The historical report identifies Werlen et al. as 2019; the verified publication year used in the review is 2020.

## Human follow-up of the ten records without platform full text

After title and abstract assessment, JCC retrieved and assessed six of these ten records: three were retained as adjacent evidence and three were excluded after full-text assessment. The remaining four were excluded at the human title/abstract stage and were not sought for retrieval. Their individual titles or identifiers are not recoverable from the retained documentation. No reports sought in this human follow-up remained unretrieved, and none of the six retrieved reports was added as direct evidence.

The record-level decisions recoverable for this route are shown below. This table is a retrospective account of these decisions, not a complete contemporaneous screening log.

| **Record** | **Platform status and human decision** | **Basis of human decision** |
| --- | --- | --- |
| Martínez-Jaime et al. (2024) | Extraction proposal; direct empirical evidence already included | Population aged 0–17; ADHD diagnostic pathway; substantive Levesque operationalization. |
| Werlen et al. (2020) | Extraction proposal; adjacent review evidence | Eligibility extended to participants under 19 and mixed samples; no separate synthesis restricted to ages 0–17. |
| Barbo and Petrucka (2024) | Extraction proposal; adjacent review evidence | Youth definition extended beyond the prespecified population aged 0–17. |
| Fathi Afshar (2019) | Initially no platform full text; retrieved; adjacent empirical evidence | Levesque application to general health care access among refugee minors; mental health access was not the primary focus. |
| Williams-Ridgway et al. (2026) | Initially no platform full text; retrieved; adjacent empirical evidence | Mental health application with a population aged 10–25 and no separate findings for ages 0–17. The same source is PMID 41709297, also identified by the PubMed update on 28 July 2026; it is counted once in the final map. |
| Yılmaz et al. (2025) | Initially no platform full text; retrieved; adjacent review evidence | Overview included youth up to approximately age 24 without consistently separate findings for ages 0–17. |
| Audet and Drolet (2014) | Initially no platform full text; retrieved; excluded | French-language publication and no application of the Levesque et al. (2013) framework. |
| Garces Dávila (2017) | Initially no platform full text; retrieved; excluded | Population aged 15–24 without separate results for ages 0–17; CSDH framework rather than Levesque. |
| Dumke et al. (2024) | Initially no platform full text; retrieved; excluded | Adult refugee and asylum-seeker population; child and youth reviews explicitly excluded. |
| Four further records | Initially no platform full text; excluded at human title/abstract stage; not sought | Did not meet the criteria to proceed to retrieval; individual identifiers were not retained. |

Final study classifications, extracted data and evidence roles are reported in Supplementary Material S6. Differences from the generated reports reflect human verification of the original publications; the unchanged PDFs preserve the initial automated output for auditability.
