# Supplementary material for: Patient-centered access to mental health care at selected life-course stages: a scoping review using the Levesque framework
Source: BMC Health Serv Res. 2026 Oct 22;26:1464. doi: 10.1186/s12913-026-15895-z (PMC13693167; doi:10.1186/s12913-026-15895-z)
Supplement: Supplementary file 11 [file 12913_2026_15895_MOESM8_ESM.docx]

Supplementary Material S5 Search routes screening decisions and incremental yield

**Patient-centered access to mental health care at selected life-course stages: a scoping review using the Levesque framework**

Search documentation and available screening decisions aligned with manuscript V2.14, including the PubMed searches of 28 July 2026 and Web of Science search history for 1 August 2026

# S5.1 Identification routes and platform outputs

| **Identification route** | **Tool/database** | **Date** | **Query/seed** | **Platform or route-level output** | **Transferred to human verification** | **Incremental yield** |
| --- | --- | --- | --- | --- | --- | --- |
| Original perinatal Elicit report | Elicit | 18 Feb 2026 | Population-specific semantic search; exact question and screening instructions in S4, ELICIT-01 | Up to 1,000 ranked records | 31 structured extraction outputs | Contributed to the original 27-source evidence set; source-level routes shown in S6 |
| Original older-adult Elicit report | Elicit | 18 Feb 2026 | Population-specific semantic search; exact question and screening instructions in S4, ELICIT-02 | Up to 1,000 ranked records | 17 structured extraction outputs | Contributed to the original 27-source evidence set; source-level routes shown in S6 |
| Original broader mental-health Elicit report | Elicit | 18 Feb 2026 | Broader mental-health/Levesque search with children/adolescents filter; exact question and instructions in S4, ELICIT-03 | Up to 1,000 ranked records | 2 structured extraction outputs | Contributed to the original 27-source evidence set; source-level routes shown in S6 |
| Thematic PubMed update | PubMed | 28 Jul 2026 | Complete framework-focused MeSH and title/abstract query with Create Date restriction; exact executed string in S5.7 | 2 records | 2 records screened | 0 direct; 1 adjacent source in the final map (Williams-Ridgway, also ELICIT-04/citation); 1 excluded protocol. Shared yield reported in S5.5. |
| Forward citation lookup of Levesque et al. (2013) | PubMed, Cited by | 28 Jul 2026 | Seed: Levesque et al. (2013), DOI 10.1186/1475-9276-12-18; Cited by opened (author confirmed) | Total citing-list size and filters, if any, not documented | No additional candidate records contributed (author report); total examined not documented | No additional candidate contribution reported; not a claim of an empty citing list |
| Dedicated youth-focused Elicit report (ELICIT-04) | Elicit | 31 Jul 2026 | Exact question reproduced in S4 | 5,000 ranked records | 20 Elicit full-text candidates; 3 extraction proposals | 5 adjacent sources retained, including Williams-Ridgway also retrieved by PubMed; not an exclusive incremental count. Martínez-Jaime was already in the original set. |
| Forward citation tracking of Levesque et al. (2013) | Web of Science Core Collection | 1 Aug 2026 | Seed DOI 10.1186/1475-9276-12-18; complete citation filters in S5.8 | 2,423 Web of Science citing records | 185 youth + 48 perinatal + 67 older-adult hits = 300 route-specific hits before cross-filter deduplication | 5 direct sources reported for citation follow-up (S5.5); complete database provenance not documented |

Platform and route-hit counts are not counts of unique records independently screened by a human reviewer. Human flow totals are reported aggregates without a complete record-level log. PubMed Cited by contributed no additional candidates according to the author; its total list size and number examined are not documented.

Figure 1 adds 50 + 20 + 300 + 2 = 372 route entries. The three youth extraction proposals are nested within the 20 candidates and are not added again. This arithmetic does not independently verify the reported five duplicate removals or establish the screening-stage provenance of every retained source, including the predefined framework seed. A complete record-level reconciliation is unavailable.

# S5.2 Reported human screening and eligibility flow

| **Stage** | **n** | **Operational note** |
| --- | --- | --- |
| Candidate-record entries merged before deduplication | 372 | 50 original Elicit outputs + 20 ELICIT-04 candidates + 300 Web of Science citation-filter hits + 2 thematic PubMed records; no additional Cited by candidates reported |
| Duplicates removed across and within routes | 5 | Difference between reported entries before and after deduplication; exact historical software and matching workflow not documented |
| Unique records screened by title and abstract | 367 | Human screening by JCC |
| Records excluded at title/abstract stage | 297 | Did not proceed to retrieval |
| Reports sought for retrieval | 70 | All potentially eligible records after title/abstract screening |
| Reports not retrieved | 0 | All reports sought were obtained |
| Full-text reports assessed | 70 | Assessment against the original publications |
| Reports excluded after full-text assessment | 33 | JCC applied the eligibility criteria; 33 human exclusions confirmed; no complete article-level log was maintained |
| Sources retained in the full evidence map | 37 | 8 direct empirical; 2 direct review-level; 22 adjacent empirical/review-level; 2 contextual reviews; 1 protocol; 2 conceptual/framework-level |

# S5.3 Population-specific forward-citation filters

| **Population filter** | **Hits** | **Documented conceptual content** | **Verbatim query status** |
| --- | --- | --- | --- |
| Youth | 185 | Children/adolescents/youth terms combined with mental-health terms within the citing-document set | 1 Aug 2026, 17:49: complete citation-filter query in S5.8; author-supplied completion, 24 Sep 2026 |
| Perinatal | 48 | Pregnancy/perinatal/postpartum/maternal terms combined with mental-health terms | 1 Aug 2026, 19:48: complete citation-filter query in S5.8; historical PDF |
| Older adults | 67 | Older-adult/geriatric/ageing terms combined with mental-health terms | 1 Aug 2026, 19:59: complete citation-filter query in S5.8; confirmed by author, 24 Sep 2026 |
| Combined route-specific hits | 300 | Before cross-filter deduplication; individual records may occur in more than one population filter | Not a unique-record count |

# S5.4 Available source-specific decisions: partial retrospective account

The 35 unique named sources below comprise 26 excluded and 9 retained sources documented retrospectively. The former standalone entry for PMID 41709297 has been consolidated with Williams-Ridgway et al.; these were the same source, not two different studies. They are not a complete historical screening log and cannot be mapped exhaustively to the reported 367 screened records or 33 full-text exclusions. Only three entries explicitly identify exclusion after full-text assessment. General exclusion labels do not establish the screening stage; no exclusion-reason frequencies are derived from this subset.

| **Record/title** | **Identification route(s)** | **Final decision** | **Primary reason or eligibility basis** |
| --- | --- | --- | --- |
| Healthcare professionals’ perspectives on minoritised ethnic young people’s access to eating disorder services in the West Midlands, United Kingdom: a qualitative study [Williams-Ridgway et al. 2026; PMID 41709297] | Thematic PubMed update, 28 Jul 2026; ELICIT-04 human follow-up; forward citation | Adjacent empirical; retained | Substantive Levesque application; mixed target age 10–25 years without separate 0–17 findings. Retained as adjacent, not direct. The prior separate PMID entry referred to this same source and has been consolidated. |
| Treatment Barriers and Modality Preferences Among Individuals With Obsessive-Compulsive and Related Disorders | Youth filter | Excluded | Not youth-specific and no substantive Levesque application established |
| Examining factors influencing forcibly displaced children and youth’s access to mental health support: An overview of reviews | Youth; ELICIT-04 follow-up; forward citation | Adjacent review-level; retained | Age range extended to approximately 24 years; no consistently separate 0–17 synthesis |
| Challenges in Accessibility of Public Specialized Mental Health Services for Children and Adolescents in Mexico | Youth filter | Excluded | No substantive Levesque-based design, analysis, or interpretation established |
| Access to mental health care for Black and Latino teens with anxiety and depression: a qualitative study | Youth forward citation | Direct empirical; retained | Meets population, mental-health-access, completed-study, and substantive-framework-use criteria |
| Barriers to seeking consultation at public service and medical facilities among pregnant and postpartum women in Japan: a cross-sectional study | Youth filter; reassigned to perinatal screening | Excluded | Wrong youth filter and no substantive Levesque application established |
| Challenges of Accessibility: Experience of Receiving Ukrainian War Refugees With Disabilities in Poland and Romania | Youth filter | Excluded | Mixed population and broad support/service access rather than target-population mental-health access |
| Closing the gap in access to child mental health care: provider feedback from the Wisconsin Child Psychiatry Consultation Program | Youth forward citation | Direct empirical; retained | Target-population access was the explicit unit of analysis; substantive framework use |
| Understanding the factors related to how East and Southeast Asian immigrant youth and families access mental health and substance use services: A scoping review | Youth filter | Excluded | Different analytical framework; no substantive Levesque application |
| Mental health service accessibility for young people in rural, regional, and remote areas and its implications for social work practice: a scoping review protocol | Youth filter | Excluded | Protocol without completed findings |
| Access to primary care for children and young people (CYP) in the UK: a scoping review of CYP’s, caregivers’ and healthcare professionals’ views and experiences of facilitators and barriers | Youth filter | Excluded | General primary-care access rather than mental-health access; mixed age range |
| Mental health needs and accessing specialised healthcare in Mexican children with mental disorders: gender- and diagnosis-dependent differences | Youth filter | Excluded | Levesque used only as background/passing citation |
| Differences in postpartum mental healthcare among women with identified needs: The role of migration status | Youth and perinatal filters | Excluded | Perinatal duplicate across filters; no substantive Levesque application |
| A scoping review of the barriers and facilitators to accessing and utilising mental health services across regional, rural, and remote Australia | Youth filter | Excluded | All-age evidence without separate 0–17 synthesis; no substantive Levesque-based synthesis established |
| Availability and Accessibility of Mental Health Services for Youth: A Descriptive Survey of Safety-Net Health Centers During the COVID-19 Pandemic | Youth forward citation | Direct empirical; retained | Target-population service access and substantive use of the patient-centered access framework |
| Accessing mental health services for a child living with anxiety: Parents’ lived experience and recommendations | Youth filter | Excluded after full-text assessment | Mixed age range 10–22 years without separate 0–17 findings; not retained as adjacent because no distinct additional contribution was required |
| Access to mental health and addiction services for youth and their families in Ontario: perspectives of parents, youth, and service providers | Youth filter | Excluded after full-text assessment | Population aged 16–24 years without separate findings for 16–17-year-olds; not retained as adjacent |
| Facilitating factors for seeking help for mental health problems among Norwegian adolescent males: A focus group interview study | Youth filter | Excluded | No substantive Levesque application |
| Mental health and psychosocial support services in primary health care in Nepal: perceived facilitating factors, barriers and strategies for improvement | Youth filter | Excluded | No youth-specific target population and no substantive Levesque application |
| Interventions to improve children’s access to mental health care: a systematic review and meta-analysis | Youth; ELICIT-04; forward citation | Adjacent review-level; retained | Eligibility extended to under 19/mixed samples rather than the prespecified 0–17 population |
| Healthcare professionals’ prioritisation of barriers to accessing evidence-based psychological therapy for perinatal obsessive-compulsive disorder | Perinatal filter | Excluded | Framework use was contextual/post hoc rather than a substantive study-design or analytical application |
| Barriers and facilitators of accessing perinatal mental health services: The perspectives of women receiving continuity of care midwifery | Perinatal forward citation | Direct empirical; retained | Meets all direct criteria |
| Identification of mothers with mental health problems is accidental: perceptions of health care providers on availability, access, and support for maternal mental health care for adolescent mothers in Malawi | Perinatal filter | Excluded | No substantive Levesque application established |
| Barriers to perinatal mental health care experiences by midwives and obstetricians and their patients: A rapid review | Perinatal forward citation | Direct review-level; retained | Completed review and substantive mapping to Levesque dimensions |
| Barriers and facilitators to housing and healthcare services for people experiencing homelessness with concurrent brain injury, mental health and substance use disorders: a qualitative study | Older-adult filter | Excluded | No prespecified older-adult population |
| Mental health and aging in New Zealand: mixed-methods analysis of experiences of healthcare from a survey of older adults with mental health conditions | Older-adult filter | Excluded after full-text assessment | Sample began at age 55 and findings for adults aged 65+ were not separately attributable |
| Does Maintaining High Accessibility to Medical Care Services Increase Psychological Well-Being of Chinese Older Adults? | Older-adult filter | Excluded | General medical access with psychological well-being as outcome, not mental-health-service access |
| Self-identified barriers to rural mental health services in Iowa by older adults with multiple comorbidities: qualitative interview study | Older-adult filter | Excluded | Used a different access framework rather than substantive Levesque application |
| Help-seeking experiences of older adults with a diagnosis of moderate depression | Older-adult filter | Excluded | No substantive Levesque application |
| An integrative review of potential enablers and barriers to accessing mental health services in Ghana | Older-adult filter | Excluded | Not specific to older adults and no substantive Levesque-based synthesis established |
| Les services en santé mentale pour les jeunes francophones de la région d’Ottawa | ELICIT-04 human follow-up | Excluded | French-language publication; cited a different Levesque source from 2005 rather than the 2013 framework |
| Individual and Ecological Determinants of Access to Mental Health Services Among Youth: An Analysis of Survey and Census Data | ELICIT-04 human follow-up | Excluded | Population aged 15–24 without separate 0–17 findings; CSDH rather than Levesque framework |
| Barriers to accessing mental health care for refugees and asylum seekers in high-income countries: A scoping review of reviews mapping demand and supply-side factors onto a conceptual framework | ELICIT-04 human follow-up | Excluded | Adult refugee/asylum-seeker population; children and youth explicitly excluded |
| Access to Health Care for Refugee Children and Unaccompanied Minor Aliens in The Netherlands: A Qualitative Study | ELICIT-04 human follow-up | Adjacent empirical; retained | Substantive Levesque application but general health-care access rather than mental-health-specific access |
| Ha et al. (2025). ‘Making Moves’: Protocol for a hybrid effectiveness-implementation pre-post trial of a co-designed online physical activity program for childhood cancer survivors. PMID 41878391; DOI 10.1016/j.jsampl.2025.100099. | Targeted PubMed update, 28 Jul 2026 | Excluded; not retained | Protocol for a physical-activity intervention, not a mental-health-service-access study; no substantive Levesque application or completed empirical findings for the proposed trial. |

Four ELICIT-04 records without documented identifiers: The Elicit report lists ten records without platform full-text access. According to the author, six were manually retrieved and four were excluded after human title/abstract review. The report does not identify these records individually, and no complete human screening list was maintained; the four excluded titles cannot be recovered from the supplied materials.

# S5.5 Incremental retained sources across update routes

The combined update yield is 27 original sources + five adjacent sources from the overlapping PubMed/youth Elicit follow-up + five direct sources from citation follow-up = 37. This identifies additions to the evidence map, not the chronology of every eligibility decision. Thematic PubMed retrieval alone produced no direct source; one retrieved source is present as adjacent evidence in the final map.

| **Route stage** | **Source** | **Added n** | **Incremental retained sources / note** |
| --- | --- | --- | --- |
| Original evidence set | Three original Elicit reports plus predefined framework/context sources | 27 | Original 27-source map retained after re-adjudication |
| PubMed update and dedicated youth Elicit follow-up (combined) | PubMed, 28 Jul 2026; ELICIT-04, 31 Jul 2026, including human follow-up | 5 | Five adjacent sources beyond the original map: Werlen; Barbo & Petrucka; Fathi Afshar; Williams-Ridgway; Yılmaz. Williams-Ridgway was retrieved in both routes; it is counted once. Martínez-Jaime was already in the original map. An exclusive route-specific incremental attribution is not claimed. |
| Combined citation follow-up | Web of Science, 1 Aug 2026; PubMed Cited by, 28 Jul 2026 (no additional candidates reported) | 5 | Viveiros & Darling 2018; Marshman et al. 2023; Daskalska et al. 2025; Daskalska et al. 2024; Adams 2024. The previously combined yield is retained; complete individual database provenance was not documented. |
| Final evidence map | All routes after deduplication and human adjudication | 37 | 23 empirical primary studies; 11 reviews/evidence syntheses; 1 protocol; 2 conceptual/framework sources |

Source-identity correction (1 October 2026): Williams-Ridgway et al., DOI 10.1186/s40337-026-01546-5, explicitly used the framework to inform the interview schedule. Ha et al., DOI 10.1016/j.jsampl.2025.100099, describes a physical-activity trial protocol. This correction aligns the final source roles; it is not a new literature search.

# S5.6 Documentation limitations of screening records

The author confirmed that four criteria governed Elicit’s automated preliminary screening: presence of the Levesque framework, data completeness, mental-health focus, and fit with the age/target group. JCC assessed the full texts and made the 33 final human exclusion decisions. The platform criteria do not assign article-level reasons or new exclusion frequencies to those 33 reports.

In the youth Elicit report, data completeness meant a completed study with sufficient data, excluding ongoing studies, conference abstracts without a full publication, and studies with incomplete data. This historical platform definition does not establish a zero-missing-data requirement or specify the reasons for the 33 human exclusions.

The author confirmed opening PubMed Cited by for Levesque et al. (2013) on 28 July 2026 and reported no additional candidate records. This does not establish an empty citing list or zero records examined; those historical totals, any filters, and record-level overlap are not documented.

JCC reviewed articles and excluded those that did not meet the eligibility criteria, but did not maintain a complete screening list. The human flow totals are retained as reported aggregates and cannot be independently checked against an exhaustive contemporaneous record-level log. S5.4 provides only the available retrospective subset.

The 33 final human full-text exclusions are author-confirmed. A complete title-by-title list with individual primary reasons is unavailable; the four automated Elicit criteria do not supply those reasons, and reason-specific exclusion frequencies cannot be reconstructed.

# S5.7 Executed thematic PubMed update search

The thematic framework-focused query below was executed in PubMed on 28 July 2026. It retrieved two records, neither eligible as direct evidence. PMID 41709297 is Williams-Ridgway et al., retained as adjacent evidence in the final map and also identified via ELICIT-04/citation; PMID 41878391 was excluded. The former blanket statement that both lacked substantive framework use has been corrected. These two records belong to the thematic query, separately from the Cited by lookup confirmed for the same date. Earlier query variants were preparatory formulations and were not separate identification routes.

Create Date [crdt] identifies when a citation record was first created in PubMed; it does not define publication-date eligibility. The expression 2026/02/18:3000[crdt] is reproduced unchanged. Because its upper date bound is open, the reported two-record output is anchored to the historical execution on 28 July 2026 and should not be interpreted as a current search result.

Source for field definitions: National Library of Medicine. PubMed User Guide, Create Date [crdt] and search field tags. https://pubmed.ncbi.nlm.nih.gov/help/ (accessed 24 September 2026).

## Full query executed on 28 July 2026

Framework, mental-health, access, and population blocks are combined with the Create Date restriction. Syntax is unchanged; whitespace is normalized for readability.

( ( Levesque[tiab] OR "Levesque framework"[tiab] OR "Levesque model"[tiab] OR "patient-centred access to health care"[tiab] OR "patient-centered access to health care"[tiab] OR "patient-centred access to healthcare"[tiab] OR "patient-centered access to healthcare"[tiab] OR "patient-centred access framework"[tiab] OR "patient-centered access framework"[tiab] OR "patient-centred healthcare access framework"[tiab] OR "patient-centered healthcare access framework"[tiab] OR "five dimensions of accessibility"[tiab] OR "five abilities of populations"[tiab] OR ( ( approachability[tiab] OR acceptability[tiab] OR "availability and accommodation"[tiab] OR affordability[tiab] OR appropriateness[tiab] ) AND ( "ability to perceive"[tiab] OR "ability to seek"[tiab] OR "ability to reach"[tiab] OR "ability to pay"[tiab] OR "ability to engage"[tiab] ) ) ) AND ( "Mental Health Services"[Mesh] OR "Community Mental Health Services"[Mesh] OR "Mental Disorders"[Mesh] OR "mental health"[tiab] OR "mental healthcare"[tiab] OR "mental health care"[tiab] OR "mental health service*"[tiab] OR "mental illness*"[tiab] OR "mental disorder*"[tiab] OR psychiatr*[tiab] OR psychotherap*[tiab] OR "psychological care"[tiab] OR "psychological service*"[tiab] OR "psychological treatment*"[tiab] OR "behavioral health"[tiab] OR "behavioural health"[tiab] OR depress*[tiab] OR anxi*[tiab] OR psychosis[tiab] OR psychotic[tiab] OR PTSD[tiab] OR "posttraumatic stress"[tiab] OR "post-traumatic stress"[tiab] OR suicid*[tiab] ) AND ( "Health Services Accessibility"[Mesh] OR "Health Services Needs and Demand"[Mesh] OR "Patient Acceptance of Health Care"[Mesh] OR access*[tiab] OR utiliz*[tiab] OR utilis*[tiab] OR "service use"[tiab] OR "services use"[tiab] OR "use of service*"[tiab] OR "healthcare use"[tiab] OR "health care use"[tiab] OR "help seek*"[tiab] OR helpseek*[tiab] OR "care seek*"[tiab] OR "treatment seek*"[tiab] OR uptake[tiab] OR referral*[tiab] OR "treatment gap*"[tiab] OR barrier*[tiab] OR facilitator*[tiab] OR "unmet need*"[tiab] ) AND ( "Infant"[Mesh] OR "Child"[Mesh] OR "Adolescent"[Mesh] OR infant*[tiab] OR child*[tiab] OR adolescen*[tiab] OR youth*[tiab] OR teen*[tiab] OR pediatric*[tiab] OR paediatric*[tiab] OR "young people"[tiab] OR "young person*"[tiab] OR "Pregnancy"[Mesh] OR "Pregnant Women"[Mesh] OR "Postpartum Period"[Mesh] OR pregnan*[tiab] OR antenatal[tiab] OR prenatal[tiab] OR perinatal[tiab] OR peripartum[tiab] OR postpartum[tiab] OR postnatal[tiab] OR puerper*[tiab] OR maternal[tiab] OR "new mother*"[tiab] OR "Aged"[Mesh] OR "Aged, 80 and over"[Mesh] OR "older adult*"[tiab] OR "older person*"[tiab] OR "older people"[tiab] OR "older patient*"[tiab] OR elder*[tiab] OR geriatric*[tiab] OR senior*[tiab] OR "late life"[tiab] OR "late-life"[tiab] ) ) AND 2026/02/18:3000[crdt]

The framework terms search title/abstract fields. They neither restrict retrieval to papers citing Levesque et al. (2013) nor establish substantive framework use. Framework eligibility remains a human full-text decision. Forward citation tracking is documented separately in S5.1, S5.3, and S5.8.

# S5.8 Web of Science search-history record

Sources: Search history.pdf, exported 24 September 2026, and the complete youth and older-adult queries supplied and confirmed by the author on 24 September 2026. The historical entries document Web of Science Core Collection with A&HCI, ESCI, SCI-EXPANDED, and SSCI. On 1 August 2026, the DOI seed 10.1186/1475-9276-12-18 retrieved the Levesque et al. (2013) article at 17:48; its citing-document set contained 2,423 records at 17:49. The population filters returned 185 youth hits at 17:49, 48 perinatal hits at 19:48, and 67 older-adult hits at 19:59 (times as displayed, converted to 24-hour notation). These are the 300 route-specific hits in S5.1–S5.3, before cross-filter deduplication.

All three citation-route queries are reproduced in full below, with whitespace normalized. The author-supplied strings complete the youth and older-adult entries that were clipped in the PDF; the perinatal entry is fully visible in that source. The interface label Search within all fields is retained unchanged.

## Youth — 1 August 2026, 17:49; 185 hits

Citations of Patient-centred access to health care: conceptualising access at the interface of health systems and populations and (Child* Or Adolescen* Or Youth* Or "Young People" Or Pediatric* Or Paediatric* Or Kind* Or Jugendlich*) And ("Mental Health" Or "Mental Health Care" Or "Mental Healthcare" Or "Mental Illness*" Or "Mental Disorder*" Or Psychiatr* Or Psycholog* Or Psychisch* Or Depress* Or Anxi* Or Suicid* Or "Self-harm" Or Trauma* Or Ptsd Or Adhd Or Autism Or Neurodevelopment* Or Psychosis Or Schizophren* Or Bipolar Or "Eating Disorder*" Or "Substance Use") (Search within all fields)

The author confirmed this as the youth citation-route query associated with 185 hits; it is distinct from the DOI-plus-keyword Smart Search attempt documented below.

## Perinatal — 1 August 2026, 19:48; 48 hits

Citations of Patient-centred access to health care: conceptualising access at the interface of health systems and populations and (Pregnan* Or Perinatal Or Antenatal Or Prenatal Or Postpart* Or Matern* Or Schwanger* Or Wochenbett) And ("Mental Health" Or "Mental Health Care" Or "Mental Healthcare" Or "Mental Illness*" Or "Mental Disorder*" Or Psychiatr* Or Psycholog* Or Psychisch* Or Depress* Or Anxi* Or Suicid* Or Trauma* Or Ptsd) (Search within all fields)

Complete query visible in the historical PDF, including the field label.

## Older adults — 1 August 2026, 19:59; 67 hits

Citations of Patient-centred access to health care: conceptualising access at the interface of health systems and populations and ("Older Adult*" Or Elder* Or Geriatric* Or Ageing Or Aging Or "Later Life" Or "65 Years" Or Ältere* Or Hochbetagt* Or Geront*) And ("Mental Health" Or "Mental Health Care" Or "Mental Healthcare" Or "Mental Illness*" Or "Mental Disorder*" Or Psychiatr* Or Psycholog* Or Psychisch* Or Depress* Or Anxi* Or Suicid* Or Dementia Or Cognitive Or Psychosis Or Schizophren* Or Bipolar) (Search within all fields)

The author confirmed this complete query for 1 August at 19:59. Its filter is identical to the complete older-adult entry shown on 31 July at 09:59 (67 hits); that earlier output is not counted again.

## Separate historical Smart Search attempt — 1 August 2026, 17:49; 0 hits

10.1186/1475-9276-12-18 and (Child* Or Adolescen* Or Youth* Or "Young People" Or Pediatric* Or Paediatric* Or Kind* Or Jugendlich*) And ("Mental Health" Or "Mental Health Care" Or "Mental Healthcare" Or "Mental Illness*" Or "Mental Disorder*" Or Psychiatr* Or Psycholog* Or Psychisch* Or Depress* Or Anxi* Or Suicid* Or "Self-harm" Or Trauma* Or Ptsd Or Adhd Or Autism Or Neurodevelopment* Or Psychosis Or Schizophren* Or Bipolar Or "Eating Disorder*" Or "Substance Use") (Search within all fields)

This DOI-plus-keyword string uses the same youth keyword block as the citation filter. It belongs to the historical zero-hit Smart Search attempt and adds neither a separate identification route nor candidates to the reported flow.

The 31 July history also contains a 2,419-record citing set and preceding runs, including a 25-hit youth run. These historical outputs have not been added to the 300-hit citation route or the identification flow.
