# Supplementary material for: Patient-centered access to mental health care at selected life-course stages: a scoping review using the Levesque framework
Source: BMC Health Serv Res. 2026 Oct 22;26:1464. doi: 10.1186/s12913-026-15895-z (PMC13693167; doi:10.1186/s12913-026-15895-z)
Supplement: Supplementary file 12 [file 12913_2026_15895_MOESM9_ESM.docx]

Supplementary Material S6

# Complete evidence matrix

**Patient-centered access to mental health care at selected life-course stages: a scoping review using the Levesque framework**

This matrix describes the 37 sources in the scoping review, their identification routes, methodological designs, evidence roles and contributions to the Levesque-based synthesis. Item-level appraisal and agreement statistics are reported in Supplementary Table S1.

Source type is distinct from evidence role. Ten direct sources (eight empirical studies and two reviews) inform the core population-specific synthesis. Adjacent and contextual sources support explicitly identified interpretation; the protocol and conceptual papers are not treated as completed empirical evidence. Directness concerns population, topic and framework eligibility and does not necessarily indicate first-person reporting by service users.

JCC assessed eligibility, assigned evidence roles and conducted Levesque coding. BR independently checked extracted study data against full texts. JCC and BR independently appraised methodological quality and resolved remaining disagreements by consensus. Appraisal categories below reflect the final source-level decisions in Supplementary Table S1. Identification routes refer to the available source inventory; they do not constitute a complete historical screening log. Williams-Ridgway (E35) was retrieved through multiple routes, including the PubMed update; its adjacent classification and single entry in the 37-source map are unchanged.

# S6A Source inventory and evidence roles

ELICIT-01 = perinatal report; ELICIT-02 = older-adult report; ELICIT-03 = broader mental-health report (all 18 February 2026); ELICIT-04 = dedicated youth report (31 July 2026). Search procedures and route-level yields are documented in Supplementary Materials S3–S5.

| **ID** | **Source** | **Population or domain** | **Source type and design** | **Identification route** | **Evidence role and eligibility basis** | **Appraisal** |
| --- | --- | --- | --- | --- | --- | --- |
| E01 | Levesque et al. 2013 | Framework | Conceptual framework paper | Seed publication for citation tracking | Framework-level / conceptual. Originating framework; not empirical evidence. | Not appraised |
| E02 | Ash et al. 2026 | Perinatal | Qualitative primary study | ELICIT-01 (perinatal) | Direct empirical. Meets all direct criteria. | MMAT qualitative |
| E03 | Salameh et al. 2025 | Perinatal | Qualitative primary study | ELICIT-01 (perinatal) | Direct empirical. Meets all direct criteria. | MMAT qualitative |
| E04 | Harper et al. 2018 | Youth / young adults | Conceptual commentary | ELICIT-03 (broader mental health) | Contextual conceptual. No completed empirical findings; mixed adolescent/young-adult scope. | Not appraised |
| E05 | Martínez-Jaime et al. 2024 | Youth | Retrospective cohort / quantitative non-randomized primary study | ELICIT-03; ELICIT-04; forward citation tracking | Direct empirical. Meets all direct criteria. | MMAT quantitative non-randomized |
| E06 | Schwarz et al. 2022 | Older adults | Qualitative primary study | ELICIT-02 (older adults) | Direct empirical. Eligible older-adult mental-illness subgroup reported within broader study. | MMAT qualitative |
| E07 | Sambrook Smith et al. 2019 | Perinatal | Systematic review / qualitative meta-synthesis | ELICIT-01 (perinatal) | Adjacent review-level. Perinatal mental-health relevance but not a substantive Levesque application meeting direct criteria. | JBI review checklist |
| E08 | Webb et al. 2023 | Perinatal | Meta-review | ELICIT-01 (perinatal) | Adjacent review-level. Broad contextual synthesis; not a direct substantive Levesque application. | JBI review checklist |
| E09 | Cu et al. 2021 | Framework / general health care | Scoping review | Predefined contextual/manual source | Framework-level contextual review. General health-care applications rather than target-population mental-health evidence. | JBI review checklist |
| E10 | Lowther-Payne et al. 2023 | Adult mental health | Systematic mapping review | Predefined contextual/manual source | Framework-level contextual review. Adult UK mental-health evidence, not one of the three target populations. | JBI review checklist |
| E11 | Werlen et al. 2020 | Youth | Systematic review and meta-analysis of 34 RCTs | ELICIT-04; forward citation tracking | Adjacent review-level. Eligibility extended to under 19 years and mixed samples rather than strict 0–17. | JBI review checklist |
| E12 | Barbo & Petrucka 2024 | Youth refugees | Integrative review | ELICIT-04 | Adjacent review-level. Youth definition extended beyond the prespecified 0–17 population. | JBI review checklist |
| E13 | de Jong-Bardelmeijer et al. 2026 | Perinatal / postpartum | Cross-sectional quantitative observational primary study | ELICIT-01 (perinatal) | Adjacent empirical. General postpartum health-care utilization rather than mental-health-specific access. | MMAT quantitative non-randomized |
| E14 | Viveiros & Darling 2018 | Perinatal | Qualitative primary study | Forward citation tracking | Direct empirical. Meets all direct criteria. | MMAT qualitative |
| E15 | Marshman et al. 2023 | Perinatal | Rapid review | Forward citation tracking | Direct review-level. Meets all direct review criteria. | JBI review checklist |
| E16 | DeRoche et al. 2023 | Perinatal | Qualitative primary study | ELICIT-01 (perinatal) | Adjacent empirical. Relevant perinatal mental-health evidence but no substantive direct Levesque application. | MMAT qualitative |
| E17 | Koire et al. 2024 | Perinatal | Quantitative observational workforce study | ELICIT-01 (perinatal) | Adjacent empirical. Workforce availability study; no substantive direct Levesque application. | MMAT quantitative non-randomized |
| E18 | Blackmore et al. 2022 | Perinatal | Mixed-methods development project; integration report with linked companion reports | ELICIT-01 (perinatal) | Adjacent empirical. Implementation evidence; substantive Levesque application unclear; linked reports do not constitute an additional independent sample. | MMAT mixed methods and components |
| E19 | Fisher et al. 2024 | Perinatal | Qualitative realist evaluation report | ELICIT-01 (perinatal) | Adjacent empirical. Engagement-focused contextual evidence rather than direct substantive Levesque application. | MMAT qualitative |
| E20 | Lewkowitz et al. 2024 | Perinatal | Systematic review and meta-analysis | ELICIT-01 (perinatal) | Adjacent review-level. Intervention review without substantive Levesque application. | JBI review checklist |
| E21 | Bali et al. 2024 | Perinatal / migrant maternity care | Qualitative meta-synthesis | ELICIT-01 / contextual route | Adjacent review-level. Maternity-care rather than mental-health-specific access. | JBI review checklist |
| E22 | Bartlett et al. 2025 | Dementia / older adults | Mixed-methods primary study | ELICIT-02 (older adults) | Adjacent empirical. Dementia/social-care setting rather than conventional mental-health service access. | MMAT mixed methods |
| E23 | Dableh et al. 2025 (co-design) | Older adults | Qualitative co-design study with embedded prioritization and voting | ELICIT-02 (older adults) | Adjacent empirical. Primary-care implementation rather than mental-health-service access. | MMAT qualitative |
| E24 | Dableh et al. 2025 (qualitative inquiry) | Older adults | Qualitative primary study | ELICIT-02 (older adults) | Adjacent empirical. Primary-care rather than mental-health-service access. | MMAT qualitative |
| E25 | Kurpas et al. 2018 | Older adults | Qualitative framework analysis | ELICIT-02 (older adults) | Adjacent empirical. General frail-older-adult health/social care interface. | MMAT qualitative |
| E26 | Ramakrishnan & Malhotra 2025 | Dementia / older adults | Qualitative primary study | ELICIT-02 (older adults) | Adjacent empirical. Dementia/community-care rather than direct older-adult mental-health-service access. | MMAT qualitative |
| E27 | Daskalska et al. 2025 | Youth | Qualitative primary study | Forward citation tracking | Direct empirical. Meets all direct criteria. | MMAT qualitative |
| E28 | Daskalska et al. 2024 | Youth | Mixed-methods program evaluation | Forward citation tracking | Direct empirical. Meets all direct criteria; providers report target-population access. | MMAT mixed methods |
| E29 | Adams 2024 | Youth | Quantitative descriptive survey | Forward citation tracking | Direct empirical. Meets all direct criteria. | MMAT quantitative descriptive |
| E30 | Berger et al. 2020 | Perinatal | Qualitative study protocol | ELICIT-01 (perinatal) | Protocol evidence. No completed empirical findings. | Not appraised |
| E31 | Viveiros & Darling 2019 | Perinatal | Scoping review | ELICIT-01 (perinatal) | Direct review-level. Meets all direct review criteria. | JBI review checklist |
| E32 | Kingston et al. 2014 | Perinatal | Cross-sectional observational survey | ELICIT-01 (perinatal) | Adjacent empirical. Relevant screening acceptability evidence but not substantive Levesque application. | MMAT quantitative non-randomized |
| E33 | Zivin et al. 2024 | Perinatal | Quantitative observational / administrative-data study | ELICIT-01 (perinatal) | Adjacent empirical. Affordability/realized-use context without substantive Levesque application. | MMAT quantitative non-randomized |
| E34 | Fathi Afshar 2019 | Refugee minors | Qualitative master’s thesis | ELICIT-04 human follow-up | Adjacent empirical. General health-care rather than mental-health-specific access. | MMAT qualitative |
| E35 | Williams-Ridgway et al. 2026 | Youth / young adults | Qualitative primary study | Thematic PubMed update (28 Jul 2026; PMID 41709297); ELICIT-04 human follow-up; forward citation tracking | Adjacent empirical. Target population aged 10–25 without separate 0–17 findings. | MMAT qualitative |
| E36 | Yılmaz et al. 2025 | Forcibly displaced youth | Overview of reviews | ELICIT-04 human follow-up; forward citation tracking | Adjacent review-level. Age range extended to approximately 24 years; no consistently separate 0–17 synthesis. | JBI review checklist |
| E37 | Mejía-Grueso et al. 2025 | Older adults | Quantitative descriptive inequalities study | ELICIT-02 (older adults) | Adjacent empirical. General health-service inequalities rather than mental-health-specific access. | MMAT quantitative descriptive |

# S6B Framework use and contribution to the synthesis

Article titles are abbreviated where needed. The full citations are provided in the manuscript. “Aligned” denotes conceptual correspondence and does not establish substantive framework use sufficient for direct classification. Dimensions and contributions describe what each source adds; they are not pooled estimates of barrier frequency.

| **ID and source** | **Article title or abbreviated title** | **Mental-health and access focus** | **Levesque framework use** | **Dimensions represented** | **Contribution to the synthesis** |
| --- | --- | --- | --- | --- | --- |
| E01 Levesque et al. 2013 | Patient-centred access to health care: conceptualising access at the interface of health systems and populations | General health-care access framework | Originating definition of five service dimensions and five corresponding population abilities | All ten dimensions | Defines the analytical framework; not used as population-specific evidence. |
| E02 Ash et al. 2026 | Barriers and Facilitators to Accessing Mental Health Supports Among Black Perinatal Women | Mental-health supports among Black perinatal women | Framework informed conceptualisation and analysis | Multiple dimensions; especially acceptability, ability to seek, appropriateness | Medical mistrust, stigma, screening transparency, and culturally responsive trauma-informed care. |
| E03 Salameh et al. 2025 | Syrian refugee women’s experiences of barriers to mental health services for postpartum depression | Postpartum-depression service access among Syrian refugee women | Supply-side dimensions used in thematic analysis | Approachability, acceptability, availability / accommodation, affordability, appropriateness | Knowledge gaps, stigma, language/cultural mismatch, transport, childcare, time constraints, and screening gaps. |
| E04 Harper et al. 2018 | Using the Social-Ecological Model to Improve Access to Care for Adolescents and Young Adults | Adolescent and young-adult access | Levesque used as supplementary conceptual model alongside social-ecological model | Selected dimensions | Confidentiality, youth-friendly care, schools, parental roles, and declining contact in young adulthood. |
| E05 Martínez-Jaime et al. 2024 | Access to early diagnosis for ADHD among children and adolescents in Mexico City | ADHD diagnostic pathway in children/adolescents aged 0–17 | Access Pathway and AAcCeDa instrument derived from Levesque | All five dimension-ability pairs, with strong demand-side operationalisation | Caregiver-reported diagnostic pathway among already diagnosed hospital attendees: diagnostic delay, observed sex differences, teacher recognition, waits and fragmented referral. People who never reached diagnosis were not represented. |
| E06 Schwarz et al. 2022 | Barriers to accessing health care for people with chronic conditions: older people with pre-existing mental illness case group | Access for independently living adults aged >65 with pre-existing mental illness; dementia excluded | A priori use of Levesque dimensions and abilities | All ten dimensions | Stakeholder accounts and two service-user interviews identified limited perception of need, stigma, service shortages, poor coordination, financial burden and the importance of continuity and trust. |
| E07 Sambrook Smith et al. 2019 | Barriers to accessing mental health services for women with perinatal mental illness: systematic review and meta-synthesis | Perinatal mental-health service access | Closely aligned with Levesque concepts; framework not primary analytic structure | Multiple dimensions | Synthesises UK qualitative evidence on barriers and mechanisms across the perinatal pathway. |
| E08 Webb et al. 2023 | Meta-review of barriers and facilitators to women accessing perinatal mental healthcare | Perinatal mental-health access | Conceptually aligned | Multiple dimensions | Review-level context on recurrent barriers and facilitators. |
| E09 Cu et al. 2021 | Assessing healthcare access using the Levesque conceptual framework: a scoping review | Empirical applications of Levesque in health care | Synthesises a priori and a posteriori uses of the framework | All ten dimensions | Methodological benchmark showing uneven operationalisation and limited paired measurement. |
| E10 Lowther-Payne et al. 2023 | Understanding inequalities in access to adult mental health services in the UK: a systematic mapping review | Adult mental-health access measurement and inequalities | Levesque used to classify access measures and barriers | All pathway stages | Shows emphasis on utilization/health-care seeking and limited measurement of perception, desire for care, and consequences. |
| E11 Werlen et al. 2020 | Interventions to improve children’s access to mental health care: systematic review and meta-analysis | Interventions to improve access and engagement | Outcomes and intervention types mapped to Levesque dimensions | Mainly approachability / ability to perceive and appropriateness / ability to engage | School interventions improved knowledge more consistently than service use; reminders and motivational strategies improved first attendance. |
| E12 Barbo & Petrucka 2024 | Barriers associated with inaccessible mental health services for youth refugees and asylum seekers | Mental-health service access among youth refugees/asylum seekers | Deductive coding using all five dimension-ability pairs | All ten dimensions | Low literacy, stigma, cultural/linguistic mismatch, restrictive service rules, waits, costs, and limited provider competence. |
| E13 de Jong-Bardelmeijer et al. 2026 | Prevalence and determinants of health-care utilization among Dutch women postpartum | Postpartum health-care utilization | Levesque-derived determinants combined with Andersen model | Mainly demand-side / approachability-related determinants | Unawareness of available services associated with lower utilization; illustrates quantitative determinants rather than validated Levesque measurement. |
| E14 Viveiros & Darling 2018 | Barriers and facilitators of accessing perinatal mental health services: women receiving continuity-of-care midwifery | Perinatal mental-health services | Levesque-informed analysis of barriers/facilitators | Multiple dimensions | Stigma/fear, broken referrals, distance, limited capacity, baby-centredness, six-week discharge, and cost; continuity, community, advocacy, and informed choice facilitated access. |
| E15 Marshman et al. 2023 | Barriers to perinatal mental health care experiences by midwives and obstetricians and their patients: a rapid review | Perinatal mental-health access across patient and professional perspectives | Barriers mapped to Levesque supply-side dimensions | Five service dimensions with pathway implications | Overlapping detection/referral barriers; stigma at societal, institutional, and individual levels. |
| E16 DeRoche et al. 2023 | Examining gaps in perinatal mental health care: provider perspectives in Canada | Provider-reported gaps in perinatal mental-health care | Closely aligned | Approachability, availability, appropriateness | Limited training, inconsistent/non-mandated screening, referral and service gaps. |
| E17 Koire et al. 2024 | Prevalence of community perinatal psychiatrists in the United States | Availability of specialist perinatal psychiatry | Indirect alignment | Availability / accommodation | Documents specialist workforce shortages and geographic availability constraints. |
| E18 Blackmore et al. 2022 | Introducing and integrating perinatal mental health screening: equity-informed development study | Perinatal mental-health screening and integration | Closely aligned | Approachability, acceptability, appropriateness | Equity-informed screening and referral integration in a linked mixed-methods project. The integration report is not treated as a new independent sample; component appraisal uses companion empirical reports. |
| E19 Fisher et al. 2024 | Women’s engagement with community perinatal mental health services: a realist evaluation | Engagement with community perinatal mental-health services | Closely aligned | Appropriateness / ability to engage | Relationship-centred care and mechanisms affecting sustained engagement. |
| E20 Lewkowitz et al. 2024 | Digital health interventions for postpartum depression or anxiety: systematic review and meta-analysis | Digital interventions for postpartum depression/anxiety | Conceptually aligned | Availability / reach and engagement | Digital access and engagement context; intervention effects rather than framework application. |
| E21 Bali et al. 2024 | Barriers and facilitators of maternity-care access for African-born women in Australia: meta-synthesis | Maternity-care access during pregnancy/postpartum | Levesque-informed synthesis | Multiple dimensions | Culturally unsafe communication, discrimination, limited information, mistrust, navigation difficulties, waits, and discontinuity. |
| E22 Bartlett et al. 2025 | Access to social farms for people with dementia living at home in England | Access to social farms for people with dementia | Levesque used as an a priori navigational guide | Multiple dimensions | Travel and carer dependence; intersectionality and within-service access. |
| E23 Dableh et al. 2025 (co-design) | Maximizing older people’s access to primary health-care centers in Lebanon | Primary-care access and screening prototypes | Levesque-aligned; explicit substantive framework use unclear in this report | Selected dimensions | Prototypes for depression/cognitive screening and awareness campaigns. |
| E24 Dableh et al. 2025 (qualitative inquiry) | Barriers and enablers to primary health-care center access for older people in Lebanon | Primary-care access for older adults | A priori use of all dimensions and abilities | All ten dimensions | Information, transport, mobility, finance, provider attitudes, cognitive/sensory limitations, and continuity. |
| E25 Kurpas et al. 2018 | Patient-centred access to health care: framework analysis of the care interface for frail older adults | Health/social-care access among frail older adults | A posteriori use of all dimensions and abilities | All ten dimensions | Psychological-support gap, staff shortages, waits, remote clinics, costs, and need for health-social-care integration. |
| E26 Ramakrishnan & Malhotra 2025 | Challenges to accessing community dementia care services: a qualitative study | Community dementia-care access | Levesque-informed/closely aligned | Multiple dimensions | Caregivers and professionals described navigation, service availability, family dependence and continuity challenges; people with dementia were not interviewed directly. |
| E27 Daskalska et al. 2025 | Access to mental health care for Black and Latino teens with anxiety and depression | Mental-health access among Black and Latino teens aged 14–17 | Themes interpreted through an adapted Levesque access model | Multiple dimensions | One adolescent, three parents and six professionals described first contacts, provider preferences, culturally appropriate, trauma-informed and family-centered care, community awareness and service availability. |
| E28 Daskalska et al. 2024 | Closing the gap in access to child mental health care: Wisconsin consultation program | Child mental-health consultation model in primary care | Qualitative themes categorised with adapted Levesque framework | Multiple dimensions | Anonymous, unlinked provider surveys showed higher self-reported confidence and skill in the follow-up sample, with persistent referral, waiting-time and insurance barriers. The design does not establish improved patient access or a causal program effect. |
| E29 Adams 2024 | Availability and Accessibility of Mental Health Services for Youth in Safety-Net Health Centers | Availability/accessibility of youth outpatient mental-health care | Patient-centered access framework informed study variables | Availability / accommodation, affordability, approachability | Facility-level survey identified closures or non-provision, long waits despite staffing, insurance and sliding-scale conditions, and inaccurate or outdated directories; no first-person youth access accounts. |
| E30 Berger et al. 2020 | Perinatal mental health care from the user and provider perspective: study protocol | Planned perinatal mental-health access study | Interview guides and planned analysis informed by all ten dimensions | All ten dimensions (planned) | Documents planned application; not evidence of observed barriers or facilitators. |
| E31 Viveiros & Darling 2019 | Perceptions of barriers to accessing perinatal mental health care in midwifery: a scoping review | Perinatal mental-health access in midwifery | Barriers mapped onto all ten dimensions | All ten dimensions | Early pathway barriers prominent, particularly approachability and ability to perceive need. |
| E32 Kingston et al. 2014 | Public views of acceptability of perinatal mental-health screening and treatment preference | Acceptability of perinatal screening/treatment preferences | Closely aligned | Acceptability / ability to seek | Preferences, acceptability, and conditions supporting screening uptake. |
| E33 Zivin et al. 2024 | Perinatal psychotherapy use and costs before and after mandated insurance coverage | Psychotherapy use and cost around insurance coverage | Indirect alignment | Affordability / ability to pay and realized use | Insurance coverage, psychotherapy utilization, costs, and treatment intensity. |
| E34 Fathi Afshar 2019 | Access to health care for refugee children and unaccompanied minor aliens in the Netherlands | General health-care access among refugee minors, including mental-care needs | Provider interviews deductively analysed with Levesque | All five service dimensions; linked abilities in interpretation | Health literacy, cultural understanding, communication, coordination, human-resource shortages, and timely access. |
| E35 Williams-Ridgway et al. 2026 | Minoritised ethnic young people’s access to eating-disorder services in the UK | Specialist eating-disorder service access | Interview schedule informed by Levesque; service/user factors interpreted through the framework | Multiple dimensions | Gatekeeping, service invisibility, locations, language/cultural mismatch, workforce diversity/training, awareness, shame, and stigma. |
| E36 Yılmaz et al. 2025 | Forcibly displaced children and youth’s access to mental-health support: overview of reviews | Mental-health access and engagement among forcibly displaced children/youth | Narrative synthesis organised by the five-stage Levesque model | All pathway stages | Low literacy, stigma, limited autonomy, legal insecurity, unstable housing, weak support, fragmentation, and lack of culturally/linguistically responsive care. |
| E37 Mejía-Grueso et al. 2025 | Inequalities in access to health services among older adults in Colombia, 2015–2023 | Health-service access inequalities among older adults | Indirect alignment with access dimensions | Affordability / ability to pay and equity determinants | Socioeconomic inequalities in access; contextual evidence for affordability and structural inequity. |

# S6C Source counts

| **Source type** | **n** |
| --- | --- |
| Empirical primary-study entries | 23 |
| Reviews or evidence syntheses | 11 |
| Study protocol | 1 |
| Conceptual or framework sources | 2 |
| Total sources | 37 |

| **Evidence role** | **n** |
| --- | --- |
| Direct empirical | 8 |
| Direct review-level | 2 |
| Adjacent empirical | 15 |
| Adjacent review-level | 7 |
| Framework-level contextual reviews | 2 |
| Framework-level conceptual paper | 1 |
| Contextual conceptual commentary | 1 |
| Protocol evidence | 1 |
| Total sources | 37 |

The direct set contains five perinatal sources (three empirical studies and two reviews), four youth empirical studies and one older-adult empirical study. The 34 appraised sources comprise the 23 empirical entries and 11 reviews. The three mixed-methods component appraisals do not add sources or independent samples.

# S6D Respondent perspectives in selected youth and older-adult sources

These source-specific examples support the discussion of whose account is represented. They are not based on systematic respondent-type coding of every mapped source and do not establish how frequently proxy reporting occurs in one population compared with another.

| **ID and source** | **Evidence role** | **Respondents or data source** | **Interpretive implication** |
| --- | --- | --- | --- |
| E05 Martínez-Jaime et al. 2024 | Direct youth empirical | 177 child/adolescent–caregiver dyads with diagnosed ADHD; pathway ages reported retrospectively by caregivers. | Diagnostic-pathway findings concern caregiver recall among people who reached a tertiary hospital and diagnosis. |
| E27 Daskalska et al. 2025 | Direct youth empirical | 10 interviewees: one adolescent, three parents and six professionals. | The analysis combines first-person youth, parent and professional perspectives; it is not a broad sample of adolescent accounts. |
| E28 Daskalska et al. 2024 | Direct youth empirical | Anonymous, unlinked provider surveys at baseline (n = 342) and follow-up (n = 114), including free-text responses. | Provider confidence and perceived barriers are not direct measures of patient experience or causal changes in access. |
| E29 Adams 2024 | Direct youth empirical | Facility-level survey of safety-net health centers. | Organizational availability and accessibility were assessed, rather than first-person youth experience. |
| E06 Schwarz et al. 2022 | Direct older-adult empirical | Mental-illness case group: 10 interviewees, comprising two service users, two carers, five providers and one researcher; intended population aged >65; dementia excluded. | The only direct older-adult source combines predominantly stakeholder perspectives with two service-user accounts. |
| E26 Ramakrishnan and Malhotra 2025 | Adjacent dementia empirical | Caregiver and professional interviews; people with dementia were not interviewed directly. | Accounts concern care navigation and service provision from caregiver and professional perspectives. |
| E22 Bartlett et al. 2025 | Adjacent dementia empirical | Survey of 32 social-farm managers, four online focus groups, and two individual plus twelve dyadic interviews with people living with dementia and supporters. | Direct and supported participation must be distinguished from exclusively proxy-reported evidence. |
